# A Mesoporous Calcium Peroxide Nanocuboid with High Tumor Accumulation Across Biological Barriers for High Efficacy Tumor Therapy

**DOI:** 10.1002/advs.202510778

**Published:** 2025-07-20

**Authors:** Qingdeng Fan, Min Wang, Jie Lin, Ya Huang, Jing Yang, Jiaoyang Zhu, Bin Ren, Li Sun, Zongheng Li, Aochi Liu, Wei Xiong, Zhenni Wei, Lin Huang, Chenggong Yan, Ge Wen, Zhao Chen, Xiaoyuan Chen, Zheyu Shen

**Affiliations:** ^1^ School of Biomedical Engineering Southern Medical University 1023 Shatai South Road, Baiyun Guangzhou Guangdong 510515 P. R. China; ^2^ Medical Imaging Center Nanfang Hospital Southern Medical University 1023 Shatai South Road, Baiyun Guangzhou Guangdong 510515 P. R. China; ^3^ Ningbo Institute of Materials Technology and Engineering CAS 1219 Zhongguan West Road Ningbo 315201 P. R. China; ^4^ Departments of Diagnostic Radiology, Surgery, Chemical and Biomolecular Engineering, and Biomedical Engineering Clinical Imaging Research Centre Nanomedicine Translational Research Program Yong Loo Lin School of Medicine and College of Design and Engineering National University of Singapore Singapore 119228 Singapore

**Keywords:** enhanced drug delivery across biological barriers, magnetic resonance imaging (MRI), mesoporous calcium peroxide nanocuboid (MCPNC), positive feedback loop of Fenton reaction, targeted therapy for tumors

## Abstract

The common problem of tumor therapy based on nanoparticles is the limited efficacy due to the blockage of tumor accumulation by biological barriers. To enhance the drug delivery of nanoparticles across biological barriers and augment their tumor accumulation, herein, a mesoporous calcium peroxide nanocuboid (MCPNC) is developed via a facile hydrolysis‐precipitation method, which can be utilized for high efficacy tumor therapy by promoting a positive feedback loop of Fenton reaction. The biodistribution results demonstrate that MCPNC exhibits higher accumulation in various tissues than calcium peroxide nanosphere (CPNS). Ferroheme (FH) and exceedingly small magnetic iron oxide nanoparticle (IO) loaded MCPNC is modified with hyaluronic acid (HA), forming MCPNC‐FH‐IO@HA. Under acidic tumor microenvironment (TME), Fe^3+/2+^, H_2_O_2_ and Ca^2+^ can be released from MCPNC‐FH‐IO@HA. The reactive oxygen species (ROS) generation through Fenton reaction can disrupt mitochondrial membranes, which accelerates the unbalance of Ca^2+^ mitochondrial homeostasis. The loss of mitochondrial membrane potential activates mitochondrial autophagy, which results in the release of Fe^3+/2+^ in tumor mitochondria. The released Fe^3+/2+^ can further produce more and more ROS via the Fenton reaction, which establishes the positive feedback loop of the Fenton reaction. Both in vitro and in vivo results demonstrate that MCPNC‐FH‐IO@HA exhibits remarkable antitumor efficacy, superior MRI performance, and favorable biosafety.

## Graphical abstract

The ferroheme (FH) and exceedingly small magnetic iron oxide nanoparticles (IO) loaded mesoporous calcium peroxide nanocuboid (MCPNC) with conjugation of hyaluronic acid (HA) possesses exceptional capability of tumor accumulation, and enhances therapeutic efficacy of tumors based on the mechanism of promoting a positive feedback loop of Fenton reaction via reactive oxygen species (ROS) generation and mitophagy under acidic tumor microenvironment (TME).

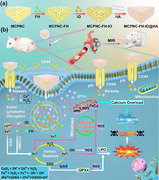

## 1 Introduction

Nanomedicines hold significant importance for various biomedical applications due to their capacity to prolong blood circulation time, overcome biological barriers, and reduce toxic and side effects by designing their physicochemical parameters, e.g., surface charge, shape, size, and surface functionalization.^[^ ^1^, ^2^, ^3^ ^]^ Numerous studies have demonstrated that the physicochemical properties of nanomedicines exert a profound influence on their functionalities.^[^ ^4^, ^5^, ^6^ ^]^ For instance, among the common nanoparticle shapes, nanocuboids (NCs), which are akin to disk‐shaped nanoparticles, possess their unique tumbling and margination dynamics.^[^ ^7^ ^]^ Compared with nanospheres (NSs) and nanorods (NRs), NCs present stronger interactions with the vascular wall and a more efficient penetration through the vascular endothelium into tissues.^[^ ^2^, ^4^, ^7^, ^8^, ^9^ ^]^ Therefore, NCs should be a superior drug carrier for effective accumulation in tumor sites, achieving better therapeutic outcomes. However, most of the current research focuses on NSs, and there is an urgent need for further development and investigation on NCs.

Among the myriad of bio‐imaging techniques, magnetic resonance imaging (MRI) has been extensively used in the clinical diagnosis of diseases due to its non‐destructive nature, excellent tissue discriminability, high‐fidelity spatial resolution, and multi‐layered tissue penetration.^[^ ^10^, ^11^, ^12^ ^]^ Gadolinium chelates have been dominating the market of MRI contrast agents (CAs) for decades.^[^ ^13^ ^]^ However, gadolinium‐based CAs (GBCAs) still exhibit numerous side effects, including nephrotoxicity and brain deposition. The emerging exceedingly small magnetic iron oxide nanoparticles (IO, < 5.0 nm) are expected to replace GBCAs owing to their high longitudinal relaxivity (*r*_1_) and great biosafety. Nevertheless, the specific diagnostic capabilities of IO for diseased tissues still need to be enhanced.

Currently, chemodynamic therapy (CDT) based on the Fenton reaction has been extensively employed for tumor treatments.^[^ ^14^, ^15^, ^16^ ^]^ This strategy typically involves the generation of highly lethal hydroxyl radicals (·OH) in tumors through Fenton or Fenton‐like reactions, which subsequently induce ferroptosis of tumor cells.^[^ ^17^, ^18^, ^19^ ^]^ However, the relatively low concentration of Fenton reagents (e.g., Fe^2+^, Cu^2+^, or Mn^2+^) and the limited content of H_2_O_2_ in the tumor microenvironment (TME) are adverse to the generation of reactive oxygen species (ROS) via Fenton or Fenton‐like reactions. Furthermore, glutathione peroxidase 4 (GPX4), which is overexpressed in tumor cells with glutathione (GSH) serving as its substrate, eliminates toxic ROS to prevent cell damage induced by oxidative stress,^[^ ^20^ ^]^ thereby significantly compromising the efficacy of CDT. Therefore, designing a peroxide‐based nanoplatform, which can promote the Fenton reaction to generate a large amount of ROS to exceed the defensive capacity of the antioxidant system in tumors, holds significant investigation value for cancer therapy.

Ca^2+^ is an important factor in regulating mitochondrial homeostasis, which is essential for regulating the intracellular antioxidant system.^[^ ^21^, ^22^, ^23^ ^]^ Besides, calcium peroxide nanoparticle (CPNP) is crucial for enhancing the efficiency of the Fenton reaction as a TME‐responsive self‐supplied H_2_O_2_ carrier.^[^ ^24^, ^25^, ^26^ ^]^ Therefore, CPNP is an ideal choice for disrupting mitochondrial homeostasis to induce intracellular oxidative stress, and thereby improving the efficacy of CDT.

**Scheme 1:**
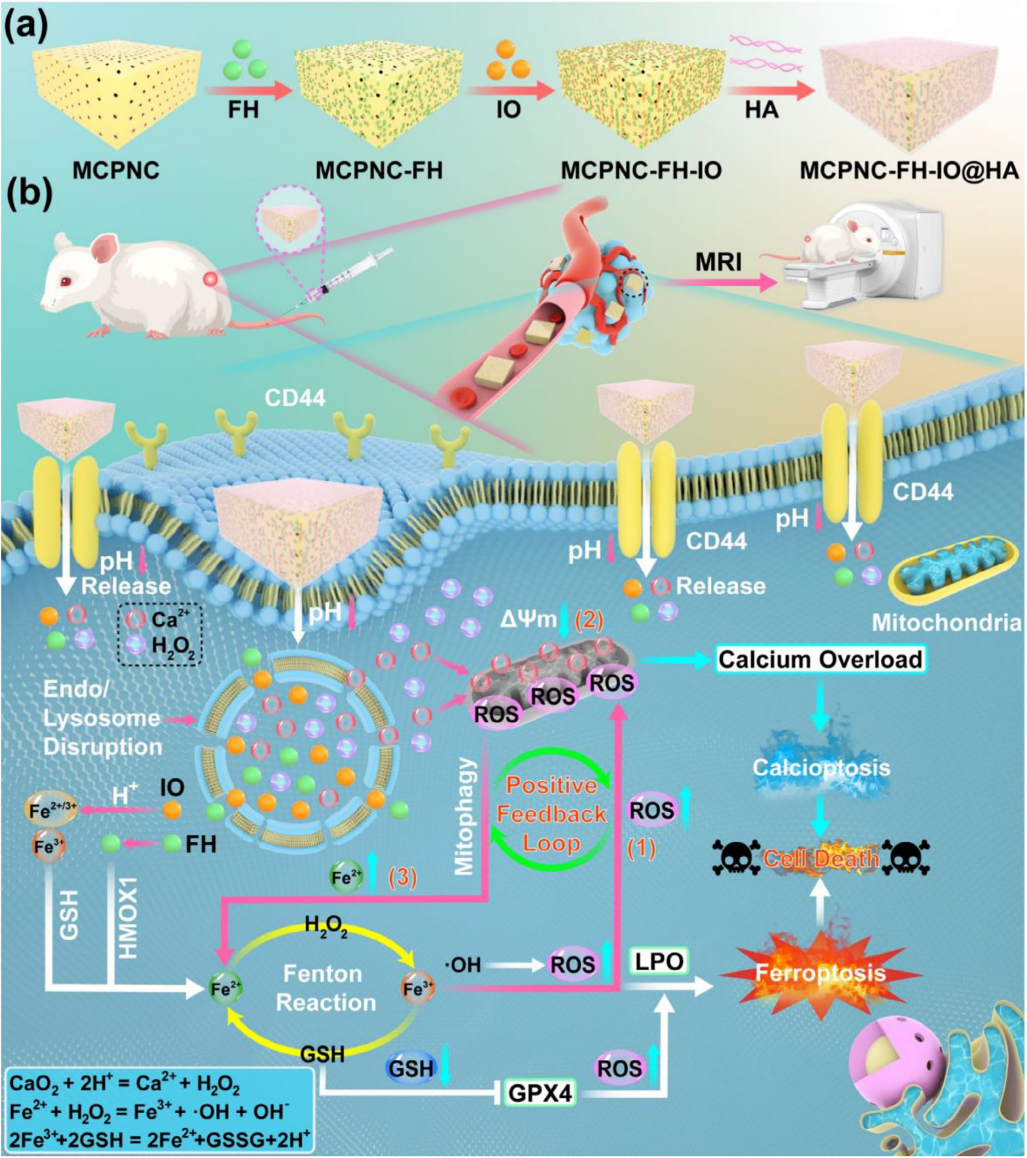
Schematic illustration for the synthesis of MCPNC‐FH‐IO@HA a), and the mechanism (b) of high efficacy tumor therapy by promoting positive feedback loop of Fenton reaction using the MCPNC‐FH‐IO@HA.

To overcome the above‐mentioned problems, based on our newly developed mesoporous CP nanocuboid (MCPNC), a nanoplatform was designed and constructed to augment the capabilities of NCs in drug delivery across biological barriers and improve their tumor accumulation for enhanced cancer therapy. Typically, the MCPNC was synthesized using PVP as the capping agent via a hydrolysis precipitation method. As illustrated in **Scheme** **1**a, MCPNC was employed to sequentially load ferroheme (FH) and IO with *T*_1_ MRI performance (<5.0 nm), yielding MCPNC‐FH‐IO. Furthermore, hyaluronic acid (HA) was modified to the surface of MCPNC‐FH‐IO to enhance its active targetability toward tumor cells through the specific interaction between the CD44 receptor and HA ligand. The HA conjugation can also reduce the risk of premature drug leakage, thereby enhancing tumor therapeutic efficacy. After intravenous injection into the body and accumulation in tumors (Scheme 1b), MCPNC‐FH‐IO@HA can be degraded due to the acidity of the tumor microenvironment (TME) and late endosomes, releasing Fe^2+/3+^, FH, Ca^2+^, and H_2_O_2_. The FH can be degraded by heme oxygenase‐1 (HMOX1),^[^ ^27^, ^28^, ^29^ ^]^ which is highly expressed in tumor cells, leading to the release of Fe^2+^. These released components synergistically promote the positive feedback loop of the Fenton reaction through reactive oxygen species (ROS) generation and mitophagy, thereby realizing enhanced therapeutic efficacy against tumors. The specific mechanism of the above‐mentioned positive feedback loop of the Fenton reaction is given below: 1) The generation of ROS through the participation of Fe^2^⁺ in the Fenton reaction can disrupt mitochondrial membranes and synergistically accelerate the unbalance of mitochondrial homeostasis with Ca^2+^.^[^ ^30^, ^31^, ^32^, ^33^ ^]^ 2) The loss of mitochondrial membrane potential subsequently activates mitochondrial autophagy signaling in tumor cells. After mitophagy, intracellular endogenous Fe ions stored in the mitochondria are released.^[^ ^34^, ^35^, ^36^ ^]^ 3) The released Fe ions can further participate in the Fenton reaction, and lead to the production of a significant amount of ROS, which thereby establishes the positive feedback loop of the Fenton reaction. These aforementioned processes promote the occurrence of ferroptosis and calciptosis in tumor cells, which ultimately result in cell death. Both in vitro and in vivo experimental results demonstrate that our MCPNC‐FH‐IO@HA can be effectively uptaken by tumor cells and inhibit the growth and development of tumors.

## 2 Results and Discussion

### 2.1 Synthesis and Characterization of MCPNC

**Figure 1:**
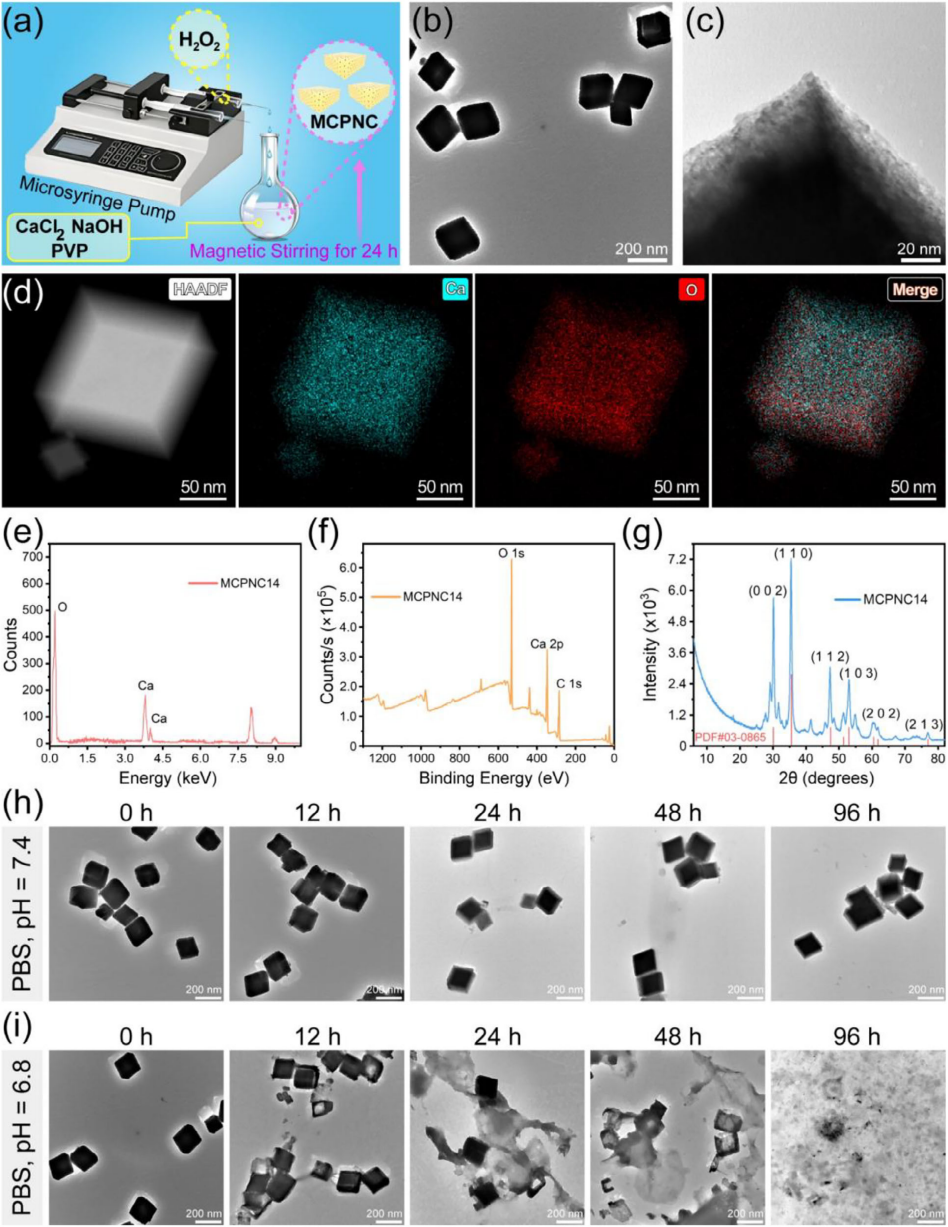
a) Schematic illustration for the synthesis of MCPNC. b,c) TEM image of MCPNC14 (b), and its surface showing the mesoporous structure (c). d) HAADF‐STEM elemental mapping of MCPNC14. e–g) Energy dispersive X‐ray (EDX) spectrum (e), X‐ray photoelectron spectroscopy (XPS) spectrum (f), and X‐ray diffraction (XRD) pattern (g) of MCPNC14. h,i): TEM images of MCPNC14 after incubation in PBS at pH 7.4 h), or pH 6.8 i) for 0, 12, 24, 48, or 96 h.

The conditions of the hydrolysis precipitation reaction (**Figure** **1**a) were optimized to obtain MCPNC. PVP was used as a capping agent for MCPNC preparation, which enables MCPNC to form a cubic shape.^[^ ^37^, ^38^, ^39^ ^]^ The influence of PVP, CaCl_2_, NaOH, H_2_O_2_, and ethanol concentrations on the morphology and average particle sizes of MCPNC1‐17 (Table S1, Supporting Information) was assessed. Figures S1–S10 (Supporting Information) and Figure 1b show the morphology and the average particle sizes of MCPNC2‐17 measured by transmission electron microscopy (TEM). 2000–250.0 µ _m_ of PVP solutions were used to synthesize MCPNC1‐5 (Figure S1, Supporting Information). The 2000 µm of PVP solution can easily cause severe aggregations of MCPNC1, forming precipitation. 500 µm was chosen as the optimal PVP concentration for MCPNC synthesis because the average particle size of MCPNC4 measured by TEM is the smallest among MCPNC2‐5 (Figure S2, Supporting Information).

Furthermore, MCPNC4 and MCPNC6‐9 were synthesized using CaCl_2_ solutions with concentrations ranging from 400 to 25.0 mg mL^−1^, respectively. As shown in Figure S3 (Supporting Information), MCPNC8 failed to form a cubic structure due to insufficient concentration of the CaCl_2_ solution. No product was obtained for MCPNC9 because the concentration of the CaCl_2_ solution is too low. Among MCPNC4, MCPNC6, and MCPNC7, MCPNC7 exhibits the smallest average particle size (Figures S2 and S4, Supporting Information). Therefore, 100 mg mL^−1^ was considered the optimal concentration of CaCl_2_ solution for MCPNC synthesis.

Moreover, 0.5–4.0 m NaOH solutions were used for the synthesis of MCPNC7 and MCPNC10‐12. As shown in Figure S5 (Supporting Information), no nanocuboid was found for MCPNC12 because 4.0 m of NaOH solution was too high. The average particle sizes of MCPNC7 and MCPNC11 are larger than that of MCPNC10, whose NaOH concentration (1.0 m) should be optimal (Figures S4 and S6, Supporting Information).

The influence of H_2_O_2_ concentration (30–5.0%) on the particle size of MCPNC10 and MCPNC13‐15 was further evaluated. It can be observed from Figure 1b and Figures S6–S8 (Supporting Information) that MCPNC14 (146.9 nm) is significantly smaller than MCPNC10 (237.4 nm), MCPNC13 (195.6 nm), and MCPNC15 (296.3 nm). Therefore, the optimal concentration of H_2_O_2_ for MCPNC preparation is 10%.

Finally, the influence of ethanol concentration (99.7–50%) on the particle size of MCPNC was investigated. Figures S9 and S10 (Supporting Information) show that the particle sizes of MCPNC16 and 17 become larger as the concentration of ethanol decreases. Therefore, 99.7% was considered as the optimal ethanol concentration, and the final optimal sample is MCPNC14 with an average diameter of 146.9 nm.

Based on Figure 1b, the morphology of MCPNC14 was observed by further tenfold magnification (Figure 1c). It can be clearly observed that MCPNC14 has a mesoporous structure. Furthermore, the Brunauer‐Emmett‐Teller (BET) analysis indicates that MCPNC14 exhibits a specific surface area of 108.1 m^2^ g^−1^ (Figure S11a, Supporting Information) and an average pore size of 5.9 nm (Figure S11b, Supporting Information), which further verifies that MCPNC14 has a mesoporous structure that is conductive to drug loading.

High‐angle annular dark‐field scanning TEM (HAADF‐STEM)‐based elemental mapping was used to analyze the elemental composition of MCPNC14. As shown in Figure 1d, MCPNC14 contains Ca (cyan) and O (red). Moreover, the energy dispersive X‐ray spectroscopy (EDX) further demonstrates the presence of Ca and O elements in the MCPNC14 (Figure 1e). Notably, the peak at 3.7 keV corresponds to the Kα peak of Ca, which represents the most characteristic peak in EDX analysis of Ca.^[^ ^40^, ^41^, ^42^ ^]^ Meanwhile, the peak at 4.0 keV is attributed to the Kβ peak of Ca, which serves as a supplementary characteristic peak for confirming the presence of Ca. The X‐ray photoelectron spectroscopy (XPS) of MCPNC14 shows the levels of Ca2p and O1s, demonstrating that the component of MCPNC14 is CaO_2_ (Figure 1f).^[^ ^43^, ^44^, ^45^ ^]^ The X‐ray diffraction patterns (XRD) of MCPNC14 (Figure 1g) reveal characteristic diffraction peaks at (000), (110), (112), (103), (202), and (213) crystallographic orientations, which match well with those from references for calcium peroxide.^[^ ^46^, ^47^, ^48^ ^]^ The above‐mentioned results indicate the successful preparation of MCPNC14.

Figure 1h,i shows the morphological changes of MCPNC14 after incubation in phosphate‐buffered saline (PBS) at pH 7.4 or pH 6.8, respectively. The structure of MCPNC14 remains basically unchanged after incubation in PBS at pH 7.4 for 96 h. However, the construction of MCPNC14 begins to be broken after incubation with PBS at pH 6.8 for 12 h, and is almost completely degraded after incubation for 96 h. The degradation of MCPNC14 triggered by weakly acidic conditions is related to its main component CaO_2_. Specifically, CaO_2_ can react with H^+^ in the acidic TME, which results in the release of Ca^2+^ and H_2_O_2_. This lays the foundation for effective degradation and release behavior of MCPNC14 in vivo for tumor therapy.

### 2.2 Synthesis and Characterization of MCPNC‐FH‐IO@HA

The loading content and loading efficiency of FH in MCPNC14‐FH1‐5 were quantified via inductively coupled plasma optical emission spectrometry (ICP‐OES) and calculated using the Equations (1) and (2), which are presented in Table S2 (Supporting Information).

(1) $$\begin{array}{ccc} & & \mathrm{FH}\;\mathrm{Loading}\;\mathrm{Content}\;\left(\%\right)=\\ & & \mathrm{Mass}\;\mathrm{of}\;\mathrm{Loaded}\;\mathrm{FH}/\mathrm{Mass}\;\mathrm{of}\;\mathrm{MCPNC}14\times 100\% \end{array}$$

(2) $$\begin{array}{ccc} & & \mathrm{FH}\;\mathrm{Loading}\;\mathrm{Efficiency}\;\left(\%\right)=\\ & & \mathrm{Mass}\;\mathrm{of}\;\mathrm{Loaded}\;\mathrm{FH}/\mathrm{Mass}\;\mathrm{of}\;\mathrm{Feeding}\;\mathrm{FH}\times 100\% \end{array}$$

MCPNC14‐FH2 was selected as the optimal sample for subsequent experiments due to the most suitable loading efficiency (60.6%) and loading content (22.3%) of FH among MCPNC14‐FH1‐5.

Furthermore, IO was loaded onto the surface of MCPNC14‐FH2 to synthesize MCPNC14‐FH2‐IO1‐5, achieving MRI visualization for drug delivery. The IO was synthesized via a co‐precipitation method^[^ ^35^, ^49^, ^50^ ^]^ and observed by TEM. As shown in Figure S12a,b (Supporting Information), IO exhibits excellent dispersibility and well‐defined spherical morphology with uniform size (≈3.68 nm). The loading content and loading efficiency of IO on MCPNC14‐FH2‐IO1‐5 were measured by ICP‐OES and calculated using Equations (3) and (4), which are presented in Table S3 (Supporting Information).

(3) $$\begin{array}{ccc} & & \mathrm{IO}\;\mathrm{Loading}\;\mathrm{Content}\;\left(\%\right)=\\ & & \mathrm{Mass}\;\mathrm{of}\;\mathrm{Loaded}\;\mathrm{IO}/\mathrm{Mass}\;\mathrm{of}\;\mathrm{MCPNC}14\times 100\% \end{array}$$

(4) $$\begin{array}{ccc} & & \mathrm{IO}\;\mathrm{Loading}\;\mathrm{Efficiency}\;\left(\%\right)=\\ & & \mathrm{Mass}\;\mathrm{of}\;\mathrm{Loaded}\;\mathrm{IO}\;/\;\mathrm{Mass}\;\mathrm{of}\;\mathrm{Feeding}\;\mathrm{IO}\times 100\% \end{array}$$

MCPNC14‐FH2‐IO3 was identified as the optimal sample for subsequent experiments due to the ideal drug loading efficiency (66.3%) and loading content (17.3%) among MCPNC14‐FH2‐IO1‐5.

To further enhance the active targeting capability of the nanoplatform, minimize its toxicity and side effects on normal tissues and organs, and improve the therapeutic efficacy of tumors, HA was modified on the surface of MCPNC14‐FH2‐IO3. MCPNC14‐FH2‐IO3@HA1‐6 were prepared with HA/Ca feed mass ratios ranging from 1.0 to 0.1 in pure water, and the synthetic conditions along with the characterization results are summarized in Table S4 (Supporting Information). Figures S13 and S14 (Supporting Information) show that MCPNC14‐FH2‐IO3@HA4 exhibits a significantly brighter *T*_1_‐weighted MRI signal and a sufficiently dark *T*_2_‐weighted MRI signal compared to MCPNC14‐FH2‐IO3@HA1‐3, 5, and 6, rendering it more suitable for *T*_1_/*T*_2_ dual‐mode MRI. Figure S15 (Supporting Information) shows the corresponding quantitative results of *r*_1_ and *r*_2_ values for MCPNC14‐FH2‐IO3@HA1‐6. MCPNC14‐FH2‐IO3@HA4 was identified as the optimal sample for subsequent characterization and experiments based on its highest *r*_1_ value (10.3 ± 0.7 mM⁻¹ s⁻¹) and a suitable *r*_2_/*r*_1_ ratio (11.9 ± 1.3) (Table S4, Supporting Information).

Figure S16a,b (Supporting Information) presents the structures of PVP, HA, MCPNC14, and MCPNC14‐FH2‐IO3@HA4 and the relevant Fourier‐transform infrared (FT‐IR) spectra. PVP has a characteristic peak at 1655 cm^−1^ (C═O stretching vibration). The characteristic peak 1652 cm^−1^ of MCPNC14 demonstrates the successful introduction of stabilizer PVP. Furthermore, HA has characteristic peaks at 3430 cm^−1^ (O─H stretching vibration in hydroxyl groups) and 1042 cm^−1^ (C─O stretching vibration in carboxyl groups). The simultaneous existence of the characteristic peaks at 1655, 3430, and 1042 cm^−1^ indicates the successful modification of PVP and HA onto MCPNC14‐FH2‐IO3@HA4.

**Figure 2:**
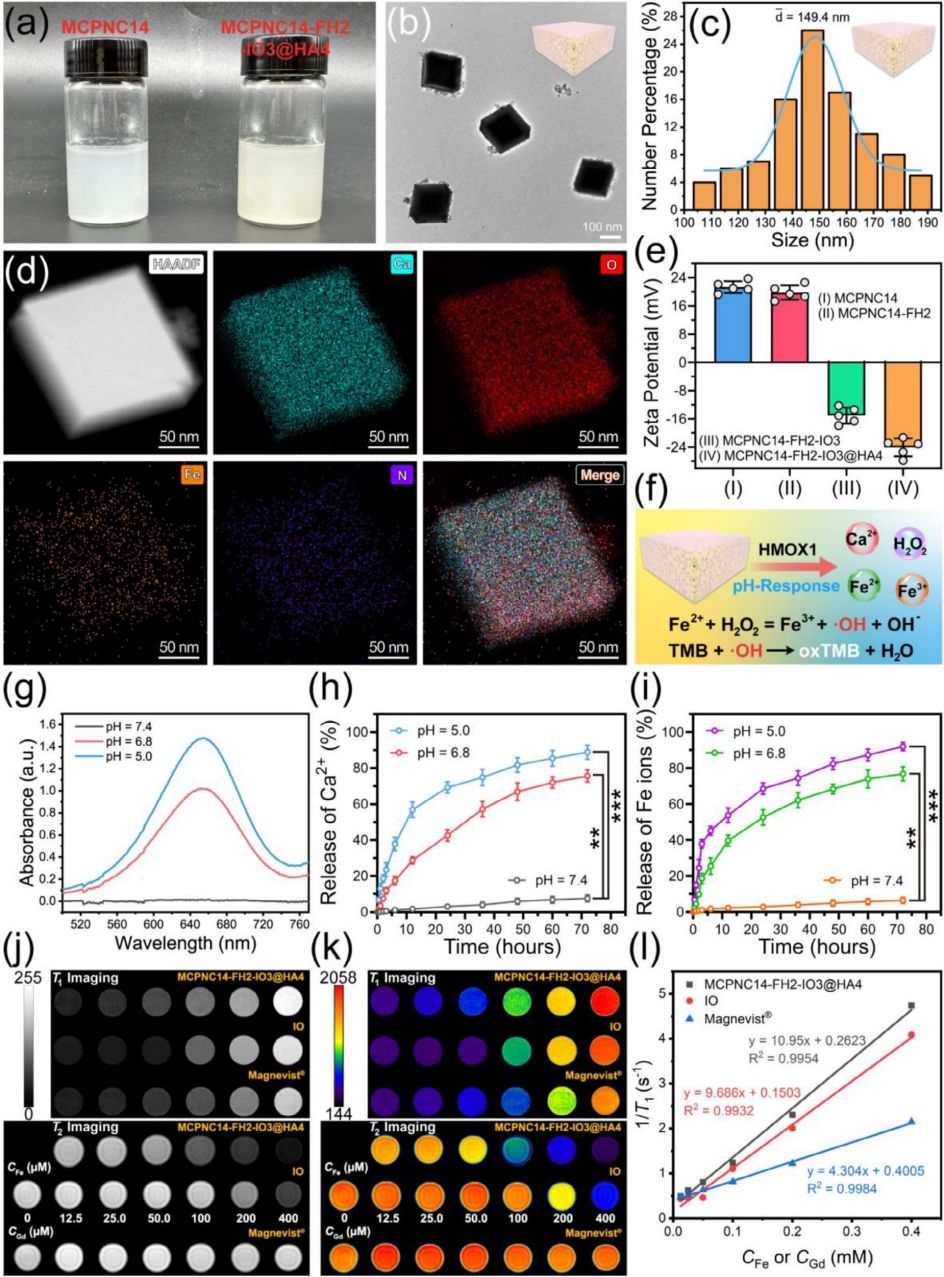
a) The physical photos of MCPNC14 and MCPNC14‐FH2‐IO3@HA4. b–d) TEM image (b), particle size distribution (c), and HAADF‐STEM elemental mapping (d) of MCPNC14‐FH2‐IO3@HA4. e) Zeta potentials of MCPNC14, MCPNC14‐FH2, MCPNC14‐FH2‐IO3 or MCPNC14‐FH2‐IO3@HA4. Mean ± S.D., *n* = 5. f) Schematic illustration for pH responsive degradation of MCPNC14‐FH2‐IO3@HA4 and the mechanism of TMB oxidation. g) UV–vis spectra of TMB solutions incubated with MCPNC14‐FH2‐IO3@HA4 in PBS at pH 5.0, 6.8, or 7.4. h,i) Release behaviors of Ca (h), or Fe (i) ions from MCPNC14‐FH2‐IO3@HA4 in PBS at pH 5.0, 6.8 or 7.4. Mean ± S.D., *n* = 3. j,k) *T*_1_‐ and *T*_2_‐weighted MR images (j), and the corresponding pseudo‐color images (k) of MCPNC14‐FH2‐IO3@HA4, IO, or commercial Magnevist solutions with different concentration of Fe or Gd. l) *T*_1_ relaxation rate (1/*T*_1_) plotted as a function of Fe or Gd concentration for MCPNC14‐FH2‐IO3@HA4, IO, or commercial Magnevist solutions. For *T*_1_ relaxation rates: TE = 8.4 ms, TR = 500 ms. Magnetic field = 3.0 T. ^**^ *p* < 0.01, ^***^ *p* < 0.001.

MCPNC14 and MCPNC14‐FH2‐IO3@HA4 exhibit exceptional dispersion in aqueous solution (**Figure** **2**a). MCPNC14‐FH2‐IO3@HA4 has a uniform particle size, as evidenced by the TEM image (Figure 2b). The actual particle size distribution of 100 nanoparticles measured from TEM images (Figure 2c) confirms the uniformity of MCPNC14‐FH2‐IO3@HA4 within the range of 130–160 nm. As shown in Figure S17 (Supporting Information), the hydrodynamic diameters of MCPNC14 and MCPNC14‐FH2‐IO3@HA4, determined by dynamic light scattering (DLS) are 183.2 and 191.6 nm, respectively. Figure 2d presents the elemental mapping of MCPNC14‐FH2‐IO3@HA4 obtained using HAADF‐STEM. The elements of Ca (cyan), Fe (orange), N (purple), and O (red) can be observed, which indicates the successful preparation of MCPNC14‐FH2‐IO3@HA4. The high surface charge of the NCs results in strong electrostatic repulsion between the NCs, which endows them with exceptional stability and aqueous dispersibility. As presented in Figure 2e, the zeta potential of MCPNC14‐FH2 (19.8 ± 1.8 mV) exhibits no significant difference with that of MCPNC14 (21.4 ± 1.5 mV), indicating that the charge is barely affected by the electroneutral FH modification. Additionally, the loading of IO and the modification with HA result in an increased electronegativity, which places MCPNC14‐FH2‐IO3@HA4 in a state of high negative charge (−24.1 ± 2.3 mV). The high negative charge state can effectively prevent nonspecific uptake of NCs by most normal cells (negatively charged).

Figure 2f depicts a schematic illustration of the process by which MCPNC14‐FH2‐IO3@HA4 undergoes degradation to release Fe^2^⁺, Fe^3^⁺, Ca^2^⁺, and H₂O₂ under acidic conditions in the presence of HMOX1 enzyme, and then ·OH is generated through the Fenton reaction, leading to the oxidation of 3,3′,5,5′‐tetramethylbenzidine (TMB). Generally speaking, the extracellular pH of various tumor types ranges from 6.7 to 7.1,^[^ ^51^ ^]^ and endo/lysosomes maintain an acidic pH of 4.5–6.0.^[^ ^52^, ^53^, ^54^ ^]^ Therefore, pH 6.8 and 5.0 are respectively used to simulate the acidity of TME and endo/lysosomes. Figure 2g illustrates that the UV–vis absorbance of TMB/oxTMB increases as the pH decreases from 7.4 to 5.0, which indicates that the production of ·OH and the rate of the Fenton reaction are pH‐dependent. Furthermore, it can be found from Figure S18 (Supporting Information) that the UV–vis absorption peak values of TMB at 652 nm after incubation at pH 6.8 and 5.0 are significantly different from that at pH 7.4 (^***^ *p* < 0.001). As shown in Figure 2h, the release of Ca^2^⁺ remains below 10% after incubation at pH 7.4 for 72 h, but significantly increases to 42.6 ± 2.6% and 69.3 ± 2.5% respectively, after incubation at pH 6.8 and 5.0 for 24 h. The release behavior of Fe ions (Figure 2i) exhibits a similar pattern, with the lowest release quantity observed after incubation at pH 7.4 for 72 h (6.5 ± 1.4%), and the highest release quantity noted after incubation at pH 5.0 for 72 h (91.9 ± 1.9%). Although the release of Ca and Fe ions from MCPNC14‐FH2‐IO3@HA4 is fast in acidic TME, it is extremely low in neutral environments, which are insufficient to exert toxicity on normal tissues.

In terms of MRI performance, it can be observed from Figure 2j,k that MCPNC14‐FH2‐IO3@HA4 exhibits superior bright and dark signal intensities compared to IO and Magnevist. As presented in Figure 2l and Figure S19 (Supporting Information), based on the corresponding quantitative calculations, the *r*_1_ values and *r*_2_ values of MCPNC14‐FH2‐IO3@HA4 (10.95 and 121.3 mM⁻¹ s⁻¹) are both much higher than those of IO (9.686 and 43.32 mM⁻¹ s⁻¹) and Magnevist (4.304 and 3.995 mM⁻¹ s⁻¹). Additionally, the *r*_2_/*r*_1_ ratio of 11.08 for MCPNC14‐FH2‐IO3@HA4 is well‐suited for *T*_1_/*T*_2_ dual‐mode MRI, which facilitates more advantages for disease diagnosis.

### 2.3 Interaction Between MCPNC14‐FH2‐IO3@HA4 and Cells

**Figure 3:**
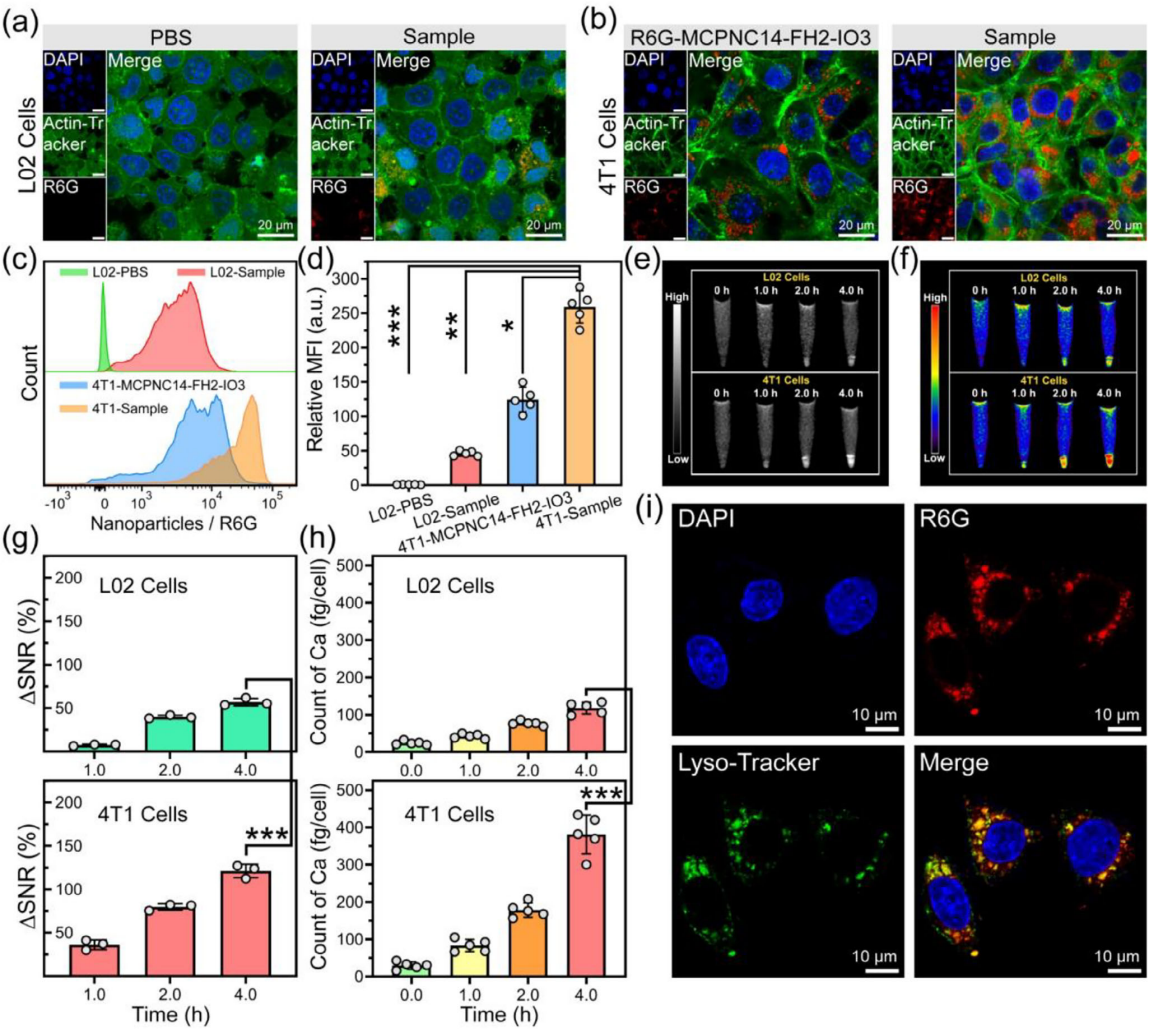
a,b) LSCM photos of L02 cells (a), or 4T1 cells (b) after incubation with PBS, R6G‐MCPNC14‐FH2‐IO3 or the sample (R6G‐MCPNC14‐FH2‐IO3@HA4), showing cellular uptake of the nanoparticles. Green fluorescence: Actin‐Tracker for cytoskeleton; red fluorescence: R6G for nanoparticles; blue fluorescence: DAPI for nuclei. c,d) R6G fluorescence distributions of L02 or 4T1 cells incubated with PBS, R6G‐MCPNC14‐FH2‐IO3 or the sample (R6G‐MCPNC14‐FH2‐IO3@HA4) (c), and the corresponding quantitative analysis (d) determined by flow cytometry. Mean ± S.D., *n* = 5. e,f) *T*_1_‐weighted MR images (e), and the corresponding pseudo‐color images (f) of L02 or 4T1 cells treated with MCPNC14‐FH2‐IO3@HA4 observed on a 3.0 T MRI scanner. g) Quantitative measurement of the *T*_1_ MR images for L02 or 4T1 cells treated with MCPNC14‐FH2‐IO3@HA4. Mean ± S.D., *n* = 3. h) Cellular uptaken amounts of L02 or 4T1 cells after incubation with MCPNC14‐FH2‐IO3@HA4 for 0, 1.0, 2.0, and 4.0 h measured by ICP. Mean ± S.D., *n* = 5. i) LSCM photos of 4T1 cells after incubation with R6G‐MCPNC14‐FH2‐IO3@HA4, showing lysosome escape of the nanoparticles. Green fluorescence: Lyso‐Tracker for lysosomes; red fluorescence: R6G for nanoparticles; blue fluorescence: DAPI for nuclei. ^*^ *p* < 0.05, ^**^ *p* < 0.01, ^***^ *p* < 0.001.

Laser scanning confocal microscopy (LSCM), flow cytometric analysis, and MRI are used to evaluate the cellular uptake behavior of MCPNC14‐FH2‐IO3@HA4 on L02 or 4T1 cells. **Figure** **3**a,b shows that 4T1 cells with treatment of R6G‐loaded MCPNC14‐FH2‐IO3@HA4 (R6G‐MCPNC14‐FH2‐IO3@HA4) exhibit a more pronounced red fluorescence signal than the PBS‐treated or R6G‐MCPNC14‐FH2‐IO3@HA4‐treated L02 cells and R6G‐MCPNC14‐FH2‐IO3‐treated 4T1 cells, which indicates that MCPNC14‐FH2‐IO3@HA4 can be efficiently taken up by tumor cells. Figure 3c presents flow cytometric analysis for the fluorescence intensity of PBS‐treated, R6G‐MCPNC14‐FH2‐IO3‐treated or R6G‐MCPNC14‐FH2‐IO3@HA4‐treated L02 or 4T1 cells, and the relevant quantification of mean fluorescence intensity is shown in Figure 3d. Compared to other treatment groups, 4T1 cells with treatment of R6G‐MCPNC14‐FH2‐IO3@HA4 exhibit statistically significant differences in fluorescence intensity (^*^ *p* < 0.05, ^**^ *p* < 0.01, ^***^ *p* < 0.001), which demonstrates the effective endocytosis of MCPNC14‐FH2‐IO3@HA4. It is worth noting that the uptake of MCPNC14‐FH2‐IO3@HA4 by 4T1 cells is significantly higher than that of MCPNC14‐FH2‐IO3 (^*^ *p* < 0.05). This phenomenon can be attributed to the overexpression of CD44 receptors on the surface of tumor cells,^[^ ^55^, ^56^, ^57^ ^]^ which favors HA binding and thereby facilitates cellular uptake. Therefore, NCs with HA can be more efficiently endocytosed by tumor cells.

Figure 3e,f depicts the *T*_1_‐weighted MR images of L02 and 4T1 cells post‐treatment with MCPNC14‐FH2‐IO3@HA4, acquired using a clinical 3.0 T MRI scanner. The endocytosis of MCPNC14‐FH2‐IO3@HA4 exhibits a time dependence with the highest intensity observed at 4.0 h. The corresponding ΔSNR values, as shown in Figure 3g, further corroborate the aforementioned observations, demonstrating significantly enhanced uptake of MCPNC14‐FH2‐IO3@HA4 by 4T1 cells compared to L02 cells (^***^ *p* < 0.001). Consistent conclusions can also be drawn from the ICP measurements of intracellular Ca^2+^ levels (Figure 3h).

Additionally, after incubation for 4.0 h, the distribution of R6G‐MCPNC14‐FH2‐IO3@HA4 (red fluorescence) and Lyso‐Tracker‐stained lysosomes of 4T1 cells (green fluorescence) were visualized using LSCM. Figure 3i reveals apparent spatial separation between red and green fluorescence, which indicates the successful lysosomal escape. This property facilitates enhanced intracellular delivery of FH and IO, which supports a groundwork for subsequent tumor theranostics.

### 2.4 in vitro Verification of Cell Death

**Figure 4:**
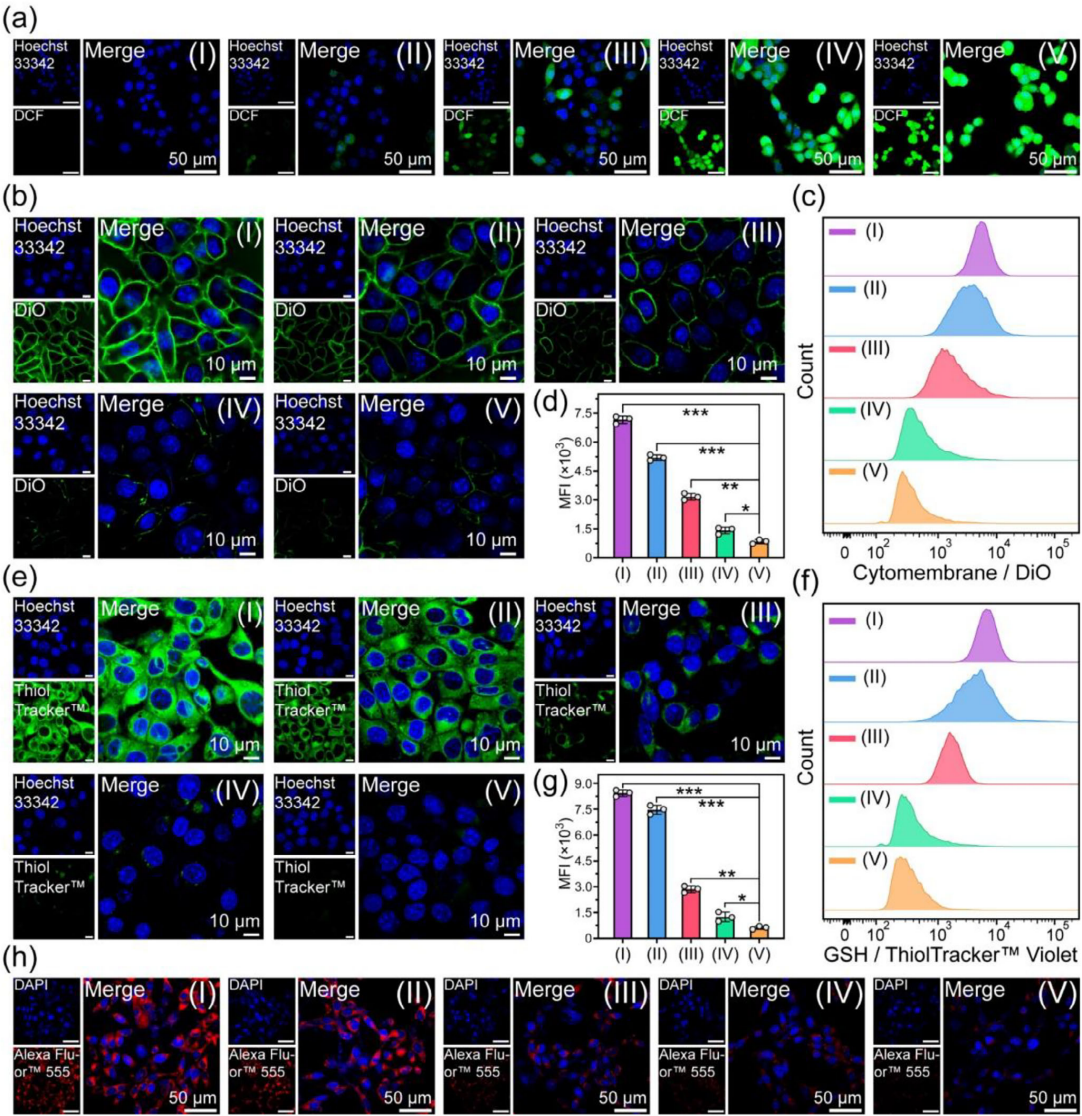
a) LSCM photos of DCFH‐DA‐stained 4T1 cells with treatments of PBS (group I), MCPNC14 (group II), MCPNC14‐FH2 (group III), MCPNC14‐FH2‐IO3 (group IV), or MCPNC14‐FH2‐IO3@HA4 (group V), showing ROS generation. The nuclei stained with Hoechst 3342 are blue. b) LSCM images of 4T1 cells after treatments with groups I–V, and staining with DiO probe showing the situation of cell membrane. The decreased green fluorescence indicates cell membrane damage. c,d) DiO fluorescence distributions (c), and the corresponding quantitative analysis (d) of 4T1 cells after different treatments (groups I–V) measured by flow cytometry. e) LSCM photos of 4T1 cells after incubation with groups I–V, and staining with ThiolTracker Violet showing GSH levels. f,g) ThiolTracker Violet fluorescence distributions (f), and the corresponding quantitative analysis (g) of 4T1 cells after the above‐mentioned treatments (groups I–V) determined by flow cytometry for the intracellular GSH levels assay. h) LSCM images of 4T1 cells stained with Alexa Fluor 555‐Labeled Donkey Anti‐Rabbit IgG (H + L), showing intracellular GPX4 levels. The nuclei stained with DAPI are blue. Mean ± S.D., *n* = 3. ^*^ *p* < 0.05, ^**^ *p* < 0.01, ^***^ *p* < 0.001.

The LSCM pictures of 4T1 cells with treatment of PBS (group I), MCPNC14 (group II), MCPNC14‐FH2 (group III), MCPNC14‐FH2‐IO3 (group IV), or MCPNC14‐FH2‐IO3@HA4 (group V) are displayed in **Figure** **4**a. The green fluorescence emitted by the DCFH‐DA probe demonstrates the ROS generation. Compared to groups I‐III, groups IV and V exhibit substantial generation of ROS, which demonstrates the robust ROS‐generating capacity of MCPNC14‐FH2‐IO3 and MCPNC14‐FH2‐IO3@HA4, attributed to the enhanced Fenton reaction caused by the increased release of Fe ions from IO degradation. Meanwhile, most of the 4T1 cells in groups IV and V appear round without normal cell morphology after DCFH‐DA staining.

Figure S20a,b (Supporting Information) presents DCF fluorescence distribution with the aforementioned treatment of groups I–V, and the flow cytometric quantifications of ROS generation in 4T1 cells. The results indicate that the fluorescence intensity in group V is significantly higher than that in groups I–IV (^*^ *p* < 0.05, ^**^ *p* < 0.01, ^***^ *p* < 0.001), which reinforces the potent ROS‐generating capability of MCPNC14‐FH2‐IO3@HA4.

Figure S21 (Supporting Information) shows the LSCM photos of 4T1 cells (stained with JC‐1 probe) with treatment of groups I‐V. The red fluorescence indicates the aggregation of JC‐1 in healthy mitochondria with normal mitochondrial membrane potential, whereas the green fluorescence represents the monomeric form of JC‐1 in cytoplasm due to the decreased mitochondrial membrane potential. It can be seen that the intensity of green fluorescence of group IV and V is much stronger than that of groups I–III because the increased concentration of Fe ions in groups IV and V accelerates the consumption of H_2_O_2_, which enhances the degradation of CaO_2_, and thereby increases the concentration of Ca^2^⁺. Upon an abnormal increase of Ca^2^⁺ concentration in cytoplasm, mitochondria actively take up excess Ca^2^⁺ through the mitochondrial calcium uniporter (MCU), exceeding their buffering capacity,^[^ ^58^, ^59^, ^60^ ^]^ which results in mitochondrial dysfunction and a subsequent decrease in mitochondrial membrane potential. Furthermore, the co‐localization of autophagy marker microtubule‐associated protein 1 light chain 3 (LC3) with mitochondria was observed by LSCM to verify whether the mitophagy pathway was activated.^[^ ^61^ ^]^ As presented in Figure S22 (Supporting Information), the red fluorescence (mitochondria) and the green fluorescence (LC3) are obviously separated in group I, which indicates that almost no mitophagy occurs in PBS‐treated 4T1 cells. However, obvious yellow fluorescence composed of red fluorescence superimposed with green fluorescence appears in groups II–V, which proves that mitophagy is activated.

As presented in Figure S23 (Supporting Information), the red fluorescence (the reduced state of C11‐BODIPY^581/591^) in the 4T1 cells with treatment of group IV and group V transitions to green (the oxidized state of C11‐BODIPY^581/591^), which demonstrates the massive generation of lipid peroxidation (LPO). Consequently, MCPNC14‐FH2‐IO3 and MCPNC14‐FH2‐IO3@HA4 can trigger LPO in 4T1 cells, which is attributed to abundant ROS production via Fe ion‐mediated Fenton reactions.

Flow cytometric analysis presented in Figure S24a,b (Supporting Information) depicts the distribution of C11‐BODIPY^581/591^ green fluorescence and the quantitative measurements of intracellular LPO production in 4T1 cells with treatment of groups I–V. Notably, 4T1 cells treated with MCPNC14‐FH2‐IO3@HA4 exhibit the strongest BODIPY^581/591^ green fluorescence among the groups I–V (^*^ *p* < 0.05, ^***^ *p* < 0.001).

Figure 4b presents LSCM photos of 4T1 cells subjected to various treatments and stained with the 3,3′‐dioctadecyloxacarbocyanine (DiO) probe. It can be seen that the decrease in green fluorescence indicates cellular membrane damage. Notably, the membrane structures of 4T1 cells with treatment of MCPNC14‐FH2‐IO3 and MCPNC14‐FH2‐IO3@HA4 are more markedly impaired due to the generation of LPO compared to that of groups I–III.

Flow cytometric analysis presented in Figure 4c,d depicts the DiO fluorescence distribution and quantitative measurements of 4T1 cells with treatment of groups I–V. Statistically significant differences are observed between groups V and I–IV (^*^ *p* < 0.05, ^**^ *p* < 0.01, ^***^ *p* < 0.001). The cellular membrane damage induced by MCPNC14‐FH2‐IO3@HA4‐generated LPO serves as a marker of ferroptosis.

Figure 4e shows the LSCM photos of 4T1 cells stained with ThiolTracker Violet (a GSH probe). It can be seen that the green fluorescence of groups II–V gradually decreased compared with group I, which suggests the consumption of GSH. The distribution of ThiolTracker Violet green fluorescence and quantitative measurements of intracellular GSH depletion assessed by flow cytometry (Figure 4f,g) demonstrate that 4T1 cells treated with group V exhibit the most reduced GSH levels among groups I–V (^*^ *p* < 0.05, ^**^ *p* < 0.01, ^***^ *p* < 0.001). This phenomenon is primarily attributed to the combined consumption of GSH by Fe^3+^ generated through the Fenton reaction and released from IO degradation (2Fe^3+^ + 2GSH = 2Fe^2+^ + GSSG + 2H^+^).

Figure 4h shows the LSCM photos of 4T1 cells stained with Alexa Fluor 555‐Labeled Donkey Anti‐Rabbit IgG (H + L) and treated with groups I–V. A progressive attenuation of red fluorescence intensity is observed in 4T1 cells from group I to V, which indicates a corresponding gradual reduction in GPX4 levels. That's because GSH serves as the essential substrate for GPX4 to convert cytotoxic LPO to non‐toxic lipid hydroxides (LOH), and its excessive consumption can directly reduce the GPX4 activity.

The quantitative analysis results obtained using a GPX4 assay kit (Figure S25, Supporting Information) reveal that the relative average levels of intracellular GPX4 with treatments of groups I–V are respectively 100 ± 3.6, 89.9 ± 2.8, 58.8 ± 4.4, 27.8 ± 1.5, and 14.6 ± 1.2%, which indicates that MCPNC14‐FH2‐IO3@HA4 has the most potent ability to downregulate GPX4. Downregulated expression of GPX4 makes tumor cells sensitive to ferroptosis, further elucidating the cause of intracellular substantial ROS generation and resultant LPO accumulation.

Figure S26 (Supporting Information) presents the LSCM photos of 4T1 cells with treatment of groups I–V, stained with fluorescence‐labeled H2A histone family member X (γ‐H2AX) to track the levels of intracellular DNA damage (green). Among groups I‐V, 4T1 cells treated with MCPNC14‐FH2‐IO3@HA4 (group V) exhibit the strongest green fluorescence in nuclei. The corresponding fluorescence distributions and quantitative analysis (Figure S27a,b, Supporting Information) measured by flow cytometry indicate that MCPNC14‐FH2‐IO3@HA4 can induce the most significant DNA damage in tumor cells among the groups I–V (^*^ *p* < 0.05, ^**^ *p* < 0.01, ^***^ *p* < 0.001). This phenomenon is attributed to the combined effect of intracellular oxidative stress resulting from mitochondrial damage induced by Ca^2+^ overload and abundant ·OH radicals generated via the Fenton reaction. The aforementioned results on ROS generation, the reduction of mitochondrial membrane potential, LPO production, the disruption of cell membrane, GSH consumption, GPX4 levels, and DNA damage all demonstrate that our MCPNC14‐FH2‐IO3@HA4 can trigger both ferroptosis and calcicoptosis of tumor cells.

### 2.5 in vitro Antitumor Therapy Efficacy

**Figure 5:**
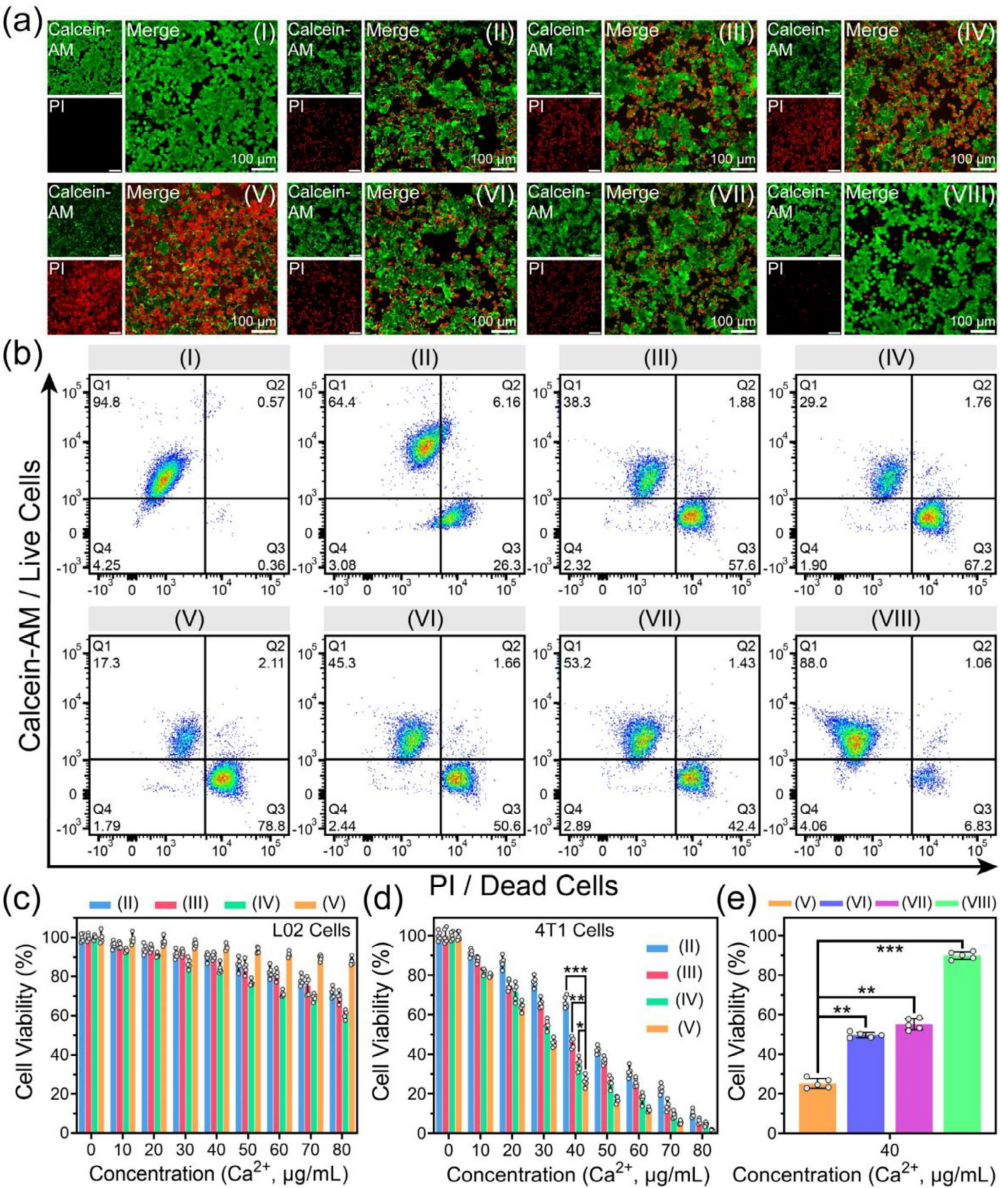
a,b) LSCM images (a), and the corresponding flow cytometry analysis (b) of 4T1 cells after treatments with PBS (group I), MCPNC14 (group II), MCPNC14‐FH2 (group III), MCPNC14‐FH2‐IO3 (group IV), MCPNC14‐FH2‐IO3@HA4 (group V), MCPNC14‐FH2‐IO3@HA4 plus DFO (group VI), MCPNC14‐FH2‐IO3@HA4 plus BAPTA‐AM (group VII), or MCPNC14‐FH2‐IO3@HA4 plus both DFO and BAPTA‐AM (group VIII). Green fluorescence: Calcein‐AM for live cells; red fluorescence: PI for dead cells. c,d) Cell viabilities of L02 cells (c), or 4T1 cells (d) after the corresponding treatments (groups II–V) for 24 h. e) Cell viabilities of 4T1 cells with treatments of groups V–VIII with 40 µg mL^−1^ of Ca^2+^ concentration for 24 h. Mean ± S.D., *n* = 5. ^*^ *p* < 0.05, ^**^ *p* < 0.01, ^***^ *p* < 0.001.

**Figure** **5**a displays the LSCM photos of 4T1 cells (co‐stained with calcein acetoxymethyl ester (Calcein‐AM, green fluorescence for live cells) and propidium iodide (PI, red fluorescence for dead cells)) after various treatments for 24 h. Groups I‐III exhibits few dead cells, demonstrating that ferroptosis occurs infrequently in the absence of Fe ions. A lot of dead cells are observable in group IV and group V because the large amount of Fe ions released by IO degradation and mitophagy will form the positive feedback loop of Fenton reaction, and thereby trigger ferroptosis and calcicoptosis for the 4T1 cells. Compared with groups IV and V, group VI–VIII shows a reduced number of dead cells due to the chelating effects of DFO on Fe ions and BAPTA‐AM on Ca^2+^, which significantly decrease the intracellular levels of Fe and Ca ions, thereby inhibiting the occurrence of ferroptosis and calcicoptosis.

Figure 5b and Figure S28 (Supporting Information) present the quantitative analysis of the corresponding cell viability or mortality rates determined by flow cytometry for 4T1 cells (co‐stained with Calcein‐AM and PI) after diverse treatments. Notably, group V (MCPNC14‐FH2‐IO3@HA4) exhibits a significantly higher dead cell rate than groups I–IV and VI–VIII (^*^ *p* < 0.05, ^**^ *p* < 0.01, ^***^ *p* < 0.001), which aligns with the above‐mentioned LSCM results.

Furthermore, the cell viability of L02 cells (Figure 5c) or 4T1 cells (Figure 5d) after 24 h of treatments with varying concentrations of groups II–V was assessed using the MTT assay. It is worth noting that L02 cells treated with groups II–V maintain high cell viability (90.1 ± 1.9, 90.6 ± 1.6, 84.1 ± 2.2, 95.1 ± 1.3% at a Ca^2+^ concentration of 40 µg mL^−1^ respectively) because L02 cells have sufficient CAT enzymes to prevent oxidative stress caused by excessive H_2_O_2_. For 4T1 cells, MCPNC14‐FH2‐IO3@HA4 treatment (group V) exhibits a cell viability of 26.4 ± 2.5% at a Ca^2+^ concentration of 40 µg mL^−1^, which is significantly lower than that of groups II–IV (^*^ *p* < 0.05, ^**^ *p* < 0.01, ^***^ *p* < 0.001). Additionally, the corresponding blocking experiments conducted at a Ca^2+^ concentration of 40 µg mL^−1^ demonstrate the restoration of cell viability (Figure 5e), aligning with the results of LSCM and flow cytometry. The findings from the LSCM, flow cytometry, and MTT assays collectively indicate that MCPNC14‐FH2‐IO3@HA4 exhibits remarkable therapeutic efficacy against cancer cells to the ferroptosis and calcicoptosis.

### 2.6 in vivo MRI Performance and Biodistribution

**Figure 6:**
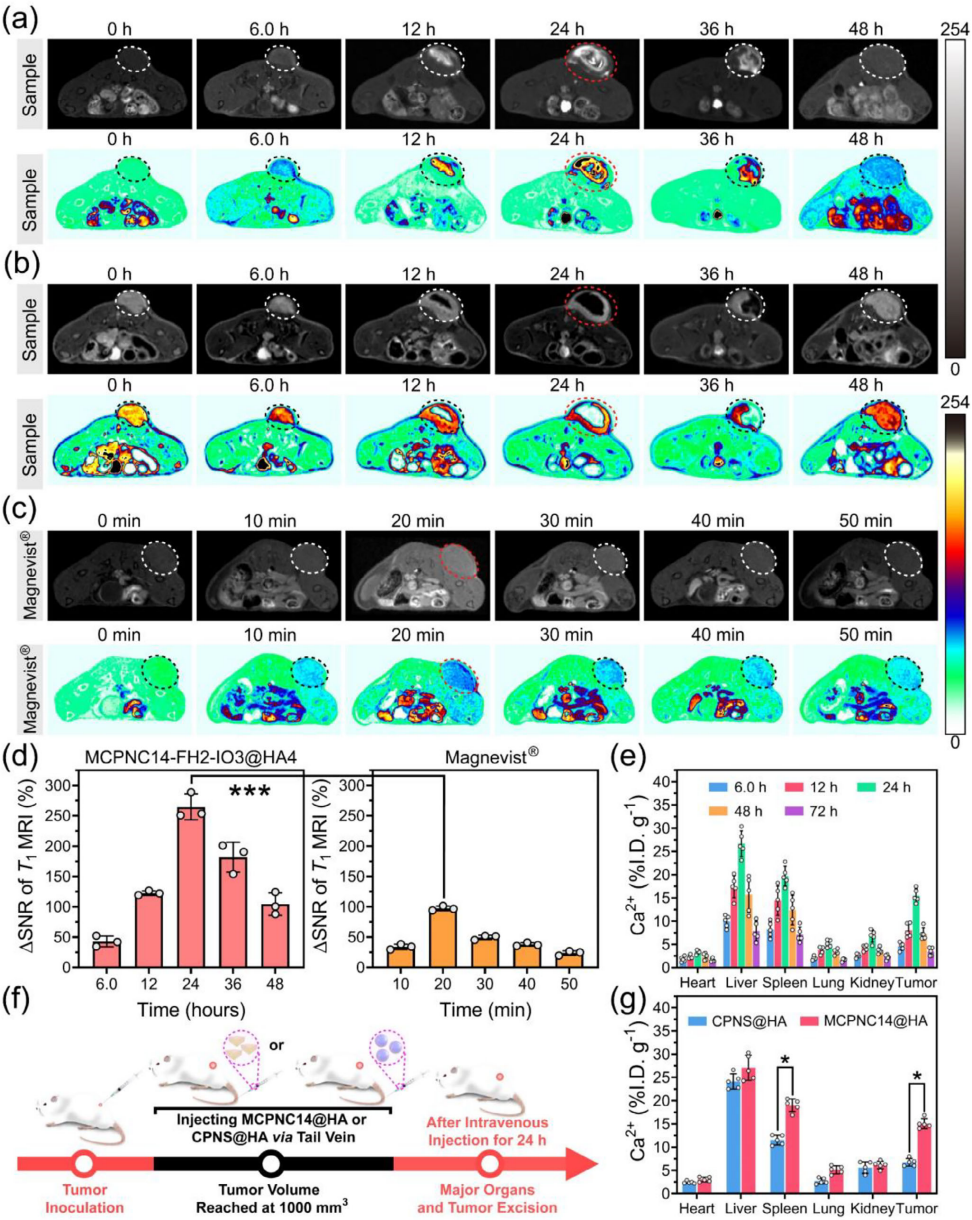
a,b) *T*_1_‐ (a) and *T*_2_‐weigted (b) MR images and the corresponding pseudo‐color images of 4T1 tumor‐bearing mice showing tumors before (0 h) and after intravenous injection of the sample (MCPNC14‐FH2‐IO3@HA4) for 6.0, 12, 24, 36, and 48 h. c) *T*_1_‐weigted MR images and the corresponding pseudo‐color images of 4T1 tumor‐bearing mice showing tumors before (0 min) and after intravenously injecting Magnevist for 10, 20, 30, 40, and 50 min. The magnetic field is 7.0 T. d) Quantitative measurement of the *T*_1_‐weigted MR images for MCPNC14‐FH2‐IO3@HA4 or commercial Magnevist. Mean ± S.D., *n* = 3. e) Biodistribution of Ca^2+^ in normal tissues and tumors of 4T1 tumor‐bearing mice after injection of MCPNC14‐FH2‐IO3@HA4 intravenously. Mean ± S.D., *n* = 5. f) Schematic diagram of the procedure for 4T1 tumor inoculation, and the excision of major organs and tumors excision after intravenously injecting MCPNC14@HA or CPNS@HA for 24 h. g) Biodistribution of Ca^2+^ in normal tissues and tumors of 4T1 tumor‐bearing mice after 24 h intravenous injection of MCPNC14@HA or CPNS@HA. Mean ± S.D., *n* = 5. ^*^ *p* < 0.05, ^***^ *p* < 0.001.

To further investigate the MRI performance of MCPNC14‐FH2‐IO3@HA4 in tumors, its half‐life of blood circulation was first measured and calculated using ICP‐OES to establish the optimal time range of the brightest or darkest signal at tumors, thereby guiding MRI performance evaluation of MCPNC14‐FH2‐IO3@HA4. The blood circulation half‐life of MCPNC14‐FH2‐IO3@HA4 was determined as 10.5 h (Figure S29, Supporting Information), which prompts in vivo 7.0 T MRI evaluation at 6.0–48 h post‐injection. **Figure** **6**a–c displays MR images of 4T1 tumor‐bearing mice after respectively intravenous injection of the sample (MCPNC14‐FH2‐IO3@HA4) or the commercial CAs Magnevist. The tumor site maximal positive (Figure 6a) and negative (Figure 6b) MRI signal (24 h) are significantly improved by MCPNC14‐FH2‐IO3@HA4. Additionally, the positive MRI signal at tumor site reaches maximum intensity at 20 min post‐injection (intravenous) of Magnevist (Figure 6c). Notably, the positive MRI signal at tumor site in MCPNC14‐FH2‐IO3@HA4 group is markedly stronger than that in the Magnevist group.

Furthermore, the tumor signal‐to‐noise ratio (SNR) after injection of CAs was quantified, and the ΔSNR was calculated according to the Equations (5) and (6):

(5) $$\mathrm{SNR}=SI_{\mathrm{mean}}/SD_{\mathrm{noise}}$$

(6) $$\Delta \mathrm{SNR}=\left(\mathrm{SN}R_{\mathrm{post}}-\mathrm{SN}R_{\mathrm{pre}}\right)/\mathrm{SN}R_{\mathrm{pre}}\times 100\%$$

Figure 6d displays that *T*_1_ ΔSNR value of MCPNC14‐FH2‐IO3@HA4 at 24 h (264.5 ± 17.4%) is significantly higher than that of Magnevist at 20 min (97.4 ± 3.1%) (^***^ *p* < 0.001). Figure S30 (Supporting Information) shows that the *T*_2_ ΔSNR value of MCPNC14‐FH2‐IO3@HA4 at 24 h (‐95.5 ± 0.7%) is significantly lower than that at 6.0 h (−16.5 ± 2.4%) or 48 h (−25.8 ± 4.2%) (^***^ *p* < 0.001), which indicates the *T*_2_ imaging capability. These results indicate that MCPNC14‐FH2‐IO3@HA4 exhibits excellent *T*_1_/*T*_2_ dual‐mode MRI performance for tumors.

Figure 6e presents the biodistribution of Ca^2+^ in major organs and tumors of 4T1 tumor‐bearing mice after subtracting the original calcium concentration of each tissue post‐injection of MCPNC14‐FH2‐IO3@HA4. The highest content of Ca in tumors is observed at 24 h post‐injection of MCPNC14‐FH2‐IO3@HA4, which aligns with the aforementioned MRI and blood half‐life results. Notably, the high accumulation of MCPNC14‐FH2‐IO3@HA4 in tumors at 24 h (e.g., 15.49 ± 1.31%I.D./g) represents one of the pivotal factors for the marked improvement in the MRI performance of MCPNC14‐FH2‐IO3@HA4 for tumors.

To verify whether the tissue accumulation capacity of NCs is more efficient than that of NSs, calcium peroxide nanosphere (CPNS) was synthesized via the hydrolysis‐precipitation method described in references.^[^ ^62^, ^63^, ^64^, ^65^ ^]^ As illustrated in Figure S31a,b (Supporting Information), CPNS has a regular spherical shape and a uniform particle diameter of 140.5 nm. Subsequently, MCPNC14 and CPNS were respectively surface‐modified with HA to synthesize MCPNC14@HA and CPNS@HA, whose tissue accumulation capacities were assessed following the protocol shown in Figure 6f. Figure 6g shows that the tissue accumulation levels of MCPNC14@HA are all higher than those of CPNS@HA. Specially, the tissue accumulation levels of MCPNC14@HA in spleens and tumor tissues are more significantly elevated than CPNS@HA (^*^ *p* < 0.05), which indicates that MCPNC14@HA indeed possesses superior margination dynamics enabling enhanced penetration through vascular endothelium into tissues. Notably, the hepatic accumulation demonstrates almost no difference between CPNS@HA and MCPNC14@HA nanoparticles. This is because the liver contains the vast majority of macrophages (kupffer cells) in vivo, and liver sinusoidal endothelial cells and hepatic B cells also contribute to nanoparticle clearance.^[^ ^66^, ^67^, ^68^ ^]^ The uptake of nanoparticles by these cells is not strictly dependent on surface modifications and shape of nanoparticles but occurs through physical interception and diverse endocytic pathways, which results in no significant difference in the accumulation of nanoparticles with different shapes in liver.

### 2.7 Antitumor Efficacy Assessment in vivo

**Figure 7:**
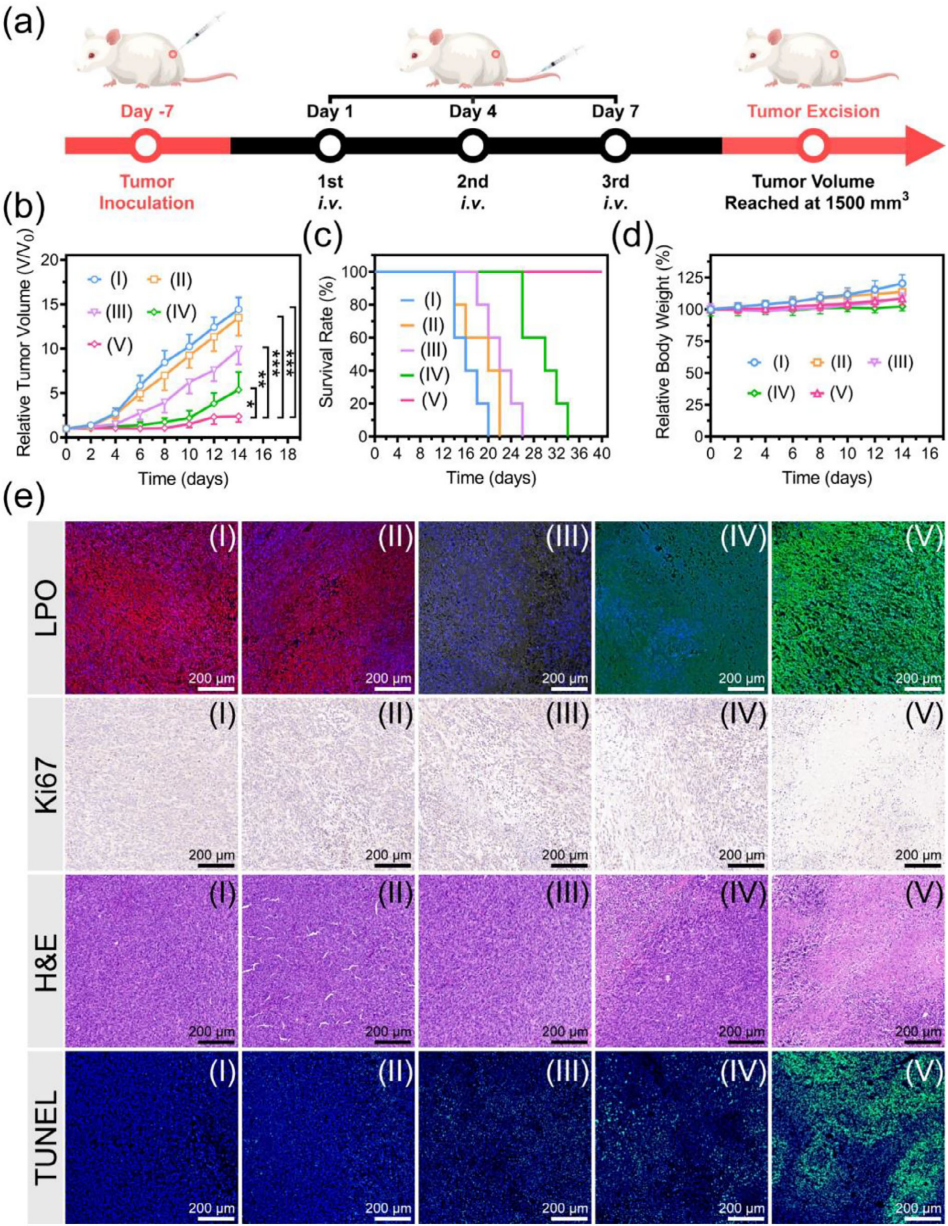
a) Schematic diagram for the timeline of 4T1 tumor inoculation and subsequent treatments. b) The relative tumor volumes of 4T1 tumor‐bearing mice after treatments with saline (group I), MCPNC14 (group II), MCPNC14‐FH2 (group III), MCPNC14‐FH2‐IO3 (group IV), or MCPNC14‐FH2‐IO3@HA4 (group V). Mean ± S.D., *n* = 5. ^*^ *p* < 0.05, ^**^ *p* < 0.01, ^***^ *p* < 0.001. c,d) Survival rates (c), and relative body weights (d) of the mice in groups I–V. e) Histological observation of the tumors with staining of C11‐BODIPY^581/591^, H&E, Ki67 or TUNEL after above‐mentioned treatments (groups I–V).

The antitumor efficacy assessment was conducted in 4T1 tumor‐bearing Balb/c mice with the experimental timeline illustrated in **Figure** **7**a. Figure 7b displays the tumor growth curves of 4T1 tumor‐bearing mice with treatments of the groups I–V. The tumor growth is very rapid in the groups I‐III, and inhibited significantly before 8.0 days post‐injection followed by rapid tumor progression in the group IV. In contrast, the group V exhibits a more significant tumor inhibition effect compared with groups I–IV (^*^ *p* < 0.05, ^**^ *p* < 0.01, ^***^ *p* < 0.001). This phenomenon is attributed to the CD44 receptor‐mediated active tumor targeting capability of MCPNC14‐FH2‐IO3@HA4, which achieves high tumor accumulation and excellent antitumor efficacy. Figure 7c shows the 4T1 tumor‐bearing mice succumbed within 26 days after treatment with saline, MCPNC14, or MCPNC14‐FH2, died within 34 days post‐treatment with MCPNC14‐FH2‐IO3, and all survived for at least 40 days after treatment with MCPNC14‐FH2‐IO3@HA4. Consequently, the post‐treatment survival profiles of mice indicate superior antitumor therapeutic efficacy of MCPNC14‐FH2‐IO3@HA4. Additionally, the body weights of mice increase gradually within 14 days after different treatments (Figure 7d), which indicates the low or negligible toxicity in vivo.

Figure 7e presents the histological observations of tumors stained with LPO, Ki67, H&E, or TUNEL after the aforementioned treatments. Among the groups I–V, MCPNC14‐FH2‐IO3@HA4 treatment (group V) exhibits the most significant LPO production, the most pronounced proliferation inhibition as evidenced by Ki67 images, and the severest apoptosis as observed in H&E and TUNEL images. These findings consolidate the superior tumor therapeutic ability of MCPNC14‐FH2‐IO3@HA4.

### 2.8 Evaluation of in vivo Toxicity

**Figure 8:**
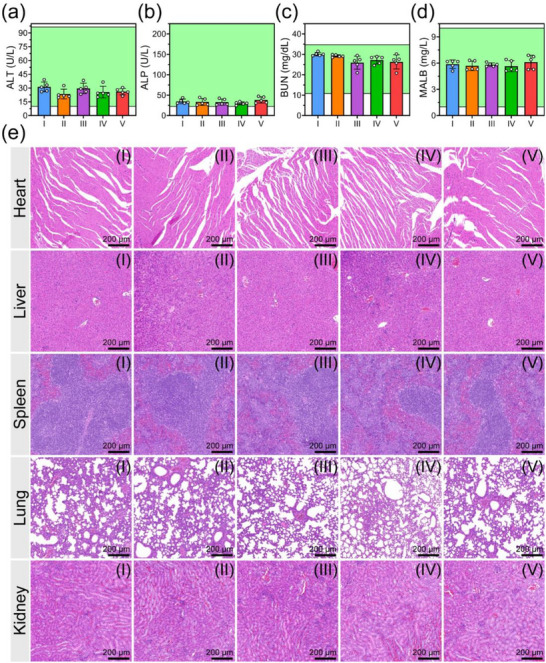
a–d) The contents of liver function markers of alanine aminotransferase (ALT) (a) and alkaline phosphatase (ALP) (b), and kidney function markers of blood urea nitrogen (BUN) (c) and micro albumin (MALB) (d) for 4T1 tumor‐bearing mice after treatments with saline (group I), MCPNC14 (group II), MCPNC14‐FH2 (group III), MCPNC14‐FH2‐IO3 (group IV), or MCPNC14‐FH2‐IO3@HA4 (group V). Ca dosage: 5.0 mg kg^−1^, Mean ± S.D., *n* = 5. e) Representative optical microscopic images of the H&E‐stained heart, liver, spleen, lung, and kidney from 4T1 tumor‐bearing mice with treatments of above‐mentioned groups I–V.

As shown in Figure S32 (Supporting Information), even at a Ca concentration of 80 µg mL^−1^, MCPNC14‐FH2‐IO3@HA4 exhibits a very low hemolysis rate (i.e., 3.1 ± 0.2%), which indicates its excellent blood compatibility. Additionally, the routine hematological parameters, including white blood cell (WBC) count, red blood cell (RBC) count, hemoglobin (HGB) level, mean corpuscular volume (MCV), platelet (PLT) count, lymphocyte (Lym) percentage, hematocrit (HCT), mean corpuscular hemoglobin concentration (MCHC), mean corpuscular hemoglobin (MCH), mean platelet volume (MPV), procalcitonin (PCT), and platelet distribution width (PDW) all remain within normal range after different treatments (Figure S33, Supporting Information). Serum biochemical markers, e.g., liver function indicators of alanine aminotransferase (ALT) and alkaline phosphatase (ALP), and kidney function markers of blood urea nitrogen (BUN) and micro albumin (MALB), are unaffected after treatment with the groups I–V, which indicates normal liver and kidney functions (**Figure** **8**a–d). Microscopic examination of H&E‐stained organs (heart, liver, spleen, lung, and kidney) from 4T1 tumor‐bearing mice with treatment of groups I–V reveal no inflammatory lesions and tissue damage (Figure 8e), which reinforces the high biosafety of MCPNC14‐FH2‐IO3@HA4.

## 3 Conclusion

To augment the capabilities of nanoparticles in drug delivery across biological barriers and enhance their tumor accumulation, in this study, a MCPNC was newly developed through a straightforward hydrolysis precipitation method, which can be utilized for high efficacy tumor therapy by promoting positive feedback loop of Fenton reaction. Typically, the FH and IO loaded MCPNC14 was modified with HA to form MCPNC14‐FH2‐IO3@HA4. The results from morphological (TEM and HAADF‐STEM), compositional (XPS and XRD), and porous structural (BET and BJH) analyses confirm the successful preparation of MCPNC14 and MCPNC14‐FH2‐IO3@HA4. The anti‐tumor efficacy of MCPNC14‐FH2‐IO3@HA4 by promoting the positive feedback loop of Fenton reaction has been verified by in vitro and cell experiments. The specific mechanism is given below: 1) The generation of ROS through the participation of Fe^2^⁺ in Fenton reaction can disrupt mitochondrial membranes and synergistically accelerate the unbalance of mitochondrial homeostasis with Ca^2+^. 2) The loss of mitochondrial membrane potential subsequently activates mitochondrial autophagy signaling in tumor cells, and intracellular endogenous Fe ions stored in the mitochondria are released after mitophagy. 3) The released Fe ions can further participate in the Fenton reaction, and lead to production of more and more ROS, which thereby establish the positive feedback loop of Fenton reaction. Furthermore, the anti‐tumor efficacy of MCPNC14‐FH2‐IO3@HA4 in vivo is demonstrated in 4T1 tumor‐bearing mice. Meanwhile, MCPNC14‐FH2‐IO3@HA4 enables in vivo monitoring of nanoparticle biodistribution and metabolic fate via *T*_1_/*T*_2_ dual mode MRI. The mouse body weight, hemolysis tests, hematological analyses, liver and kidney function assessments, and histological evaluations indicate that MCPNC14‐FH2‐IO3@HA4 exhibits favorable biocompatibility and biosafety.

This study confirms the excellent diagnostic and therapeutic efficacy of MCPNC14‐FH2‐IO3@HA4 on 4T1 tumor‐bearing mice. However, its anti‐metastatic effects and applicability in diverse tumor models remain to be investigated. The MCPNC developed in this work provide critical insights for the nanoparticle shape‐dependent research and will promote the design of novel cubic nanoparticle.

## 4 Experimental Section

### Statistical Analysis

SPSS 25.0 (SPSS, Chicago, USA) was used for statistical analysis. All experimental data were demonstrated as Mean ± S.D. of at least three independent replicate experiments. The group‐specific sample size (n) is illustrated in the figure legends. Statistical analyses utilized Student's *t*‐tests for two‐group comparisons and one‐way ANOVA with Tukey's post hoc testing for three or more groups, with significance thresholds defined as ^*^ *p* < 0.05, ^**^ *p* < 0.01, or ^***^ *p* < 0.001.

## Conflict of Interest

The authors declare no conflict of interest.

## Author Contributions

Q.F. and M.W. contributed equally to this work. Q.F. performed conceptualization, data curation, formal analysis, investigation, methodology, software, visualization, and writing – original draft. M.W. performed formal analysis, investigation, methodology, validation, visualization, and writing – original draft. J.L. performed conceptualization, formal analysis, funding acquisition, investigation, resources, supervision, and writing – review & editing. Y.H. performed formal analysis, investigation, methodology, validation, and visualization. J.Y. performed formal analysis, investigation, validation, and visualization. J.Z. performed methodology, validation, and visualization. B.R. performed investigation, validation, and visualization. L.S. performed methodology, validation, and visualization. Z.L. performed formal analysis, investigation, and visualization. A.L. performed formal analysis and methodology. W.X. performed formal analysis and visualization. Z.W. performed validation and visualization. L.H. performed formal analysis and validation. C.Y. performed methodology and resources. G.W. performed methodology and resources. Z.C. performed conceptualization, investigation, methodology, resources, supervision, and writing – review & editing. X.C. performed conceptualization, formal analysis, investigation, supervision, and writing – review & editing. Z.S. performed conceptualization, formal analysis, funding acquisition, investigation, methodology, project administration, resources, supervision, and writing – review & editing.

## Supporting information



Supporting Information

## Data Availability

The data that support the findings of this study are available in the supplementary material of this article.
