# Supplementary material for: A Mesoporous Calcium Peroxide Nanocuboid with High Tumor Accumulation Across Biological Barriers for High Efficacy Tumor Therapy
Source: Adv Sci (Weinh). 2025 Jul 20;12(39):e10778. doi: 10.1002/advs.202510778 (PMC12533144; doi:10.1002/advs.202510778)
Supplement: Supplementary file 1 — Supporting Information [file ADVS-12-e10778-s001.docx]

**Supporting Information**

**A Mesoporous Calcium Peroxide Nanocuboid with High Tumor Accumulation Across Biological Barriers for High Efficacy Tumor Therapy**

Qingdeng Fan,^#^ Min Wang,^#^ Jie Lin,* Ya Huang, Jing Yang, Jiaoyang Zhu, Bin Ren, Li Sun, Zongheng Li, Aochi Liu, Wei Xiong, Zhenni Wei, Lin Huang, Chenggong Yan, Ge Wen, Zhao Chen,* Xiaoyuan Chen,* Zheyu Shen*

Dr. Q. Fan, Y. Huang, Dr. J. Yang, J. Zhu, B. Ren, Dr. Z. Li, Dr. L. Huang, Prof. Z. Shen

School of Biomedical Engineering, Southern Medical University, 1023 Shatai South Road, Baiyun, Guangzhou, Guangdong 510515, China.

Dr. Min Wang, Dr. W. Xiong, Prof. C. Yan, Prof. G. Wen, Prof. Z. Chen

Medical Imaging Center, Nanfang Hospital, Southern Medical University, 1023 Shatai South Road, Baiyun, Guangzhou, Guangdong 510515, China.

Prof. J. Lin, Dr. L. Sun, Mr. A. Liu

Ningbo Institute of Materials Technology and Engineering, CAS, 1219 Zhongguan West Road, Ningbo 315201, P. R. China

Dr. Z. Wei, Prof. X. Chen

Departments of Diagnostic Radiology, Surgery, Chemical and Biomolecular Engineering, and Biomedical Engineering, Clinical Imaging Research Centre, Nanomedicine Translational Research Program, Yong Loo Lin School of Medicine and College of Design and Engineering, National University of Singapore, Singapore 119228, Singapore.

**Corresponding Authors**

*^*^*E-mail: sz@smu.edu.cn (Z.S.); chen.shawn@nus.edu.sg (X.C.); chenzhao0418@smu.edu.cn (Z.C.); linjie@nimte.ac.cn (J.L.)

**Author Contributions**

^#^Q.F. and M.W. contributed equally to this work.

**Experimental Section**

***Materials and Reagents:*** Calcium chloride anhydrous (CaCl_2_), hydrogen peroxide (H_2_O_2_), and hyaluronic acid (HA, M.W. = 10-20 KDa) were purchased from Sinopharm Chemical Reagent (Shanghai, China). Dimethyl sulfoxide (DMSO, ≥ 99.7 %), ethanol absolute (CH_3_CH_2_OH, ≥ 99.7 %), ammonia solution (≥ 28 %), 5,5’-dithiobis-2-(nitrobenzoicacid) (DTNB), 3,3’,5,5’-Tetramethylbenzidine (TMB), fluorescein isothiocyanate (FITC) and sodium hydroxide (NaOH, ≥ 96 %) were purchased from Macklin Reagent (Shanghai, China). Poly (acrylic acid) (PAA, *M*w = 2,100) and iron chloride (FeCl_3_, ≥ 97%) were purchased from Sigma-Aldrich (USA). Iron sulfate heptahydrate (FeSO_4_·7H_2_O) was purchased from Acros Organics (USA). 1,2-Bis(2-aminophenoxy)ethane-N,N,N’,N’-tetraacetic acid acetoxymethyl ester (BAPTA-AM) and ferroheme (FH, ≥ 99.7 %) were purchased from MedChemExpress (Shanghai, China). Dulbecco’s modified Eagle’s medium (DMEM), penicillin-streptomycin, fetal bovine serum (FBS), trypsin, 3-[4,5-dimethylthiazol-2-yl]-2,5-diphenyltetrazolium bromide (MTT) and phosphate buffered saline (PBS) were purchased from Invitrogen (USA). 5,5’,6,6’-tetrachloro-1,1’,3,3’-tetraethylbenzimidazolylcarbocyanine iodide (JC-1) Assay Kit, GSH Assay Kit, Cell Plasma Membrane Staining Kit with 3,3’-dioctadecyloxacarbocyanine (DiO), 2-(4-Amidinophenyl)-6-indolecarbamidine dihydrochloride (DAPI), DNA Damage Assay Kit by γ-H2AX Immunofluorescence, DCFH-DA, Endo/Lyso-Tracker Green, Alexa Fluor™ 555-Labeled Donkey Anti-Rabbit IgG (H + L), and Glutathione peroxidase 4 (GPX4) ELISA kit were purchased from Beyotime Biotechnology (Shanghai, China). Calcein acetoxymethyl ester/propidium iodide (Calcein-AM/PI) solution was purchased from Dalian Meilun Biotech Co., Ltd (China). Deferoxamine mesylate salt (DFO) and polyvinyl pyrrolidone (PVP, M.W. = 40 KDa) were purchased from Shanghai yuanye Bio-Technology Co., Ltd (China).

***Synthesis of MCPNC:*** MCPNC was synthesized by a hydrolysis precipitation method. Typically, 1.0 mL of CaCl_2_ solution (400-25.0 mg/mL in pure water) was fed into 15 mL of ethanol (99.7-50 % in pure water) under stirring until fully dissolved. After that, 1.0 mL of NaOH solution (0.50-4.0 M) was added and stirred for 10 min. 1.5 mL of H_2_O_2_ solution (30-2.5 %) was then added dropwise to the above mixture and stirred for 24 h. The resulting nanoparticles were respectively washed with ethanol and Milli-Q-water, and separated by centrifugation (15,000 × g, 15 min). Finally, the product was kept in a fridge for next use.

***Synthesis of MCPNC14-FH:*** 0.32-0.02 mL of FH solution in H_2_O (2.0 mg/mL) and 3.68-3.98 mL pure water were added into 1.0 mL of MCPNC14 dispersed in pure water (*C*_Ca_ = 25 mM). The mixture was stirred for 24 h at room temperature. Subsequently, the obtained MCPNC14-FH dispersion was washed using Milli-Q water and separated by centrifugation (15,000 × g, 15 min) to remove the unloaded FH. The finally obtained product in 5.0 mL of Milli-Q water was stored at 4.0 ^o^C for further use.

***Synthesis of IO:*** Exceedingly small magnetic iron oxide nanoparticles (IO) were synthesized by a coprecipitation method. Typically, 40 mL of PAA solution (4.0 mg/mL) was magnetically stirred at room temperature under N_2_ protection for 1.0 h to remove oxygen, and then heated to reflux (100 ^o^C). After that, 0.80 mL of iron precursors (500 mM of FeCl_3_ plus 250 mM of FeSO_4_) was quickly injected into the above solution, followed by addition of 12 mL of ammonia solution (28 %). The reaction was kept at 100 ^o^C for 30 min under magnetic stirring. Finally, the obtained IO dispersions were cooled down to room temperature. The resultant IO was dialyzed (MwCO = 3.5 kDa) in deionized water for 3.0 days with water change twice a day to remove the unreacted reactants. The purified IO was concentrated by a rotary evaporator. The Fe concentrations of the IO dispersions were detected by an inductively coupled plasma optical emission spectrometry (ICP-OES) (iCAP PRO, Thermo Fisher Scientific, USA).

***Synthesis of MCPNC14-FH2-IO:*** 0.58-0.04 mL of IO dispersion in H_2_O (*C*_Fe_ = 20 mM) and 3.42-3.96 mL pure water were added into 1.0 mL of MCPNC14-FH2 dispersed in pure water (*C*_Ca_ = 25 mM) under stirring at room temperature. After 24 h, the obtained MCPNC14-FH2-IO dispersion was washed using Milli-Q water and separated by centrifugation (15,000 × g, 15 min) to remove the unloaded IO. The finally obtained product was kept in a fridge for next use.

***Synthesis of MCPNC14-FH2-IO3@HA:*** 0.50-0.05 mL of HA solution in H_2_O (2.0 mg/mL) and 3.50-3.95 mL pure water were added into 1.0 mL of MCPNC14-FH2-IO3 dispersed in pure water (*C*_Ca_ = 25 mM). The mixture was stirred for 24 h at room temperature. The obtained MCPNC14-FH2-IO3@HA dispersion was then washed using Milli-Q water and separated by centrifugation (15,000 × g, 15 min) to remove the unloaded HA. Finally, the obtained product in 5.0 mL of Milli-Q water was stored at 4.0 ^o^C for further use.

***Synthesis of R6G-Loaded MCPNC14-FH2-IO3@HA4:*** 4.0 mL of MCPNC14-FH2-IO3@HA4 (*C*_Ca_ = 2.0 mM) were mixed with 0.70 mL of R6G (10 μM) under magnetic stirring at room temperature. After 24 h, the obtained R6G-MCPNC14-FH2-IO3@HA4 dispersion was washed using Milli-Q water by centrifugation (15,000 × g, 15 min) to remove the unloaded R6G. The finally obtained R6G-MCPNC14-FH2-IO3@HA4 was redispersed in 4.0 mL of Milli-Q water, and kept in a fridge for next use.

***Synthesis of CPNS:*** CPNS was synthesized by a hydrolysis precipitation method. Typically, 1.0 mL of CaCl_2_ solution (100 mg/mL in pure water) was fed into 15 mL of ethanol (99.7 %) under stirring until fully dissolved. Then 1.0 mL of NH_3_·H_2_O solution (1.0 M) was added and stirred for 10 min. 1.5 mL of H_2_O_2_ solution (10 %) was then added dropwise to the above mixture and stirred for 12 h. The resulting nanoparticles were respectively washed with ethanol and Milli-Q-water, and separated by centrifugation (15,000 × g, 15 min). Finally, the product was kept in a fridge for next use.

***Synthesis of MCPNC14@HA:*** 0.20 mL of HA solution in H_2_O (2.0 mg/mL) and 3.8 mL pure water were added into 1.0 mL of MCPNC14 dispersed in pure water (*C*_Ca_ = 25 mM). The mixture was stirred for 24 h at room temperature. The obtained MCPNC14@HA dispersion was then washed using Milli-Q water and separated by centrifugation (15,000 × g, 15 min) to remove the unloaded HA. Finally, the obtained product in 5.0 mL of Milli-Q water was stored at 4.0 ^o^C for further use.

***Synthesis of CPNS@HA:*** 0.20 mL of HA solution in H_2_O (2.0 mg/mL) and 3.8 mL pure water were added into 1.0 mL of CPNS dispersed in pure water (*C*_Ca_ = 25 mM). The mixture was stirred for 24 h at room temperature. The obtained CPNS@HA dispersion was then washed using Milli-Q water and separated by centrifugation (15,000 × g, 15 min) to remove the unloaded HA. Finally, the obtained product in 5.0 mL of Milli-Q water was stored at 4.0 ^o^C for further use.

***Characterizations:*** Transmission electron microscope (TEM) was used to observe the morphology and structure of the nanoparticles. X-ray photoelectron spectroscopy (XPS) was utilized to determine the valence states of elements for the nanoparticles. X-ray diffraction (XRD) patterns were obtained with the parameters set as Cu Kα, 40 mA, and 40 kV. The size and zeta potential of nanoparticles were measured using an instrument of dynamic light scattering (DLS) (Nano-Brook 90PlusZata, Brookhaven, USA). Nitrogen absorption-desorption isotherm and porosity were measured by a surface area analyzer (QuadraSorb SI 2000-08, Quantachrome Instruments, USA). UV-vis spectra were measured using a spectrophotometer (Evolution 300, Thermo Fisher, USA). Fourier transform infrared (FT-IR) spectra were recorded using a Nicolet 6700 spectrometer (Thermo Electron Corporation, Madison, WI). The concentration of Ca or Fe ions was determined by inductively coupled plasma-optical emission spectrometer (ICP-OES) (iCAP PRO, Thermo Fisher Scientific, USA). The cell viability was measured by a multi-mode microplate reader using MTT method (Synergy H1, BioTek Instruments, USA). The stained cells were observed by laser scanning confocal microscopy (LSCM) (ECLIPSE Ti2, Nikon, Japan). The cells after treatment were measured by flow cytometry (BD FACSAria III, BD Biosciences, USA). The magnetic resonance imaging (MRI) efficiency of nanoparticles was measured by a Bruker MRI scanner (7.0 T, B-C 70/16, Bruker, USA), or a clinical MRI scanner system (3.0 T, Ingenia, Philips, Netherlands).

***MRI Performance in Vitro:*** The MR imaging and relaxation times of the nanoparticles were measured by a clinical MRI scanner system (3.0 T, Ingenia, Philips, Netherlands). The relaxivity values of *r*_1_ and *r*_2_ were obtained as the slopes from the linear curves of relaxation rate (1/*T*_1_ or 1/*T*_2_) versus the Fe concentration (mM). Meanwhile, MR images were analyzed by measuring signal intensity with the software Image J. The signal-to-noise ratio (SNR) and ΔSNR value (*i.e.*, signal enhancement) were calculated according to the following equations:

SNR = SI_mean_/SD_noise_

ΔSNR = (SNR_sample_ - SNR_control_)/SNR_control_ × 100 %

***Cell Culture:*** 4T1 cells (mouse breast cancer cell line) were cultured in the complete DMEM culture medium supplemented with 10 % of FBS, 100 U/mL of penicillin G sodium, and 100 mg/mL of streptomycin sulfate. All of the cells were incubated at 37 ^o^C in a humidified atmosphere containing 5.0 % of CO_2_.

***Cellular Uptake:*** Cellular uptake of nanoparticles was measured *via* LSCM, flow cytometry, and MRI.

By LSCM: 4T1 cells were cultured in confocal dishes at a density of 1.0 × 10^4^ cells/mL for 24 h. After that, the growth media were replaced with 0.50 mL of fresh one without (control group) or with R6G-MCPNC14-FH2-IO3@HA4 (*C*_Ca_ = 40 μg/mL). After 4.0 h of incubation, the treated cells were washed with PBS twice, fixed with 4.0 % of paraformaldehyde for 30 min, permeabilized with 0.10 % of Triton X-100 for 5.0 min, blocked with 1.0 % of BSA for 30 min, and then stained with a mixture of DAPI and Phalloidin-FITC solution for 30 min at room temperature. Finally, the cells were observed by a LSCM (Nikon ECLIPSE Ti2).

By Flow Cytometry: 4T1 cells were cultured in a 6-well plate (5.0 × 10^5^ cells per well), and incubated without (control group) or with R6G-MCPNC14-FH2-IO3@HA4 for 4.0 h (*C*_Ca_ = 40 μg/mL). The treated cells were washed with cold PBS twice, trypsinized and harvested by centrifugation at 800 × g for 3.0 min. The cells were resuspended in 200 μL of PBS and then detected by flow cytometry (BD FACSAria III, USA).

By MRI: 4T1 cells were seeded in 6-well plates at a density of 5.0 × 10^5^ cells per well, cultured for 24 h, and then treated with MCPNC14-FH2-IO3@HA4 (*C*_Ca_ = 40 μg/mL) for different times (1.0, 2.0, or 4.0 h) at 37 ^o^C. Next, the cells were harvested in 0.5 mL of centrifuge tubes, and 0.20 mL of agarose solution (0.80 wt%) was added to fix the cells. *T*_1_-weighted MR images were acquired using a 3.0 T MRI scanner under following parameters: TE = 8.2 ms, TR = 500 ms.

***Lysosome Escape Assay:*** 0.50 mL of 4T1 cells in complete growth medium were seeded into confocal dishes at a density of 1.0 × 10^4^ cells/mL, and allowed to adhere at 37 ^o^C for 24 h. And then, the growth medium was replaced with a fresh one (without FBS) with R6G-MCPNC14-FH2-IO3@HA4 (*C*_Ca_ = 40 μg/mL) for 6.0 h. After that, the cells were washed thrice with PBS and then the nuclei of cells were stained with DAPI, while lysosomes of cells were labeled with Lyso-Tracker. The cells were then immersed in PBS and directly observed by a LSCM (Nikon ECLIPSE Ti2).

***Detection of Intracellular GSH:*** For LSCM analysis, 4T1 cells (1.0 × 10^5^ cells) were first seeded into confocal dishes, and incubated overnight to achieve adherence. The culture medium was replaced with fresh one (without FBS) without (control group) or with MCPNC14, MCPNC14-FH2, MCPNC14-FH2-IO3, or MCPNC14-FH2-IO3@HA4 (the concentration of Ca^2+^ used was 40 μg/mL in all treatment groups). After 12 h of incubation, the treated cells were washed with PBS, fixed with 4.0 % of paraformaldehyde for 30 min, permeabilized with 0.10 % Triton X-100 for 5.0 min, blocked with 1.0 % BSA for 30 min. The cells were then stained with DAPI for 30 min, and ThiolTracker™ Violet kit (10 μM) for 30 min. Finally, the cells were observed by LSCM (Nikon ECLIPSE Ti2).

For flow cytometry analysis, 1.0 mL of 4T1 cells in complete growth medium were seeded into each well of a 6-well culture plate with a cell density of 5.0 × 10^5^ cells/mL, and allowed to adhere at 37 ^o^C for 24 h. After that, the culture medium was replaced with fresh one (without FBS) without (control group) or with MCPNC14, MCPNC14-FH2, MCPNC14-FH2-IO3, or MCPNC14-FH2-IO3@HA4 (the concentration of Ca^2+^ used was 40 μg/mL in all treatment groups). After 12 h of incubation at 37 ^o^C, the culture media were removed, and the cells were washed thrice with PBS. After that, 2.0 mL of fresh culture media containing 10 μM of ThiolTracker™ Violet kit were added to each well, and the cells were cultured at 37 °C for 30 min. The cells were washed thrice with PBS, treated with trypsin for 3.0 min, and then centrifuged at 800 × g for 3.0 min. The obtained cells were resuspended in 0.20 mL of PBS and then measured by a flow cytometer (BD FACSAria III, USA). Data analysis was performed using the flow cytometry analysis software (FlowJo V10.8.1).

***Immunofluorescence Analysis of GPX4 Protein in Cells:*** 4T1 cells were seeded into confocal dishes and cultured for 24 h. Next, the original medium was replaced with the fresh DMEM (without FBS) without (control group) or with MCPNC14, MCPNC14-FH2, MCPNC14-FH2-IO3, or MCPNC14-FH2-IO3@HA4 (the concentration of Ca^2+^ used was 40 μg/mL in all treatment groups). After 12 h of incubation, the cell samples were fixed with 4.0 % paraformaldehyde, permeabilized with 1.0 % Triton X-100, blocked with 3.0 % BSA, and then incubated with Rabbit Anti-GPX4 antibody solution (dilution 1:1000) overnight. The obtained cells were washed with PBS, and further incubated with Alexa Fluor™ 555-Labeled Donkey Anti-Rabbit IgG (H + L) antibody (Beyotime Biotechnology) for 30 min. The nuclei were stained by DAPI for 30 min. Finally, the samples were observed by the LSCM imaging system (Nikon ECLIPSE Ti2).

***Measurement of Intracellular GPX4 Activity:*** The GPX4 activity was measured by the enzyme-linked immunosorbent assay (ELISA) kit (S0058, Beyotime, China). Briefly, 4T1 cells were seeded in 6-well plates at a density of 5.0 × 10^5^ cells/well to achieve adherence. The culture medium was replaced with fresh one (without FBS) without (control group) or with MCPNC14, MCPNC14-FH2, MCPNC14-FH2-IO3, or MCPNC14-FH2-IO3@HA4 (the concentration of Ca^2+^ used was 40 μg/mL in all treatment groups). After 12 h of incubation, the GPX4 activity was measured according to the manufacturer’s protocol using a multi-mode microplate reader (Synergy H1, BioTek Instruments, USA).

***Evaluation of Intracellular ROS Generation via DCFH-DA Assay:*** The intracellular generation of ROS was determined by 2,7-dichlorofluorescein diacetate (DCFH-DA), which could be oxidized to the highly fluorescent dichlorofluorescein (DCF) by ROS.

By LSCM: 0.50 mL of 4T1 cells in complete growth medium were seeded into confocal dishes at a density of 1.0 × 10^4^ cells/mL, and allowed to adhere at 37 ^o^C for 24 h. After that, the growth medium was replaced with a fresh one (without FBS) without (control group) or with MCPNC14, MCPNC14-FH2, MCPNC14-FH2-IO3, or MCPNC14-FH2-IO3@HA4 (the concentration of Ca^2+^ used was 40 μg/mL in all treatment groups). After 12 h of incubation at 37 ^o^C, the culture media were removed and the cells were washed thrice with PBS. After that, 0.50 mL of fresh culture media containing 20 μM of DCFH-DA were added to each well, and the cells were cultured at 37 °C for 30 min. The cells were washed thrice with PBS, and then fixed with 4.0 % of paraformaldehyde for 30 min. After that, the LSCM images of the samples were observed on a LSCM imaging system (Nikon ECLIPSE Ti2).

By Flow Cytometry: 1.0 mL of 4T1 cells in complete growth medium were seeded into each well of a 6-well culture plate with a cell density of 5.0 × 10^5^ cells/mL, and allowed to adhere at 37 ^o^C for 24 h. After that, the growth medium was replaced with a fresh one (without FBS) without (control group) or with MCPNC14, MCPNC14-FH2, MCPNC14-FH2-IO3, or MCPNC14-FH2-IO3@HA4 (the concentration of Ca^2+^ used was 40 μg/mL in all treatment groups). After 12 h of incubation at 37 ^o^C, the culture media were removed, and the cells were washed thrice with PBS. After that, 2.0 mL of fresh culture media containing 20 μM of DCFH-DA were added to each well, and the cells were cultured at 37 °C for 30 min. The cells were washed thrice with PBS, treated with trypsin for 3.0 min, and then centrifuged at 800 × g for 3.0 min. The obtained cells were resuspended in 0.20 mL of PBS and then measured by a flow cytometer (BD FACSAria III, USA). Data analysis was performed using the flow cytometry analysis software (FlowJo V10.8.1).

***Observation of Cell Membrane Integrity:*** 4T1 cells were seeded into confocal dishes (1.0×10^5^ cells/well), and incubated for 24 h. The culture media were replaced with fresh one (without FBS) without (control group) or with MCPNC14, MCPNC14-FH2, MCPNC14-FH2-IO3, or MCPNC14-FH2-IO3@HA4 (the concentration of Ca^2+^ used was 40 μg/mL in all treatment groups). After 12 h of incubation, the cells were washed with PBS, fixed with 4.0 % of paraformaldehyde for 30 min, permeabilized with 0.10 % Triton X-100 for 5.0 min, blocked with 1.0 % BSA for 30 min. The cells were then stained with DAPI for 30 min, and DiO (1.0 mM) for 30 min. Finally, the cells were observed by LSCM (Nikon ECLIPSE Ti2).

For flow cytometry analysis, 4T1 cells were seeded into 6-well plates (5.0×10^5^ cells/well), and incubated for 24 h. The culture media were replaced with fresh one (without FBS) without (control group) or with MCPNC14, MCPNC14-FH2, MCPNC14-FH2-IO3, or MCPNC14-FH2-IO3@HA4 (the concentration of Ca^2+^ used was 40 μg/mL in all treatment groups). After 12 h of incubation, the cells were cultured with DiO (1.0 mM) for 30 min. The cells were washed twice with PBS, and harvested by trypsinization and centrifugation (800 × g, 3.0 min). The obtained cells were re-suspended in PBS, and analyzed using flow cytometry.

***Detection of Mitochondrial Membrane Potential:*** The mitochondrial membrane potential was detected using JC-1 probe (Beyotime Biotechnology, Shanghai, China). Briefly, 4T1 were seeded in a confocal dish with 1.0 × 10^5^ cells/dish to achieve adherence. The culture medium was replaced with fresh one (without FBS) without (control group) or with MCPNC14, MCPNC14-FH2, MCPNC14-FH2-IO3, or MCPNC14-FH2-IO3@HA4 (the concentration of Ca^2+^ used was 40 μg/mL in all treatment groups). After 12 h of incubation, the cells were washed with PBS, and stained with the JC-1 (10 μg/mL) probe for 30 min. Then cells were then rinsed thrice with PBS, and observed by LSCM. A green channel with a 488 nm of excitation wavelength was utilized for JC-1 monomers, while a red channel with a 546 nm of excitation wavelength was utilized for JC-1 aggregates.

***Detection of Lipid Peroxides (LPO):*** LPO was typically detected by a C11-BODIPY^581/591^ fluorescence probe, which can be observed by LSCM and evaluated by flow cytometry. First, 4T1 cells were seeded in 6-well plates at a density of 5.0 × 10^5^ cells per well, and cultured for 24 h. After that, the growth medium was replaced with a fresh one (without FBS) without (control group) or with MCPNC14, MCPNC14-FH2, MCPNC14-FH2-IO3, or MCPNC14-FH2-IO3@HA4 (the concentration of Ca^2+^ used was 40 μg/mL in all treatment groups). After 12 h of incubation at 37 ^o^C, the cells were washed thrice with PBS, and incubated with a fresh DMEM containing C11-BODIPY^581/591^ (5.0 μM) for 30 min. Finally, cells were washed with PBS, and then subjected to LSCM observation and flow cytometry evaluation.

***Live/Dead Cells Staining:*** 4T1 cells were cultured in confocal dishes at a density of 1.0 × 10^4^ cells/mL for 24 h. As control groups, the cells were pretreated with BAPTA-AM (50 μM) and/or DFO (1.0 mM) for 1.0 h to block the occurrence of ferroptosis and/or calcicoptosis. The cells were incubated with a fresh DMEM medium (without FBS) without (control group) or with MCPNC14, MCPNC14-FH2, MCPNC14-FH2-IO3, or MCPNC14-FH2-IO3@HA4 (the concentration of Ca^2+^ used was 40 μg/mL in all treatment groups) for another 24 h. Thereafter, the cells were stained with Calcein-AM (5.0 μg/mL) and PI (10 μg/mL) for 30 min. Finally, the cells were observed by LSCM.

By Flow Cytometry: The live/dead cells were quantitatively analyzed by flow cytometry after Calcein-AM and PI staining. The cells were seeded in 6-well plates with a density of 5.0 × 10^5^ cells per well, and then treated without (control group) or with MCPNC14, MCPNC14-FH2, MCPNC14-FH2-IO3, or MCPNC14-FH2-IO3@HA4 (the concentration of Ca^2+^ used was 40 μg/mL in all treatment groups) for 24 h. The cells were then digested, and stained with 200 μL of mixture of PI (10 μg/mL) and Calcein-AM (5.0 μg/mL) for 30 min in darkness. The live/dead cells were quantified by flow cytometry, and analyzed using the FlowJo software.

***MTT Assay:*** Cell viabilities were measured using the MTT assay. Typically, 150 μL of 4T1 cells in the complete DMEM medium were seeded into each well of 96-well plates at a concentration of 1.0 × 10^4^ cells/mL, and allowed to adhere overnight. The growth medium was replaced with a fresh DMEM medium without FBS containing various concentrations of MCPNC14, MCPNC14-FH2, MCPNC14-FH2-IO3, or MCPNC14-FH2-IO3@HA4 (the concentration of Ca^2+^ used was 40 μg/mL in all treatment groups). After 24 h of incubation at 37 °C, the growth medium was replaced with a complete medium, and 10 μL of MTT (5.0 mg/mL in PBS) was added to each well of the 96-well plates. After an additional 4.0 h of incubation, the growth medium was removed and the resulted formazan crystals in each well were dissolved in 100 μL of DMSO. The absorbance was recorded at a wavelength of 490 nm using a multi-mode microplate reader (Synergy H1, BioTek Instruments, USA).

To provide further evidence for the ferroptosis and/or calcicoptosis therapy of cancer cells induced by MCPNC14-FH2-IO3@HA4 nanoparticles, blocking studies were carried out using a chelator of Ca ions (*i.e.*, BAPTA-AM) and/or a chelator of Fe ions (*i.e.*, DFO). Briefly, before being treated with MCPNC14-FH2-IO3@HA4 nanoparticles, the cells were pretreated with BAPTA-AM (50 μM) and/or DFO (1.0 mM) for 1.0 h to chelate Ca and/or Fe ions. Other steps were same with the above-mentioned procedures.

***Tumor Model:*** All animal procedures were performed in accordance with the Guidelines for Care and Use of Laboratory Animals of Southern Medical University, and approved by the Animal Ethics Committee of Southern Medical University. The assigned approval/accreditation number is SYXK(YUE)2021-0167.

To establish the xenograft tumor models, female Balb/c mice (five-week-old, 15-20 g) were subcutaneously implanted 100 μL of 5.0 × 10^6^ 4T1 cells in the right back side. The size of tumors was measured every other day with a vernier caliper, and the tumor volumes were calculated as follows: tumor volume (mm^3^) = width^2^ × length / 2.

***In Vivo Pharmacokinetics and Biodistribution of Nanoparticles:*** For the pharmacokinetic analysis, 4T1 tumor-bearing Balb/c mice were injected with 100 µL of MCPNC14 or MCPNC14-FH2-IO3@HA4 (Ca dosage is 5.0 mg/kg) *via* the tail-vein. After injection, venous blood in the orbit of mice was collected at different times, and the concentration of Ca^2+^ was determined using ICP-OES.

To investigate the biodistribution of nanoparticles *in vivo*, the 4T1 tumor-bearing Balb/c mice were administrated with 100 µL of MCPNC14-FH2-IO3@HA4 (Ca dosage is 5.0 mg/kg) intravenously. At 6.0, 12, 24, 36, 48, or 72 h post-injection, the major organs (heart, liver, spleen, lung, and kidney) and tumors were collected and digested with concentrated nitric acid. The Ca^2+^ contents in all samples were measured by ICP-OES.

***In Vivo MRI Examination:*** *In vivo* *T*_1_-MRI evaluation of MCPNC14-FH2-IO3@HA4 or commercial Magnevist^®^ was performed using a 7.0 T Bruker MRI scanner. The parameters were shown as: TR/TE = 500/8.4 ms, FOV = 40 × 40 mm^2^, matrix size = 256 × 256, number of averages = 4, scan duration = 232 s. 4T1 tumor-bearing Balb/c mice were injected with the contrast agents MCPNC14-FH2-IO3@HA4 (Ca dosage is 5.0 mg/kg) or commercial Magnevist^®^ (Gd dosage is 5.0 mg/kg) intravenously. *T*_2_-weighted MR images were acquired pre-injection, and post-injection (intravenously) of MCPNC14-FH2-IO3@HA4. Multi-slice multi-echo sequence was employed to acquire images using parameters as follows: repetition time (TR) = 6000 ms, echo time (TE) = 120 ms, matrix size = 256 × 256, field of view = 40 × 40 mm^2^, slices = 9, slice thickness = 1.0 mm. MR images were analyzed by measuring signal intensity with the software Image J.

***Tumor Therapy Performance:*** When 4T1 tumor-bearing mice grew to a volume of ~100 mm^3^, the mice were randomly divided into five groups (*n* = 5), and treated with saline, MCPNC14, MCPNC14-FH2, MCPNC14-FH2-IO3 or MCPNC14-FH2-IO3@HA4 (Ca dosage is 5.0 mg/kg) *via* tail-vein injection. The tumor volume and body weight were recorded every other day.

The tumors were dissected and stained by hematoxylin and eosin (H&E), terminal deoxynucleotidyl transferase (TdT)-mediated deoxyuridine triphosphate (dUTP)-biotin nick-end labeling (TUNEL), Ki-67 or boron-dipyrromethene C11-BODIPY^581/591^ for histological analysis.

***Hemolysis Analysis:*** Hemolysis analysis was performed to examine the hemocompatibility of the MCPNC14-FH2-IO3@HA4. Typically, 1.5 mL of blood collected from the inner canthus vein plexus of mice was diluted with 3.5 mL of PBS, followed by centrifugation and washing to obtain the pure red blood cells (RBCs) suspended in 5.0 mL of PBS solution. Thereafter, 100 μL of RBC suspension was mixed with 900 μL of PBS (negative control), water (positive control), or MCPNC14-FH2-IO3@HA4 dispersed in PBS with various concentrations of Ca (2.5, 5.0, 10, 20, 40, or 80 μg/mL). After 2.0 h of incubation at 37 ^o^C, all the samples were centrifuged at 1,000 × g for 5.0 min. Then, the photos of the samples were taken, and the absorbance of the obtained supernatants at 540 nm was measured using a UV-vis spectrophotometer. The hemolysis rate was calculated according to the following formula: hemolysis rate (%) = [(Ab_sample_-Ab_negative control_)/(Ab_positive control_-Ab_negative control_)] × 100 %.

***Ethic Approval*:** All animal procedures were performed in accordance with the guidelines for Care and Use of Laboratory Animals of Southern Medical University, and approved by the Animal Ethics Committee of Southern Medical University. The assigned approval/accreditation number is SYXK (YUE) 2021-0167.

**Table S1.** Synthesis conditions and characterization results of MCPNC1-17.

| Sample | *C*_PVP_ ^a^  (μM) | *C*_CaCl2_ ^b^  (mg/mL) | *C*_NaOH_ ^c^  (M) | *C*_H2O2_ ^c^  (%) | *C*_Ethanol_ ^d^  (%) | Average Particle Size ^e^ (nm) |
| --- | --- | --- | --- | --- | --- | --- |
| MCPNC1 | 2000 | 400 | 0.5 | 30 | 99.7 | Precipitation |
| MCPNC2 | 1500 | 400 | 0.5 | 30 | 99.7 | 397.8 |
| MCPNC3 | 1000 | 400 | 0.5 | 30 | 99.7 | 344.2 |
| MCPNC4 | 500.0 | 400 | 0.5 | 30 | 99.7 | 327.2 |
| MCPNC5 | 250.0 | 400 | 0.5 | 30 | 99.7 | 346.4 |
| MCPNC6 | 500.0 | 200 | 0.5 | 30 | 99.7 | 325.3 |
| MCPNC7 | 500.0 | 100 | 0.5 | 30 | 99.7 | 315.5 |
| MCPNC8 | 500.0 | 50.0 | 0.5 | 30 | 99.7 | No Nanocuboid |
| MCPNC9 | 500.0 | 25.0 | 0.5 | 30 | 99.7 | No Product |
| MCPNC10 | 500.0 | 100 | 1.0 | 30 | 99.7 | 237.4 |
| MCPNC11 | 500.0 | 100 | 2.0 | 30 | 99.7 | 347.4 |
| MCPNC12 | 500.0 | 100 | 4.0 | 30 | 99.7 | No Nanocuboid |
| MCPNC13 | 500.0 | 100 | 1.0 | 20 | 99.7 | 195.6 |
| MCPNC14 | 500.0 | 100 | 1.0 | 10 | 99.7 | 146.9 |
| MCPNC15 | 500.0 | 100 | 1.0 | 5.0 | 99.7 | 296.3 |
| MCPNC16 | 500.0 | 100 | 1.0 | 10 | 75.0 | 645.9 |
| MCPNC17 | 500.0 | 100 | 1.0 | 10 | 50.0 | 892.8 |

^a^ The concentration of the feeding PVP under reaction.

^b^ The volume of the feeding CaCl_2_ is 1.0 mL.

^c^ The concentration of the feeding NaOH or H_2_O_2_ solutions before reaction.

^d^ The volume of the feeding ethanol is 15 mL.

^e^ Average particle size of the MCPNC measured by TEM.**Table S2.** Synthesis conditions and characterization results of MCPNC14-FH1-5.

| Sample | MCPNC14  (mL) ^a^ | FH  (mL) ^b^ | Pure Water (mL) | FH/Ca  Mass Ratio ^c^ | FH Loading Efficiency (%) ^d^ | FH Loading Content (%) ^e^ |
| --- | --- | --- | --- | --- | --- | --- |
| MCPNC14-FH1 | 1.0 | 0.32 | 3.68 | 1.6 | 43.6 | 28.4 |
| MCPNC14-FH2 | 1.0 | 0.16 | 3.84 | 0.8 | 60.6 | 22.3 |
| MCPNC14-FH3 | 1.0 | 0.08 | 3.92 | 0.4 | 75.1 | 10.8 |
| MCPNC14-FH4 | 1.0 | 0.04 | 3.96 | 0.2 | 83.3 | 6.36 |
| MCPNC14-FH5 | 1.0 | 0.02 | 3.98 | 0.1 | 86.7 | 2.76 |

^a^ The Ca concentration (*C*_Ca_) of feeding MCPNC14 dispersed in pure water is 25 mM.

^b^ The concentration of feeding FH dissolved in H_2_O is 2.0 mg/mL.

^c^ Calculated from the mass ratio of feeding FH to Ca in MCPNC14.

^d^ Calculated from the mass percentage of loaded FH to the feeding FH.

^e^ Calculated from the mass percentage of loaded FH to the MCPNC14.**Table S3.** Synthesis conditions and characterization results of MCPNC14-FH2-IO1-5.

| Sample | MCPNC14-FH2  (mL) ^a^ | IO ^b^  (mL) | Pure Water (mL) | Fe/Ca  Mass Ratio ^c^ | IO Loading Efficiency (%) ^d^ | IO Loading Content (%) ^e^ |
| --- | --- | --- | --- | --- | --- | --- |
| MCPNC14-FH2-IO1 | 1.0 | 0.58 | 3.42 | 1.6 | 30.6 | 24.6 |
| MCPNC14-FH2-IO2 | 1.0 | 0.29 | 3.71 | 0.8 | 46.9 | 19.7 |
| MCPNC14-FH2-IO3 | 1.0 | 0.15 | 3.85 | 0.4 | 66.3 | 17.3 |
| MCPNC14-FH2-IO4 | 1.0 | 0.07 | 3.93 | 0.2 | 78.6 | 9.6 |
| MCPNC14-FH2-IO5 | 1.0 | 0.04 | 3.96 | 0.1 | 85.3 | 4.1 |

^a^ The Ca concentration (*C*_Ca_) of feeding MCPNC14-FH2 dispersed in pure water is 25 mM.

^b^ The Fe concentration (*C*_Fe_) of feeding IO dispersed in H_2_O is 20 mM.

^c^ Calculated from the mass ratio of feeding Fe in IO to Ca in MCPNC14-FH2.

^d^ Calculated from the mass percentage of loaded IO to the feeding IO.

^e^ Calculated from the mass percentage of loaded IO to the MCPNC14.

**Table S4.** Synthesis conditions and characterization results of MCPNC14-FH2-IO3@HA1-6.

| Sample | MCPNC14-FH2-IO3  (mL) ^a^ | HA  (mL) ^c^ | Pure Water  (mL) | HA/Ca  Mass Ratio ^d^ | *r*_1_  (mM^-1^ s^-1^) ^e^ | *r*_2_  (mM^-1^ s^-1^) ^e^ | *r*_2_/*r*_1_ |
| --- | --- | --- | --- | --- | --- | --- | --- |
| MCPNC14-FH2-IO3@HA1 | 1.0 | 0.50 | 3.50 | 1.0 | 8.09 | 166.8 | 20.6 |
| MCPNC14-FH2-IO3@HA2 | 1.0 | 0.40 | 3.60 | 0.8 | 8.68 | 144.8 | 16.7 |
| MCPNC14-FH2-IO3@HA3 | 1.0 | 0.30 | 3.70 | 0.6 | 9.46 | 131.4 | 13.9 |
| MCPNC14-FH2-IO3@HA4 | 1.0 | 0.20 | 3.80 | 0.4 | 10.3 ± 0.7 | 123.5 ± 5.1 | 11.9 ± 1.3 |
| MCPNC14-FH2-IO3@HA5 | 1.0 | 0.10 | 3.90 | 0.2 | 8.54 | 87.42 | 10.2 |
| MCPNC14-FH2-IO3@HA6 | 1.0 | 0.05 | 3.95 | 0.1 | 9.19 | 85.29 | 9.28 |
| Magnevist^®^ | - | - | - | - | 4.23 ± 0.07 | 4.13 ± 0.09 | 0.98 ± 0.04 |
| IO | - | - | - | - | 9.74 ± 0.19 | 43.7 ± 1.2 | 4.48 ± 0.21 |

^a^ The Ca concentration (*C*_Ca_) of feeding MCPNC14-FH2-IO3 dispersed in H_2_O is 25 mM.

^c^ The concentration of feeding HA dissolved in pure water is 2.0 mg/mL.

^d^ Calculated from the mass ratio of feeding HA to Ca in MCPNC14-FH2-IO3.

^e^ The *r*_1_ and *r*_2_ were measured by a clinical MRI scanner system (3.0 T, Ingenia, Philips, Netherlands). Mean ± S.D., *n* = 3.


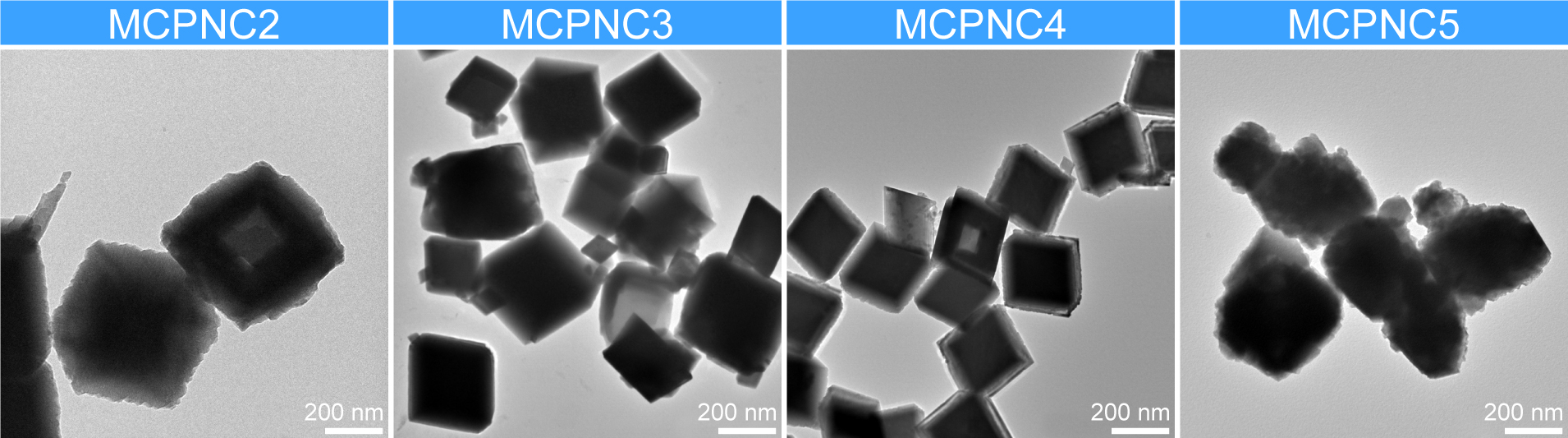


**Figure S1.** TEM images for MCPNC2-5.


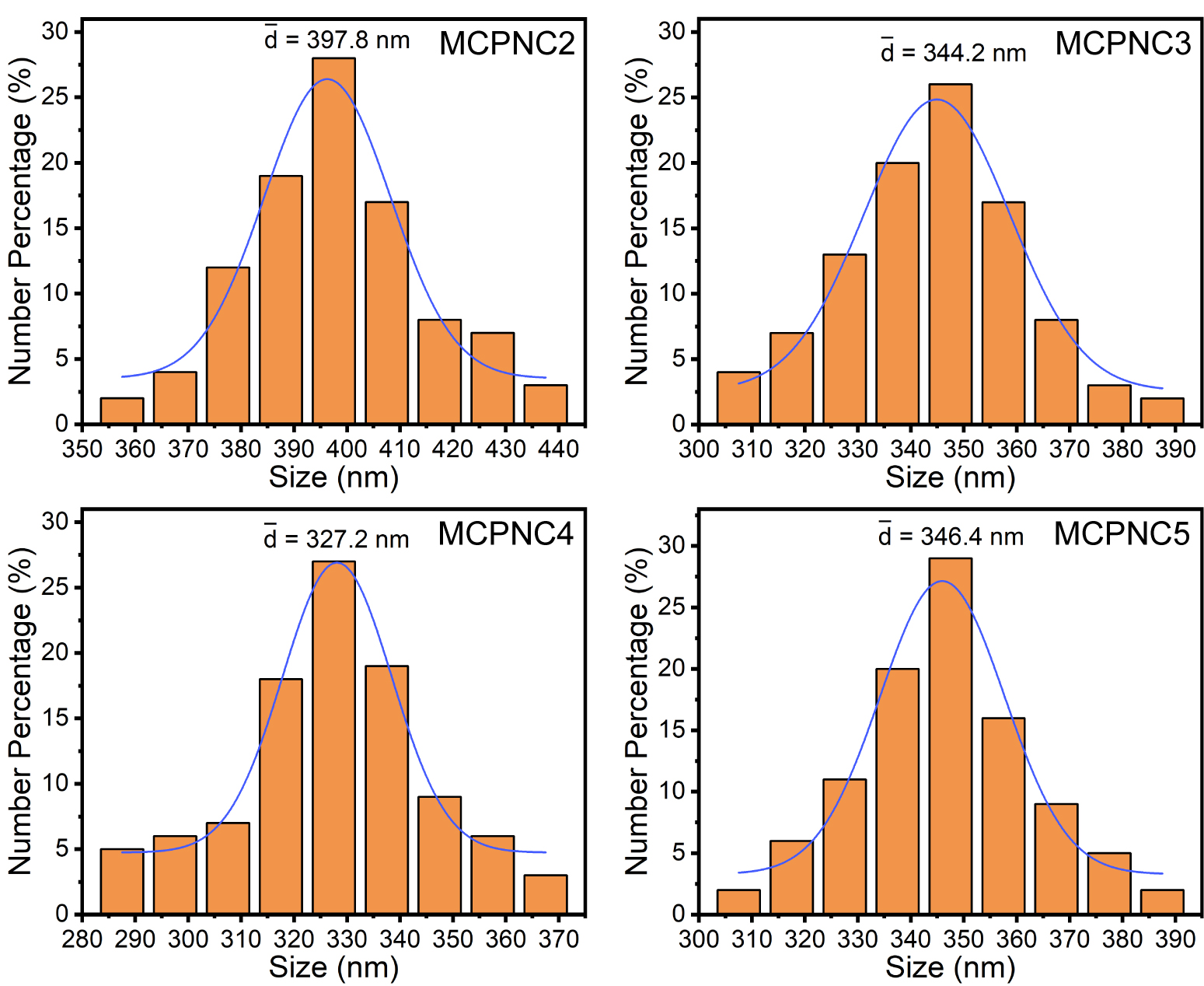


**Figure S2.** The particle size distributions of MCPNC2-5 measured by TEM.


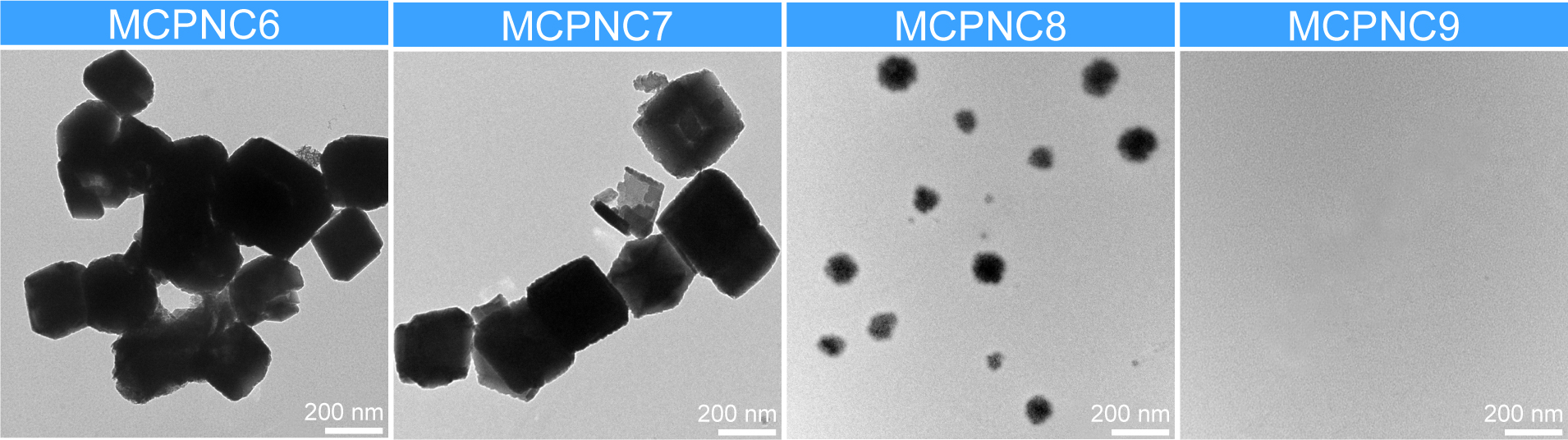


**Figure S3.** TEM images for MCPNC6-9.


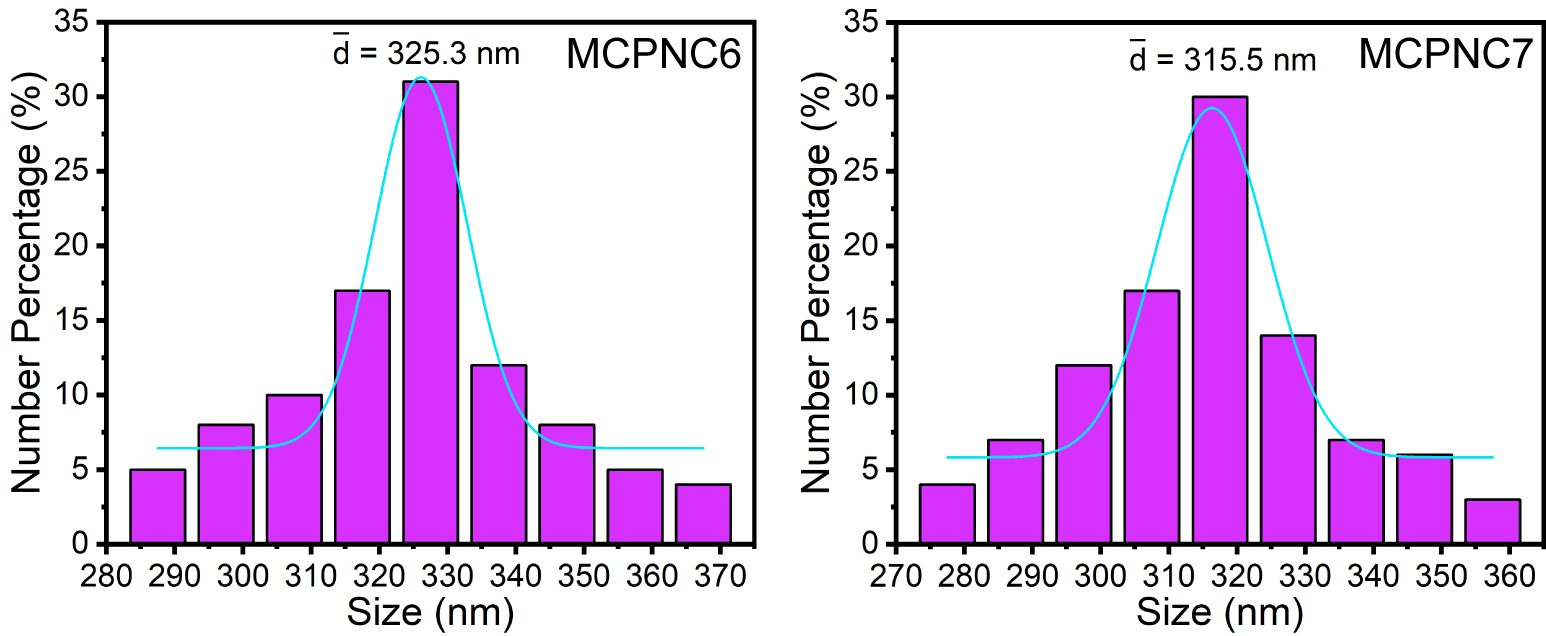


**Figure S4.** The particle size distributions of MCPNC6 and MCPNC7 measured by TEM.


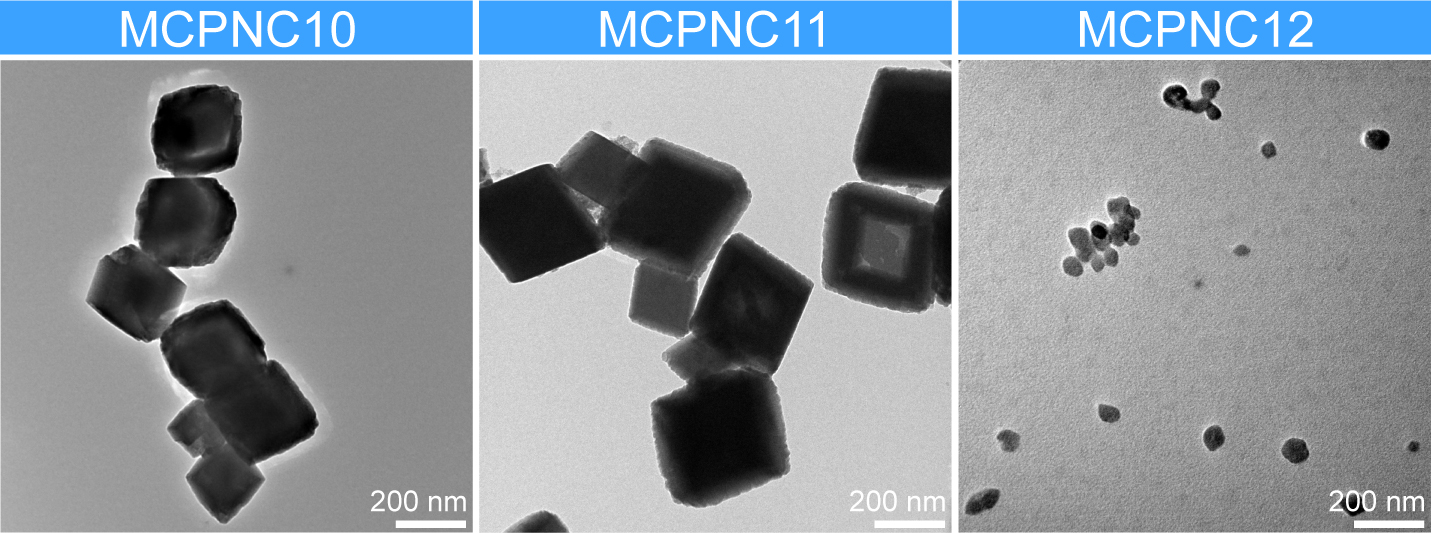


**Figure S5.** TEM images for MCPNC10-12.


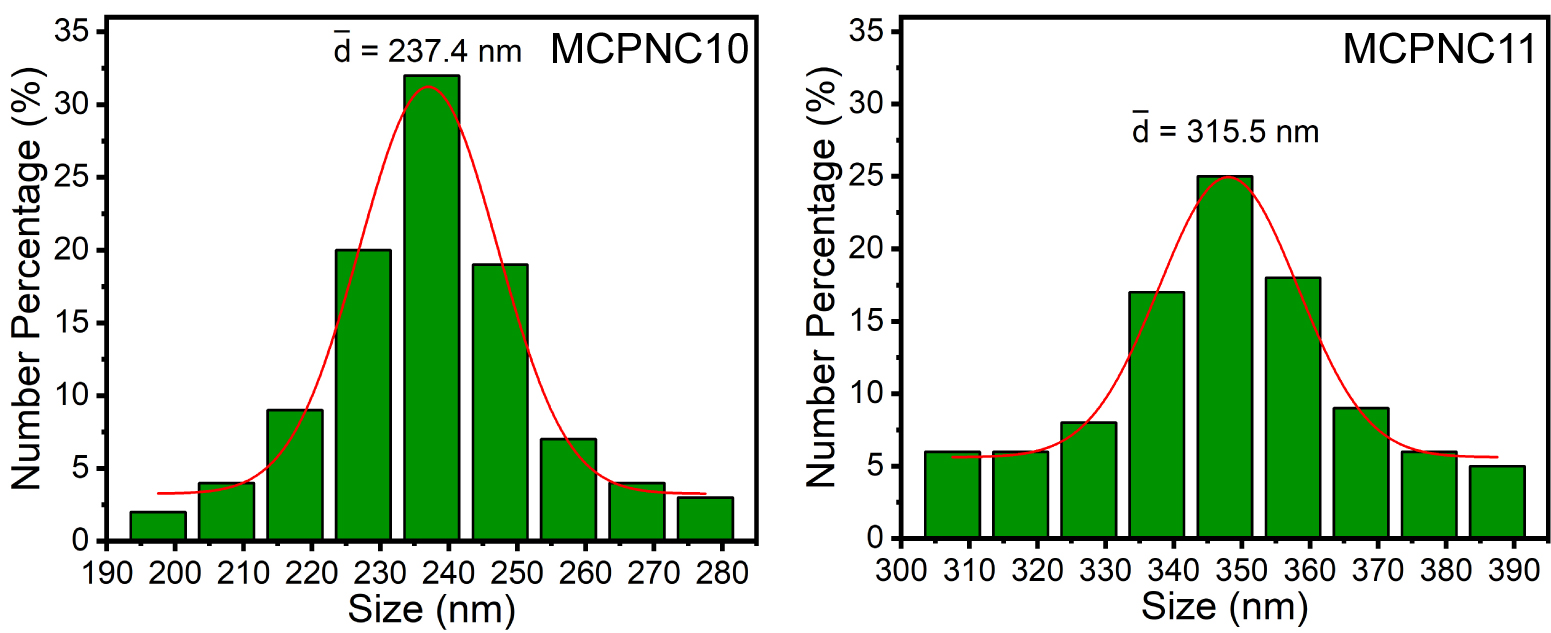


**Figure S6.** The particle size distributions of MCPNC10 and MCPNC11 measured by TEM.


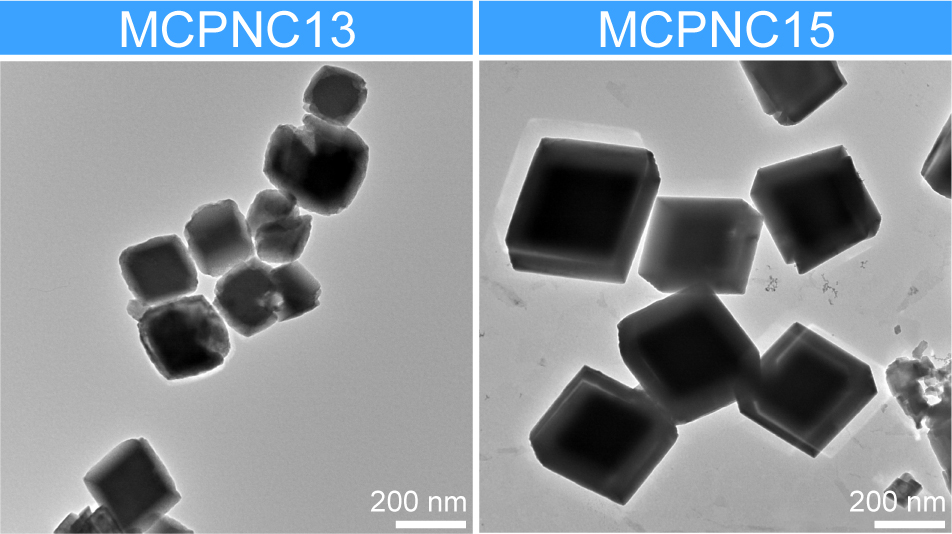


**Figure S7.** TEM images for MCPNC13 and MCPNC15.


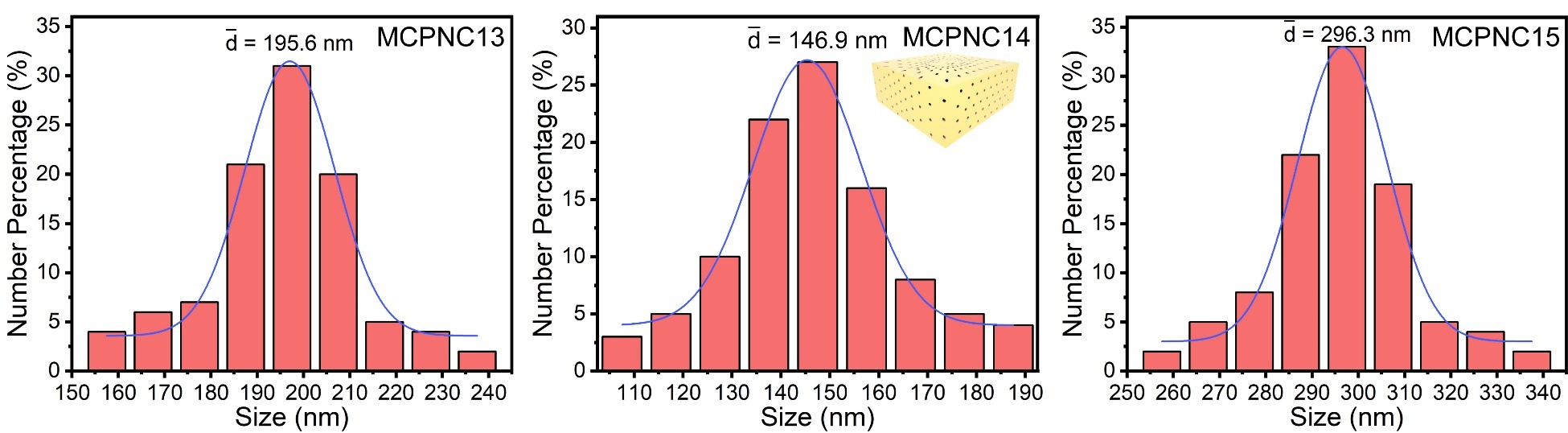


**Figure S8.** The particle size distributions of MCPNC13-15 measured by TEM.


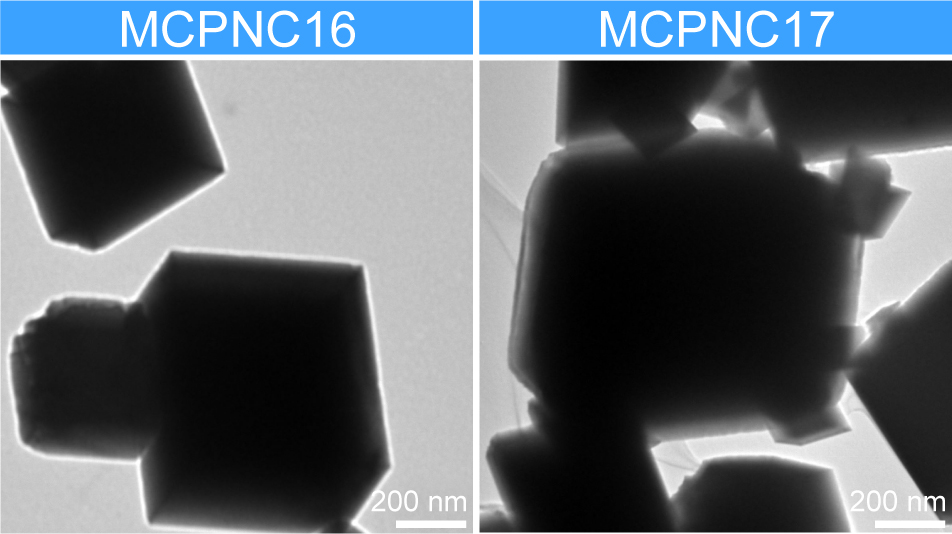


**Figure S9.** TEM images for MCPNC16 and MCPNC17.


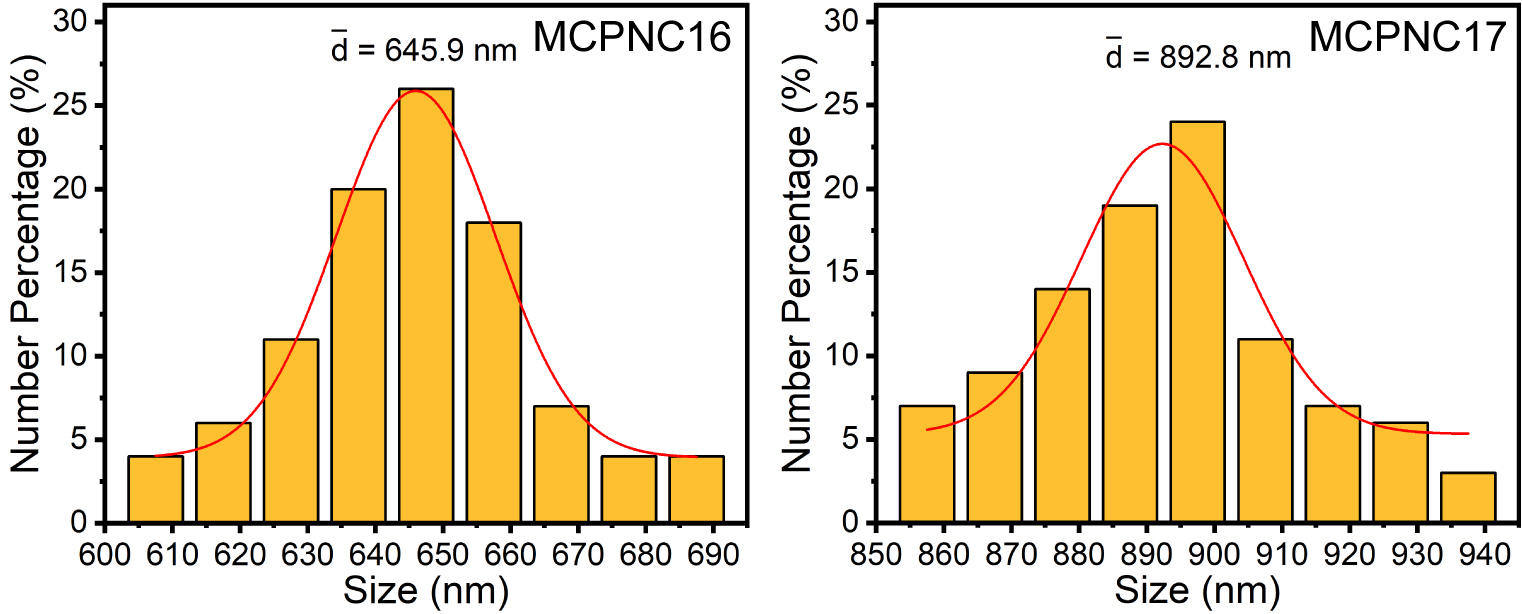


**Figure S10.** The particle size distributions of MCPNC16 and MCPNC17 measured by TEM.


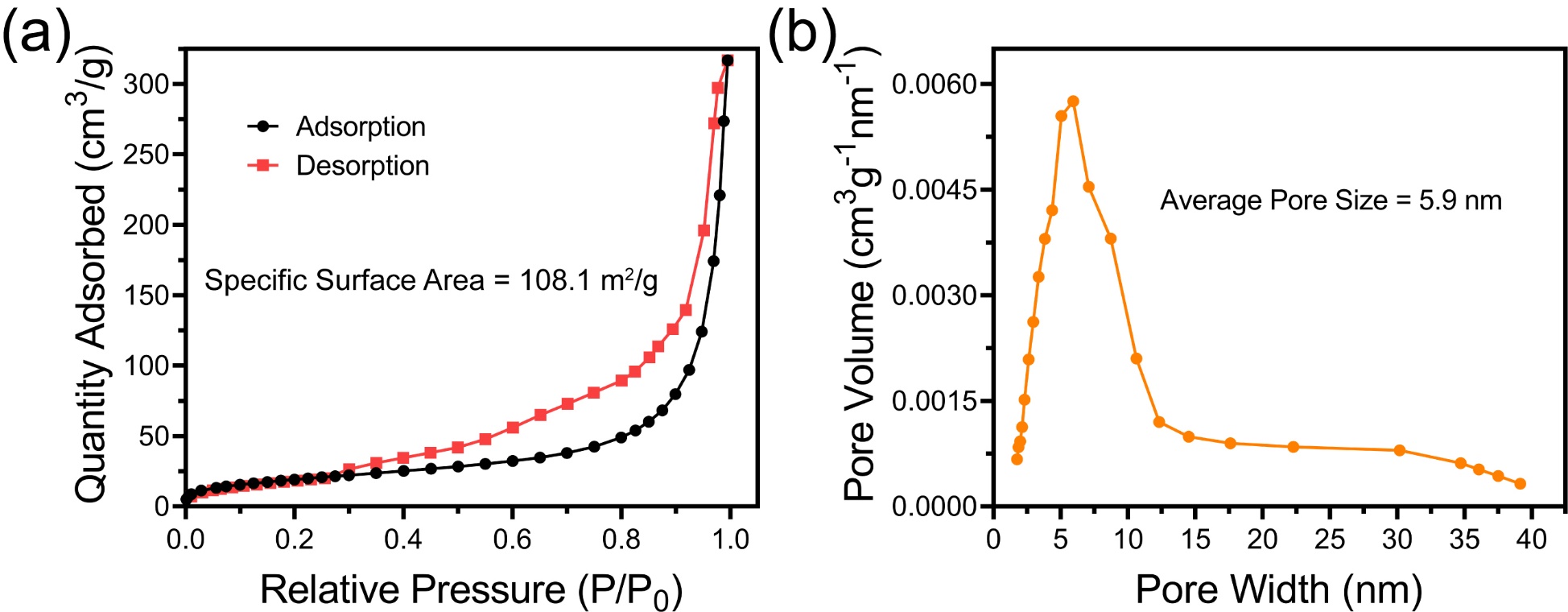


**Figure S11.** N_2_ adsorption-desorption isotherms (a), and pore size distributions (b) of the MCPNC14.


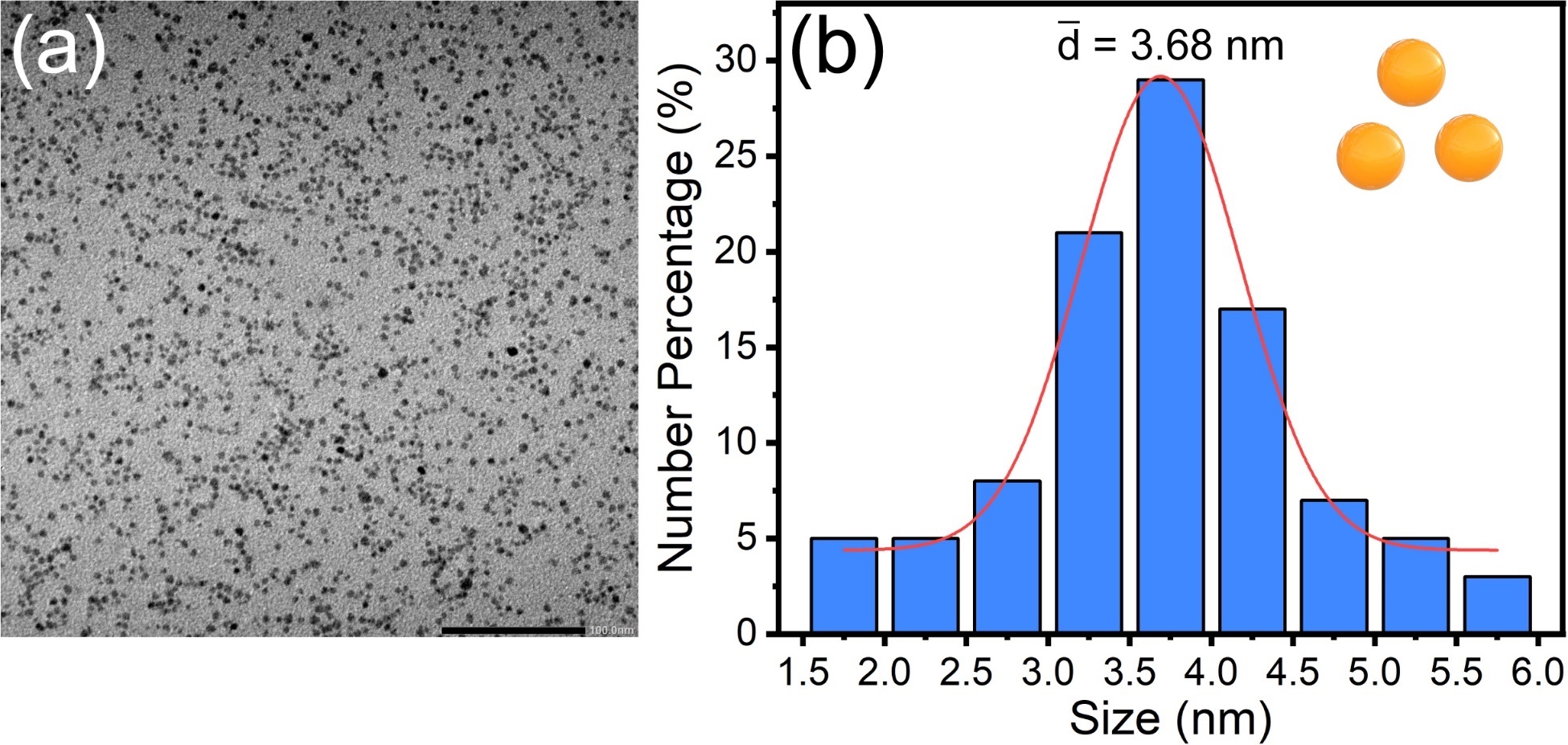


**Figure S12.** The TEM image (a), and the corresponding particle size distributions (b) of IO. Scale bar = 100 nm.


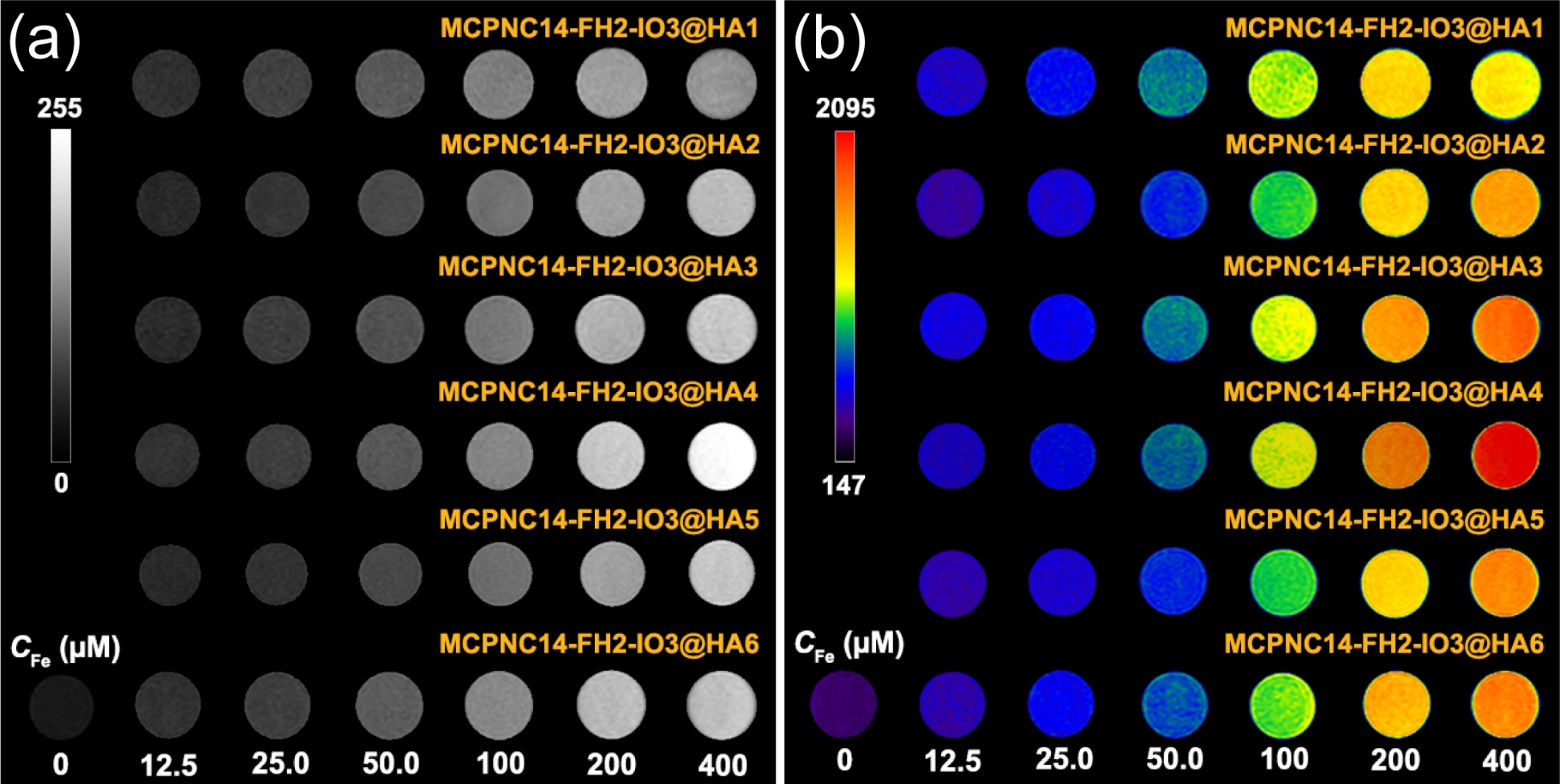


**Figure S13.** *T*_1_-weighted MR images (a), and their corresponding pseudo-color images (b) of MCPNC14-FH2-IO3@HA1-6 dispersions with different concentration of Fe. The magnetic field is 3.0 T. For *T*_1_ relaxation rates: TE = 8.4 ms, TR = 500 ms.


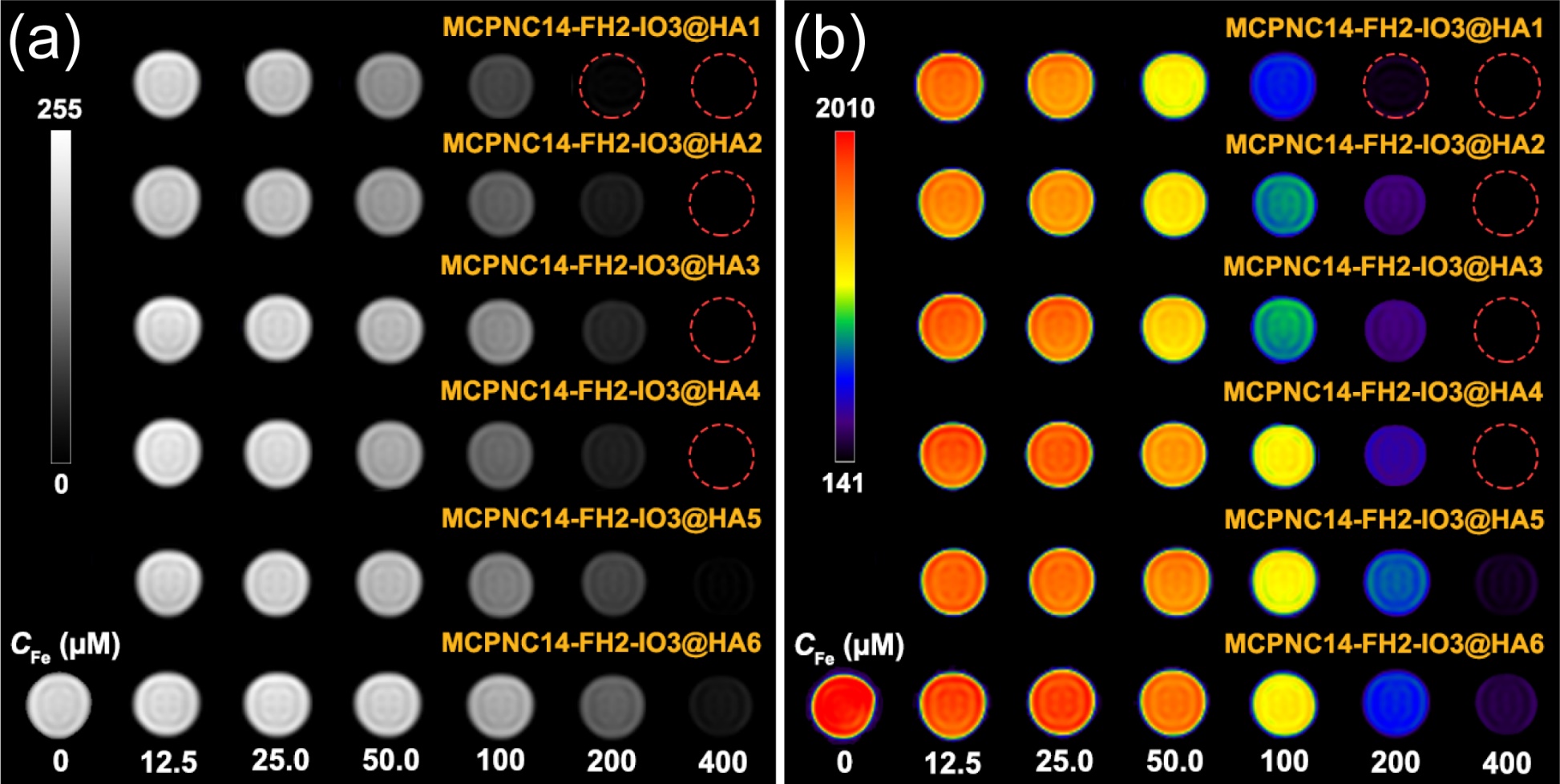


**Figure S14.** *T*_2_-weighted MR images (a), and their corresponding pseudo-color images (b) of MCPNC14-FH2-IO3@HA1-6 dispersions with different concentration of Fe. The magnetic field is 3.0 T. For *T*_2_ relaxation rates: TE = 80.0 ms, TR = 5000 ms.


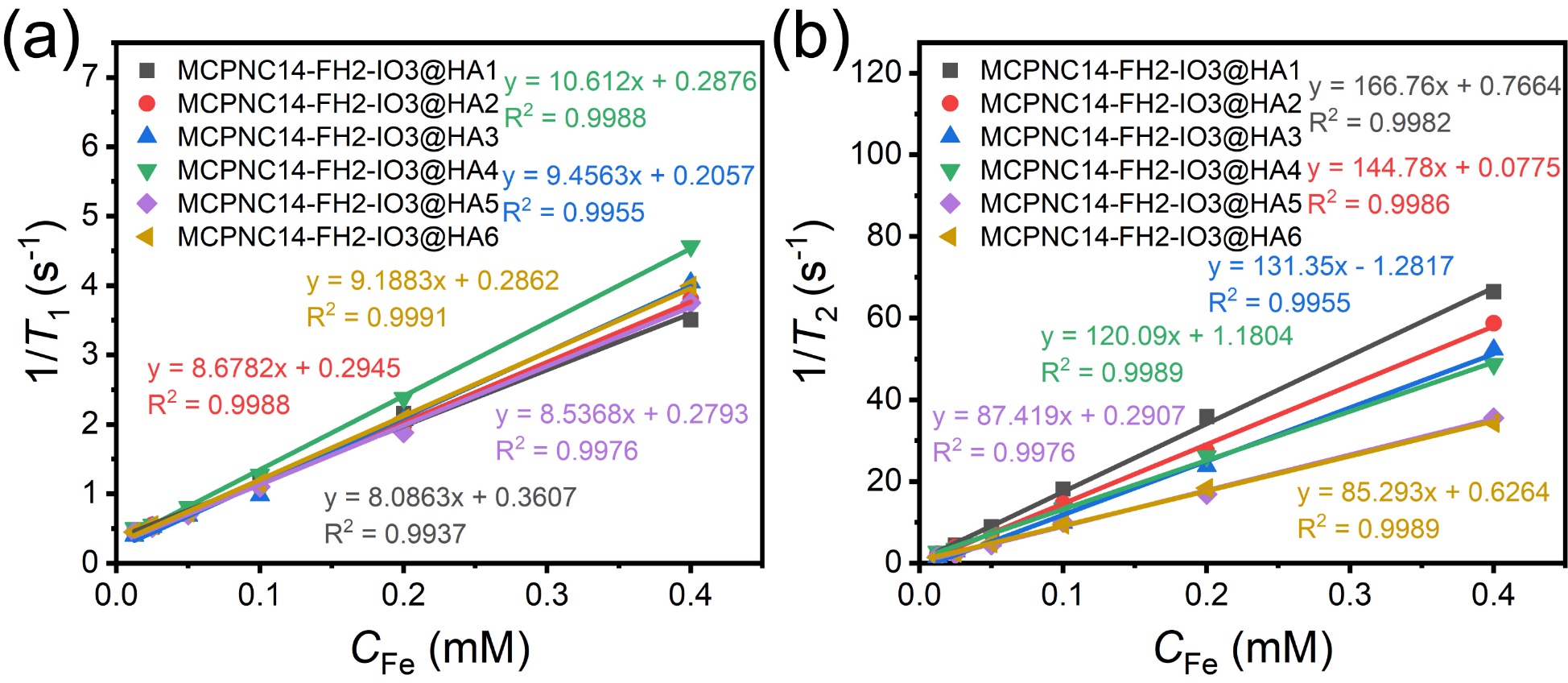


**Figure S15.** *T*_1_ relaxation rate (1/*T*_1_) (a) and *T*_2_ relaxation rate (1/*T*_2_) (b) plotted as a function of Fe concentration for MCPNC14-FH2-IO3@HA1-6 dispersions.


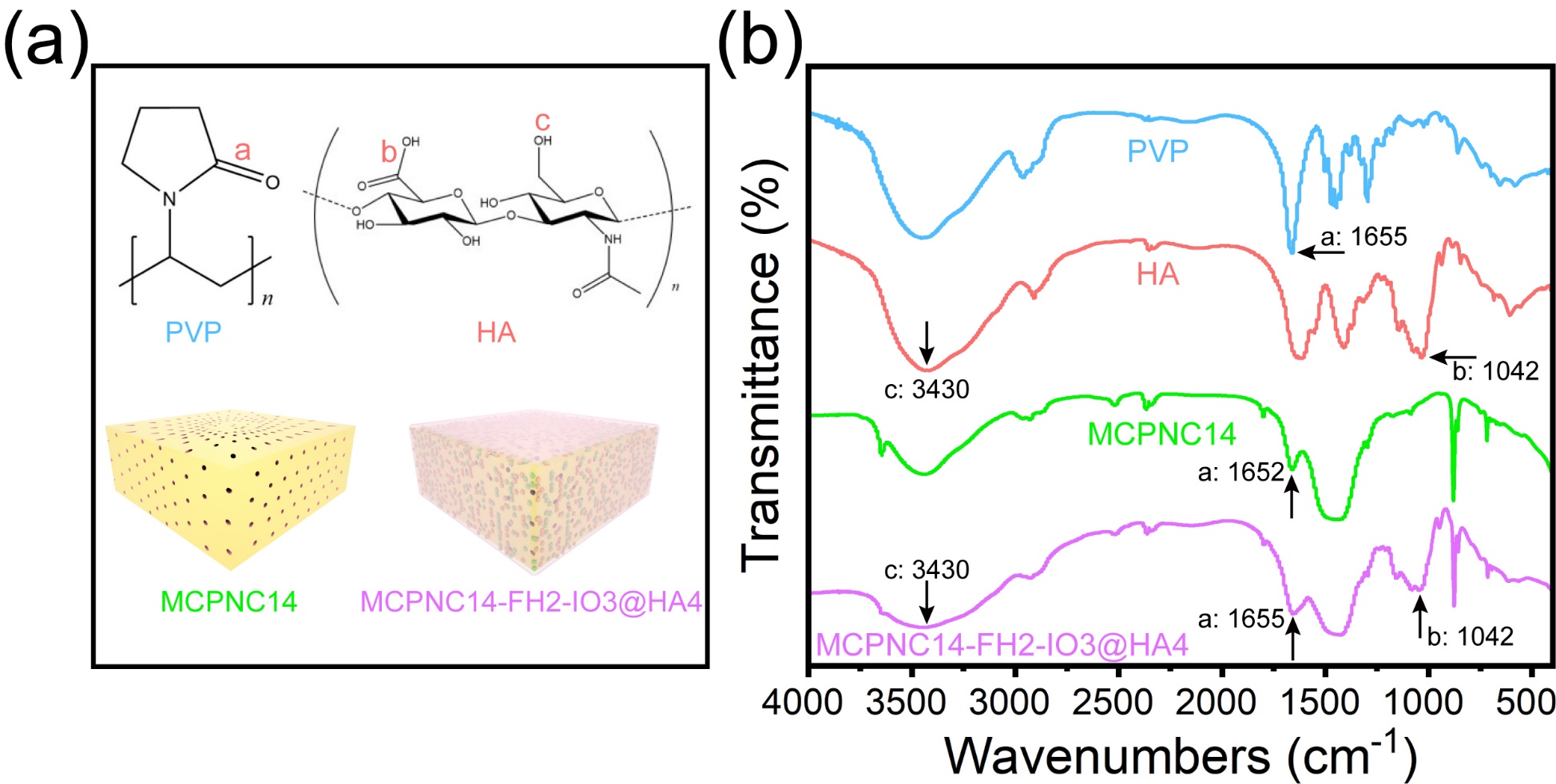


**Figure S16.** (a): Molecular structures of PVP and HA, and schematic drawing of MCPNC14 and MCPNC14-FH2-IO3@HA4. (b): FT-IR spectra of PVP, HA, MCPNC14, and MCPNC14-FH2-IO3@HA4.

**
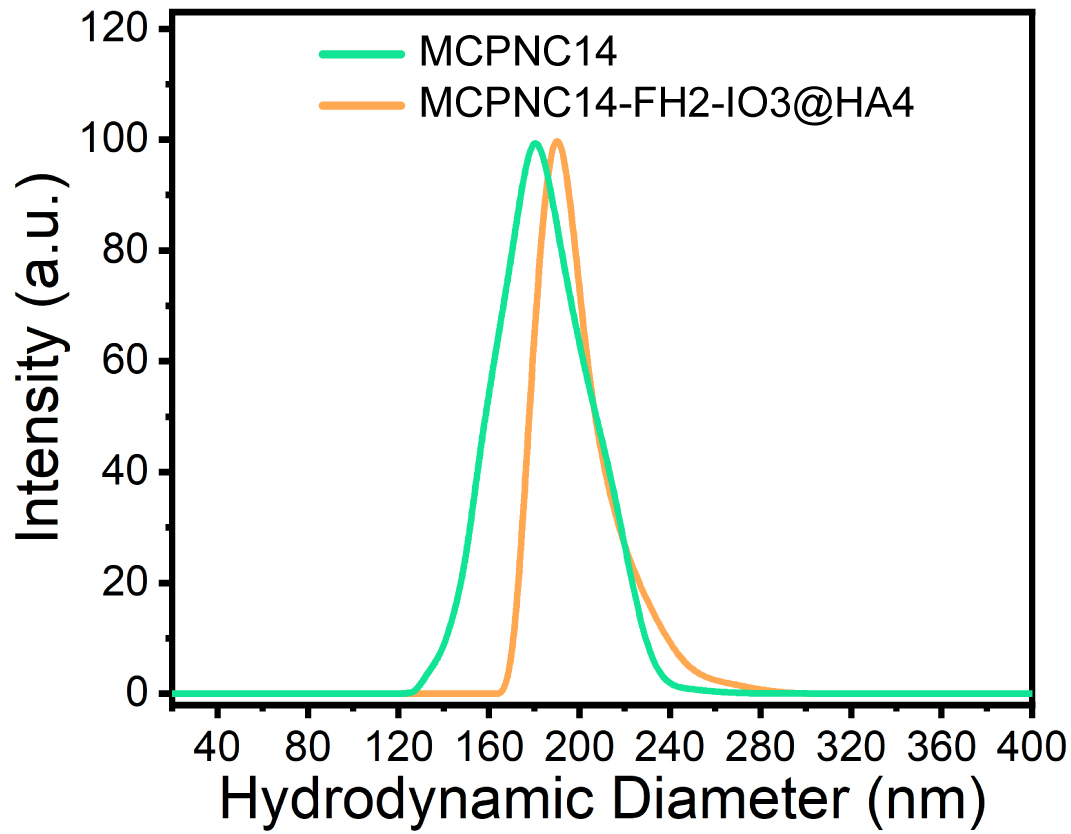
**

**Figure S17.** Size distributions of MCPNC14 and MCPNC14-FH2-IO3@HA4 measured by the dynamic light scattering (DLS).


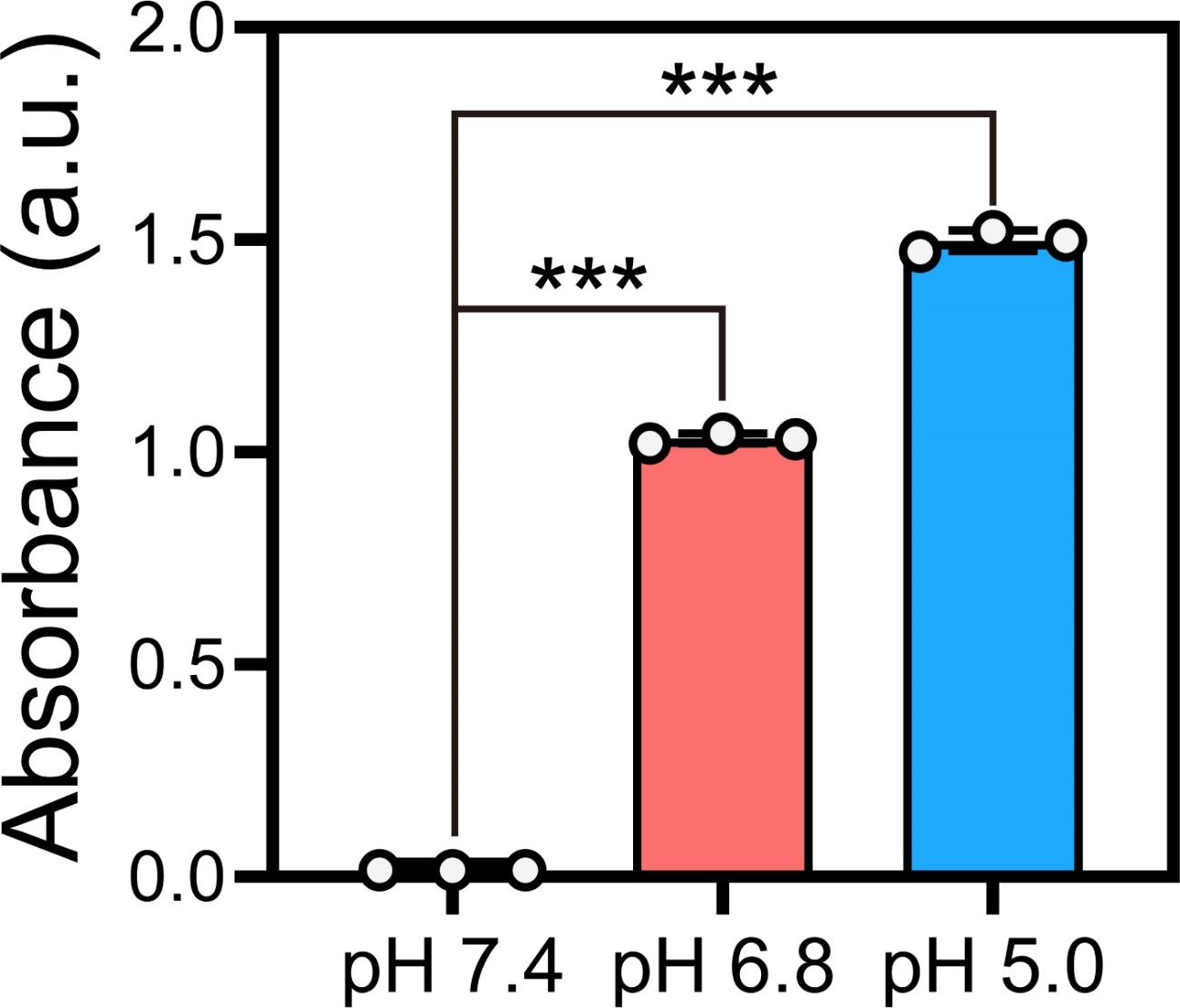


**Figure S18.** The corresponding quantitative analysis of the UV-vis absorption peak of TMB solutions at 652 nm incubated with MCPNC14-FH2-IO3@HA4 in PBS at pH 5.0, 6.8, or 7.4. Mean ± S.D., *n* = 3. ***P < 0.001.


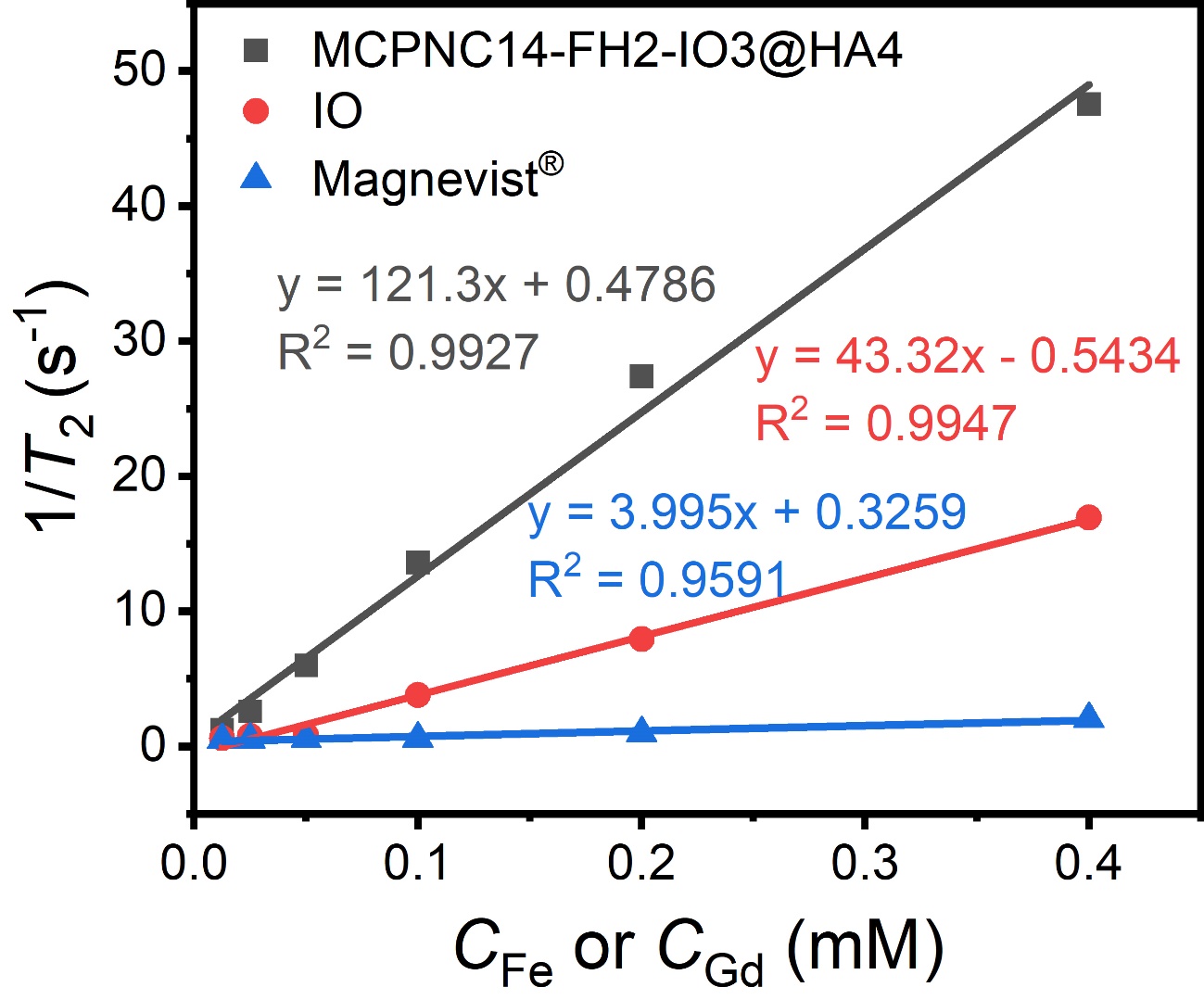


**Figure** **S19.** *T*_2_ relaxation rate (1/*T*_2_) plotted as a function of Fe or Gd concentration for MCPNC14-FH2-IO3@HA4, IO, or commercial Magnevist^®^ solutions. For *T*_2_ relaxation rates: TE = 80.0 ms, TR = 5000 ms. Magnetic field = 3.0 T.


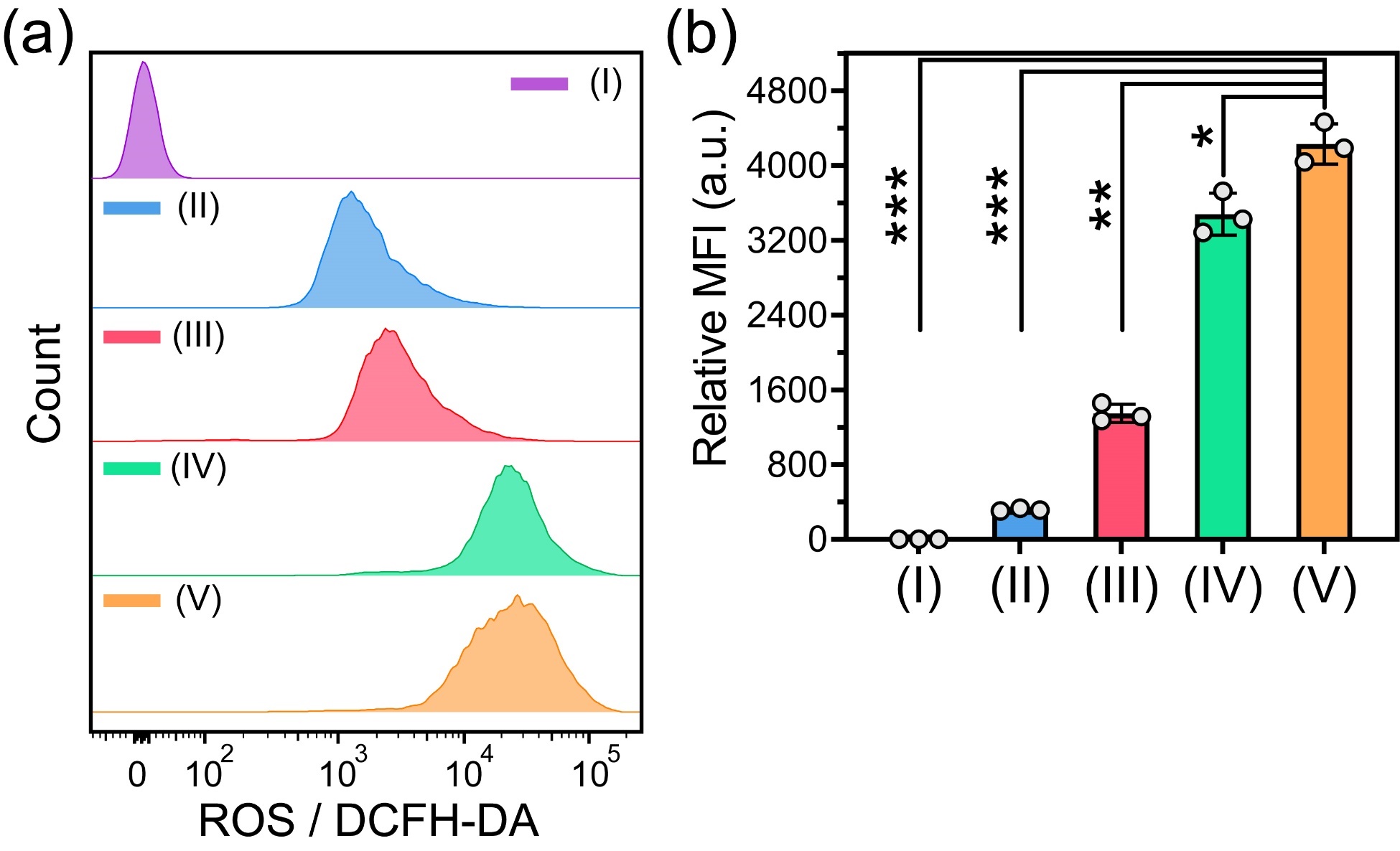


**Figure S20.** DCF fluorescence distributions (a) and the corresponding quantitative analysis (b) of DCF fluorescence intensity for 4T1 cells after treatments with PBS (group Ⅰ), MCPNC14 (group Ⅱ), MCPNC14-FH2 (group Ⅲ), MCPNC14-FH2-IO3 (group Ⅳ), or MCPNC14-FH2-IO3@HA4 (group Ⅴ) determined by flow cytometry for the intracellular ROS generation assay. Mean ± S.D., *n* = 3. *P < 0.05, **P < 0.01, ***P < 0.001.


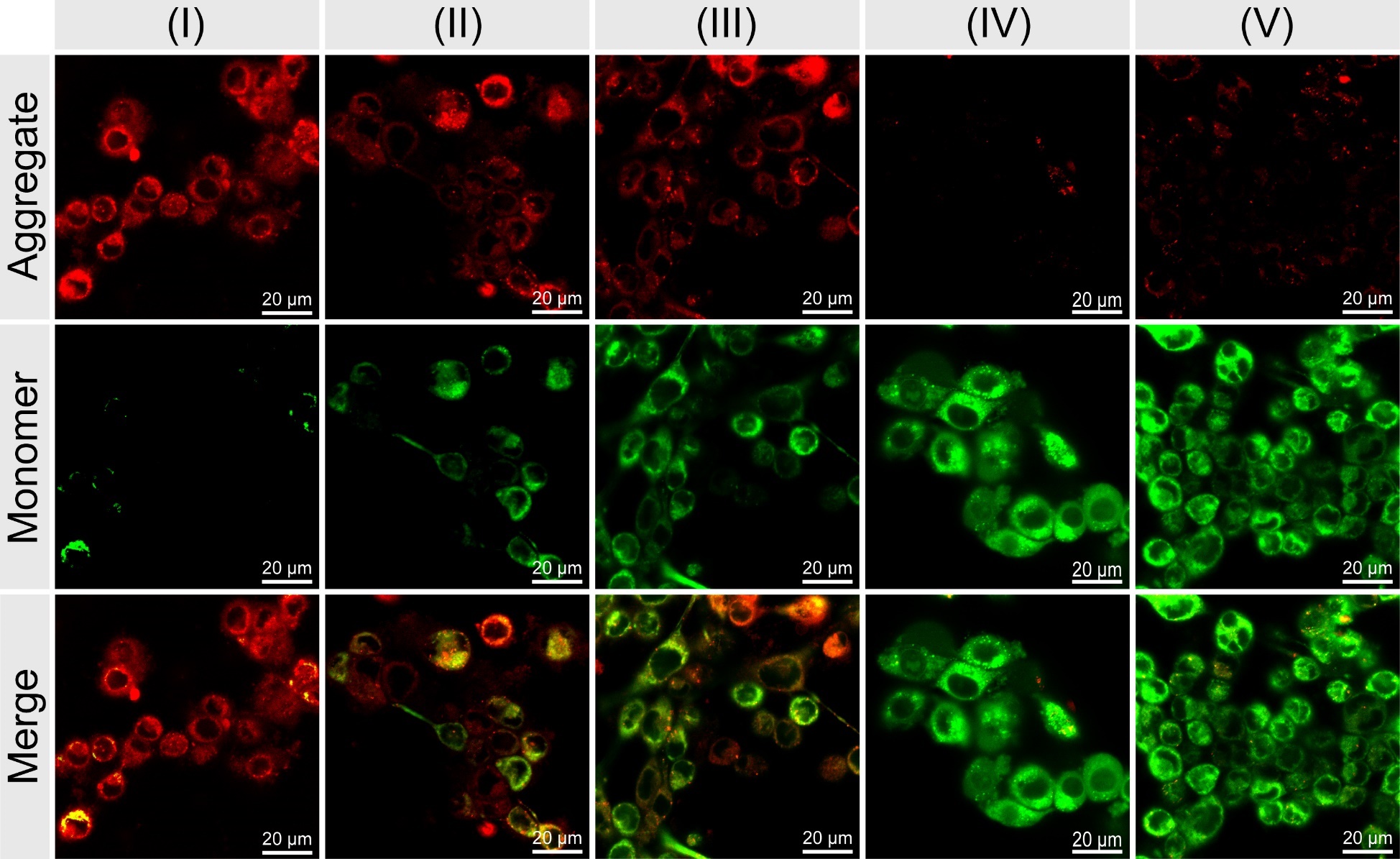


**Figure S21.** LSCM images of JC-1-stained 4T1 cells with treatments of PBS (group Ⅰ), MCPNC14 (group Ⅱ), MCPNC14-FH2 (group Ⅲ), MCPNC14-FH2-IO3 (group Ⅳ), or MCPNC14-FH2-IO3@HA4 (group Ⅴ). Red fluorescence: JC-1 aggregates for healthy mitochondrial with normal mitochondrial membrane potential. Green fluorescence: JC-1 monomers for damaged mitochondrial with declined mitochondrial membrane potential.


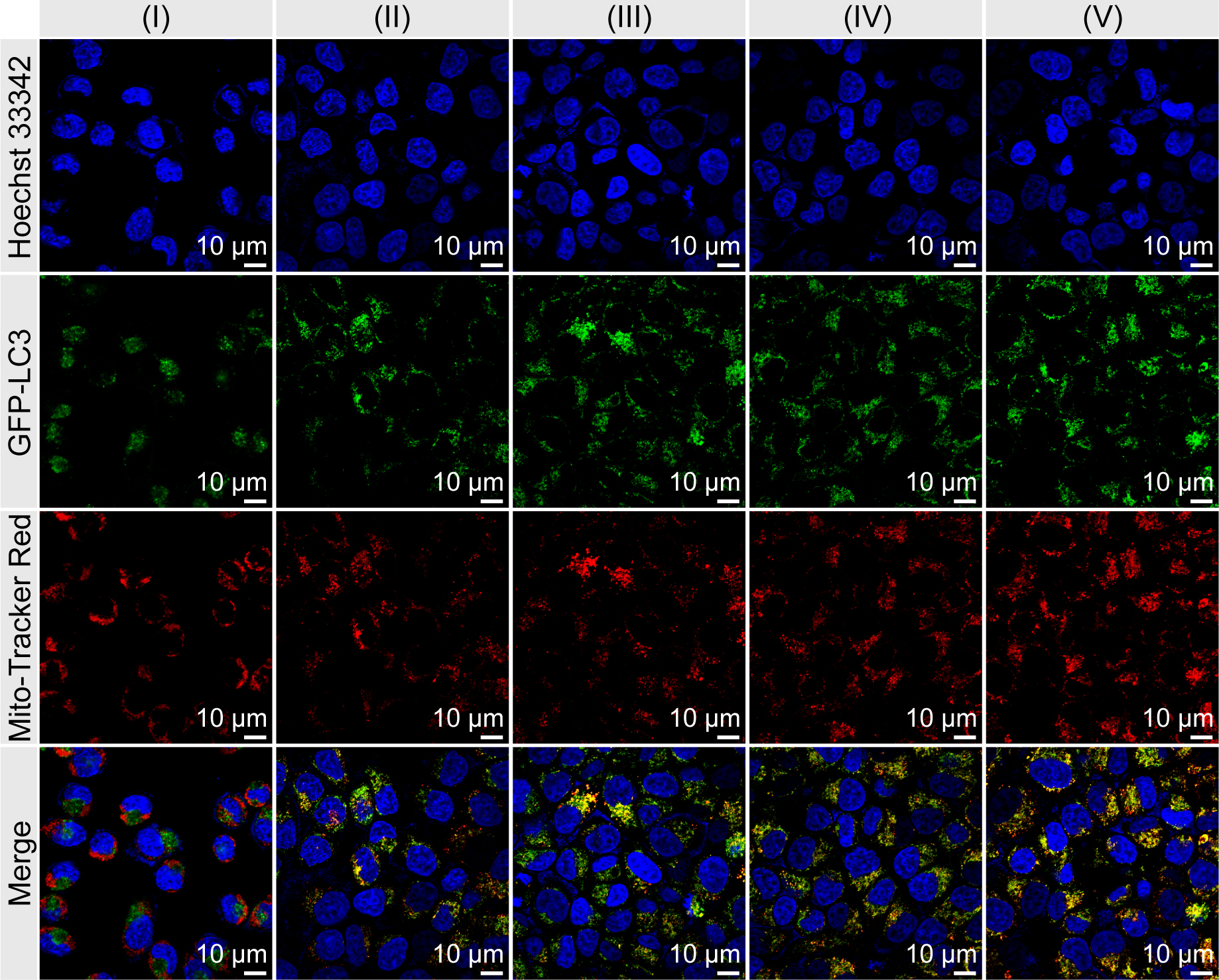


**Figure S22.** LSCM photos of 4T1 cells after incubation with PBS (group Ⅰ), MCPNC14 (group Ⅱ), MCPNC14-FH2 (group Ⅲ), MCPNC14-FH2-IO3 (group Ⅳ), or MCPNC14-FH2-IO3@HA4 (group Ⅴ), showing the co-localization of autophagy marker microtubule-associated protein 1 light chain 3 (LC3) with mitochondria. Green fluorescence: GFP-LC3 for LC3; red fluorescence: Mito-Tracker Red for mitochondria; blue fluorescence: Hoechst 33342 for nuclei.


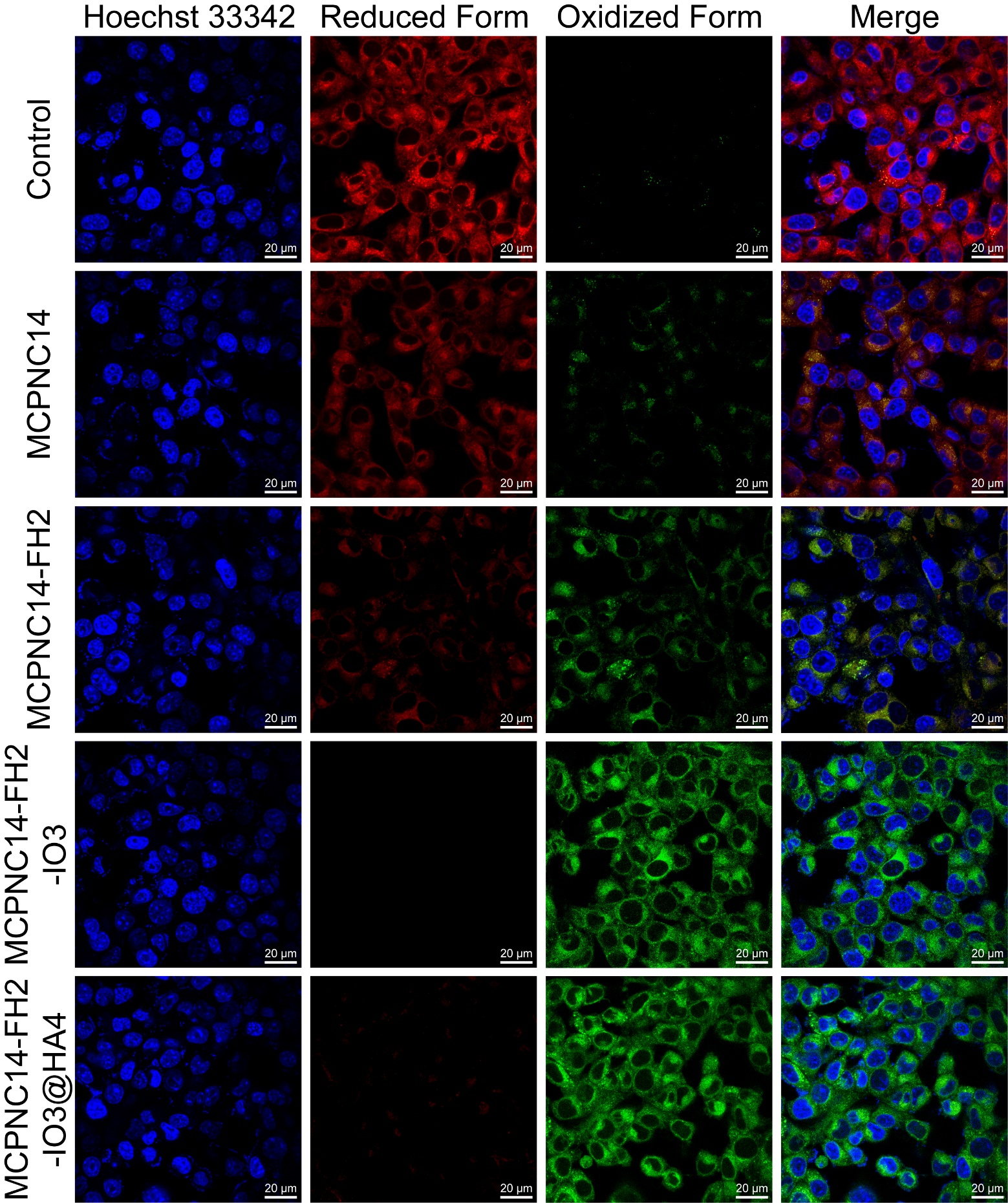


**Figure S23.** LSCM photos of the treated 4T1 cells after C11-BODIPY^581/591^ staining showing LPO generation. The increased green fluorescence and decreased red fluorescence indicate LPO generation. The nuclei stained with Hoechst 33342 are blue.


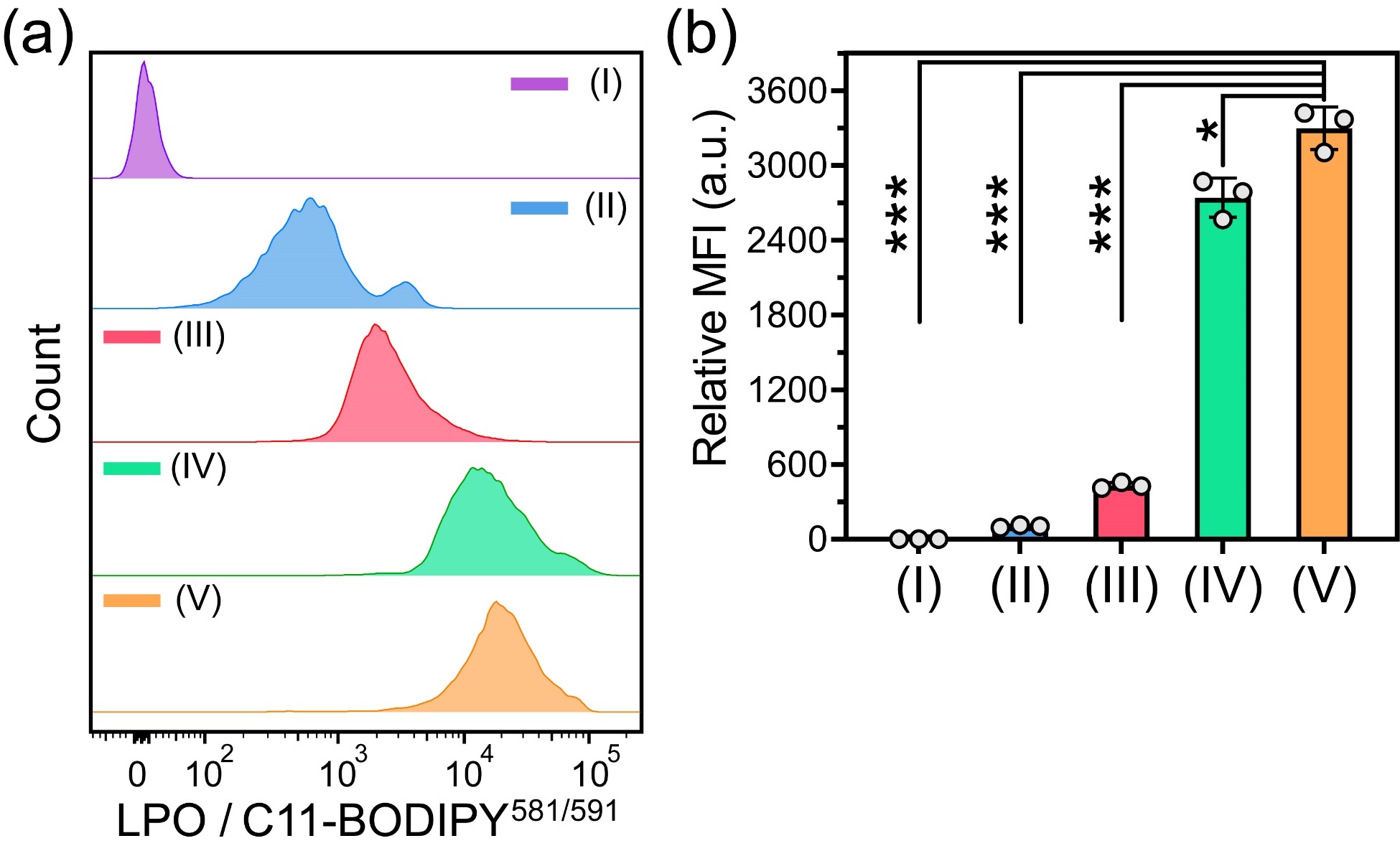


**Figure S24.** C11-BODIPY^581/591^ green fluorescence distributions (a), and the corresponding quantitative analysis (b) of 4T1 cells after treatment with PBS (group Ⅰ), MCPNC14 (group Ⅱ), MCPNC14-FH2 (group Ⅲ), MCPNC14-FH2-IO3 (group Ⅳ), or MCPNC14-FH2-IO3@HA4 (group Ⅴ) measured by flow cytometry for the intracellular LPO generation assay. Mean ± S.D., *n* = 3. *P < 0.05, ***P < 0.001.


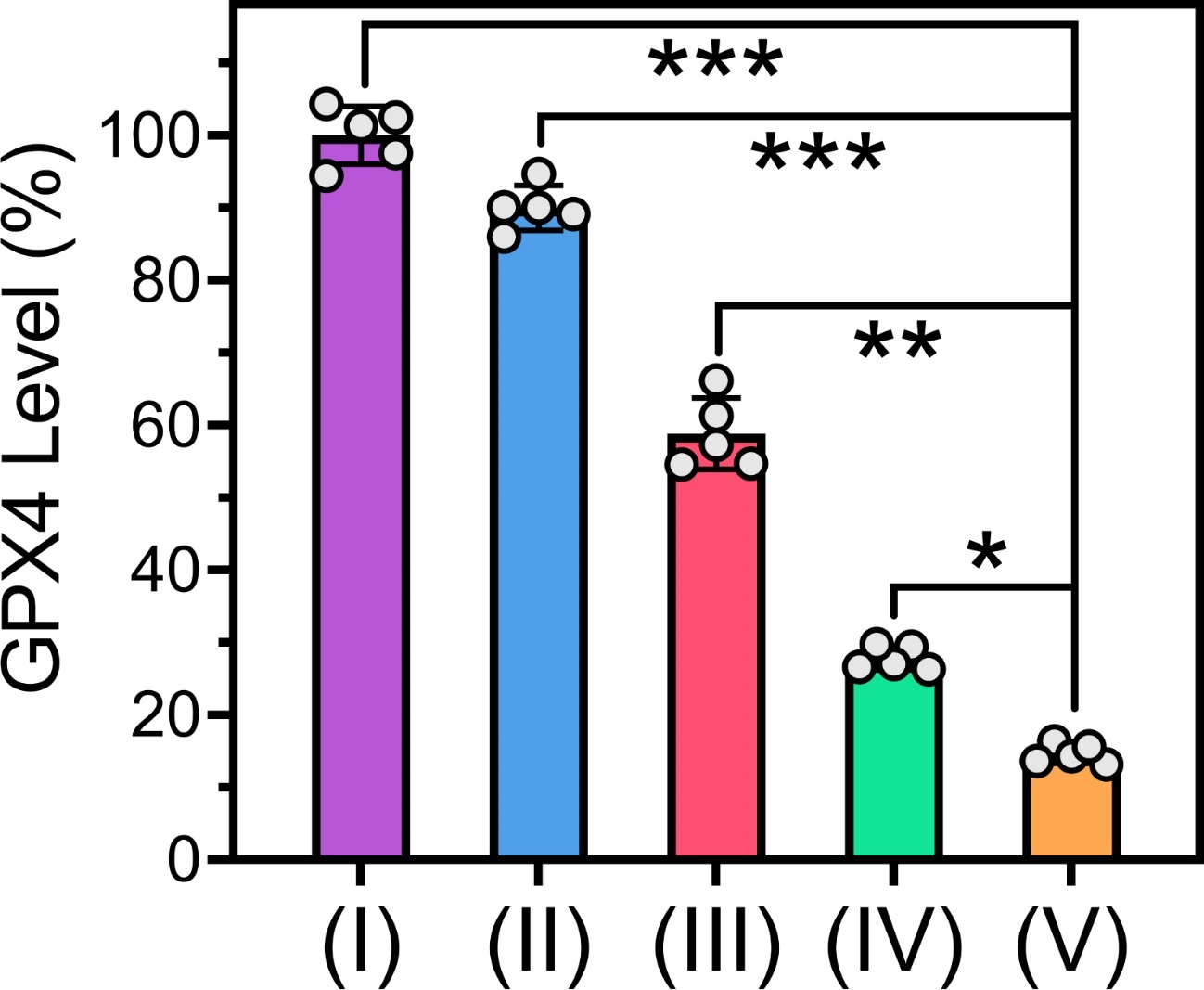


**Figure S25.** The relative intracellular GPX4 levels in 4T1 cells after treated with PBS (group Ⅰ), MCPNC14 (group Ⅱ), MCPNC14-FH2 (group Ⅲ), MCPNC14-FH2-IO3 (group Ⅳ), or MCPNC14-FH2-IO3@HA4 (group Ⅴ), measured by a GPX4 assay kit. Mean ± S.D., *n* = 5. *P < 0.05, **P < 0.01, ***P < 0.001.


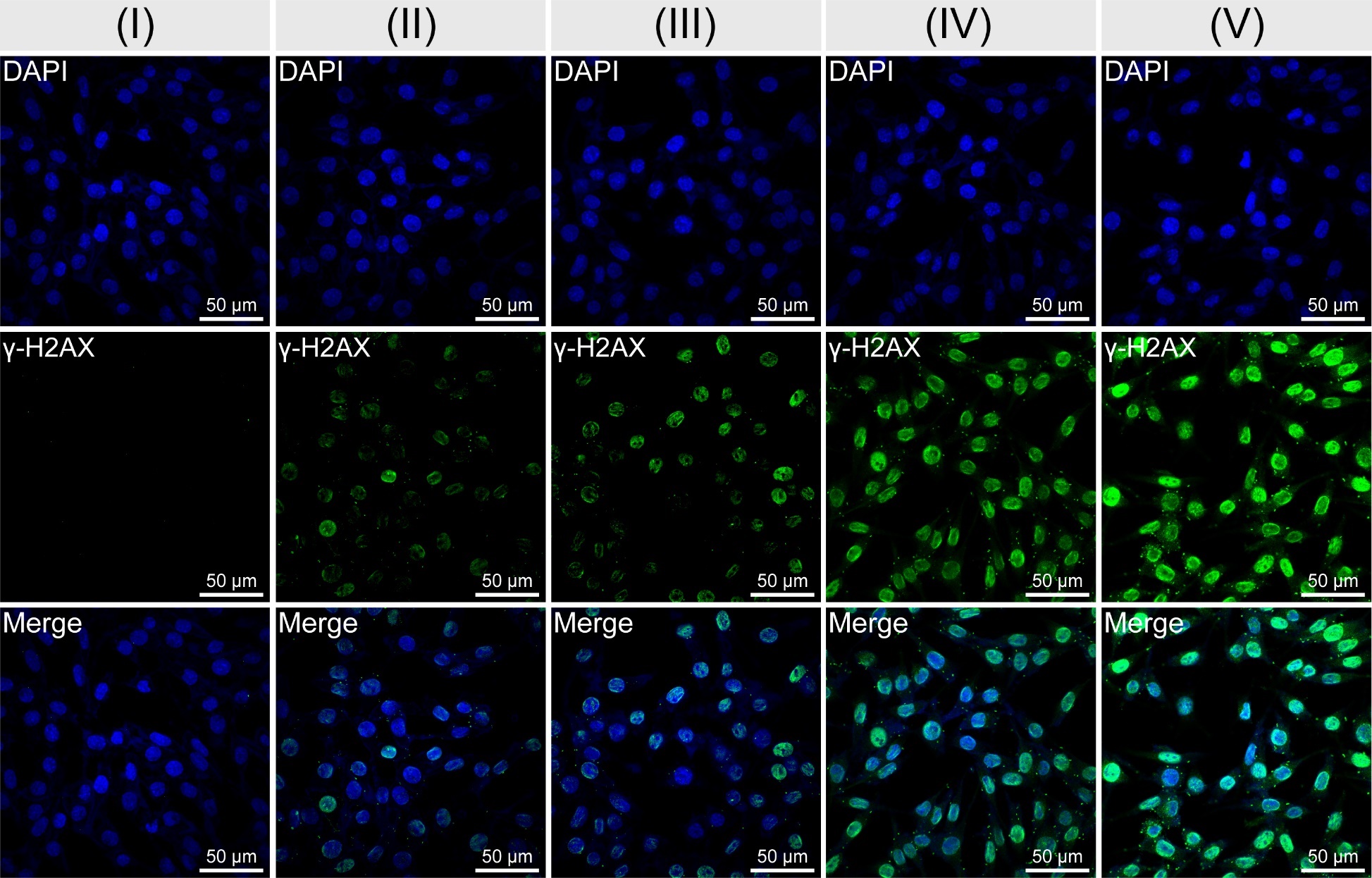


**Figure S26.** LSCM photos of 4T1 cells stained with fluorescence-labeled γ-H2AX second antibody after treatment with PBS (group Ⅰ), MCPNC14 (group Ⅱ), MCPNC14-FH2 (group Ⅲ), MCPNC14-FH2-IO3 (group Ⅳ), or MCPNC14-FH2-IO3@HA4 (group Ⅴ), showing the situation of intracellular damaged DNA. Green fluorescence: γ-H2AX for damaged DNA. Blue fluorescence: DAPI for nuclei.


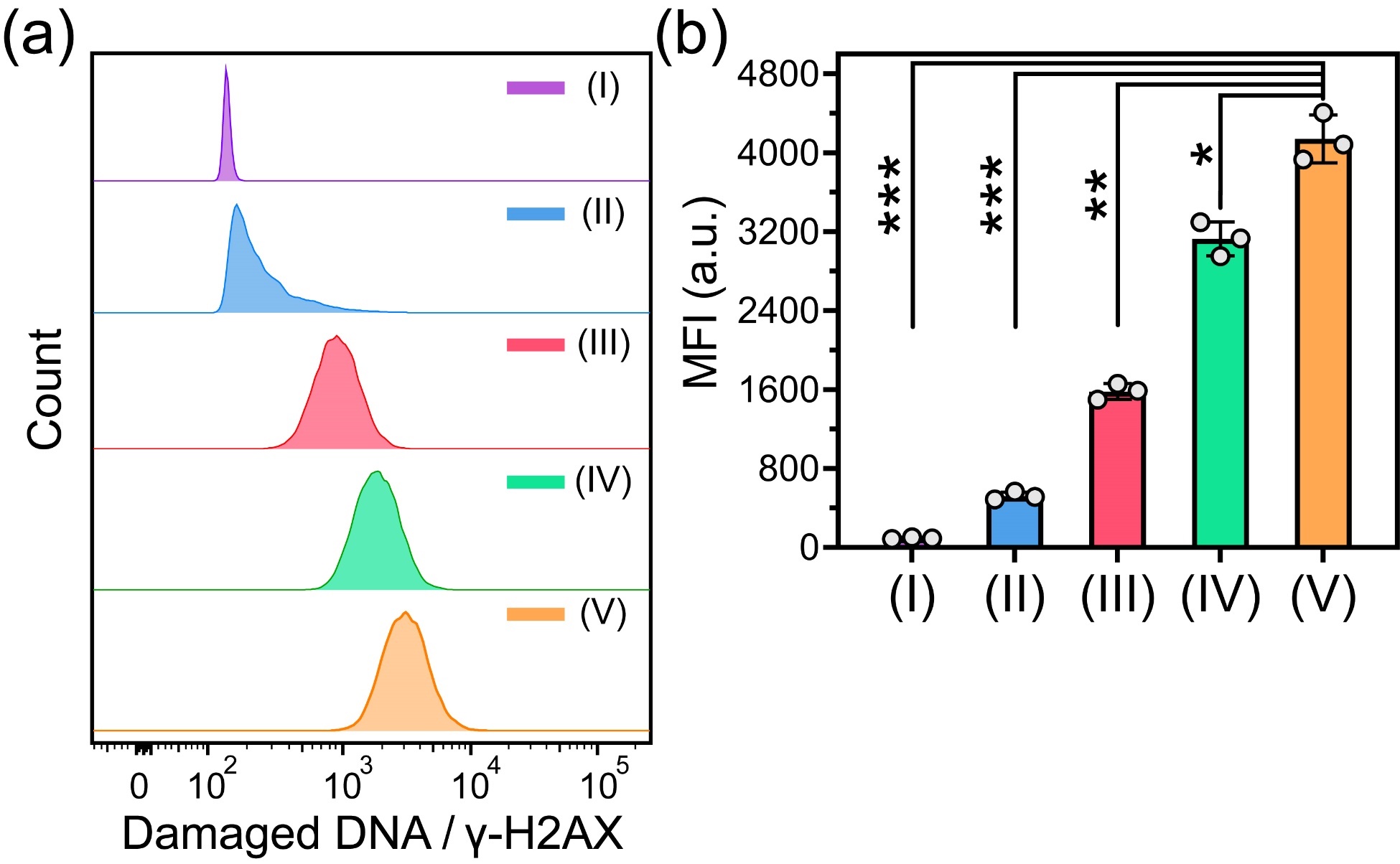


**Figure S27.** Flow cytometry fluorescence distributions (a), and the corresponding quantitative analysis (b) of 4T1 cells stained with fluorescence-labeled γ-H2AX second antibody with treatments of PBS (group Ⅰ), MCPNC14 (group Ⅱ), MCPNC14-FH2 (group Ⅲ), MCPNC14-FH2-IO3 (group Ⅳ), or MCPNC14-FH2-IO3@HA4 (group Ⅴ). *P < 0.05, **P < 0.01, ***P < 0.001.


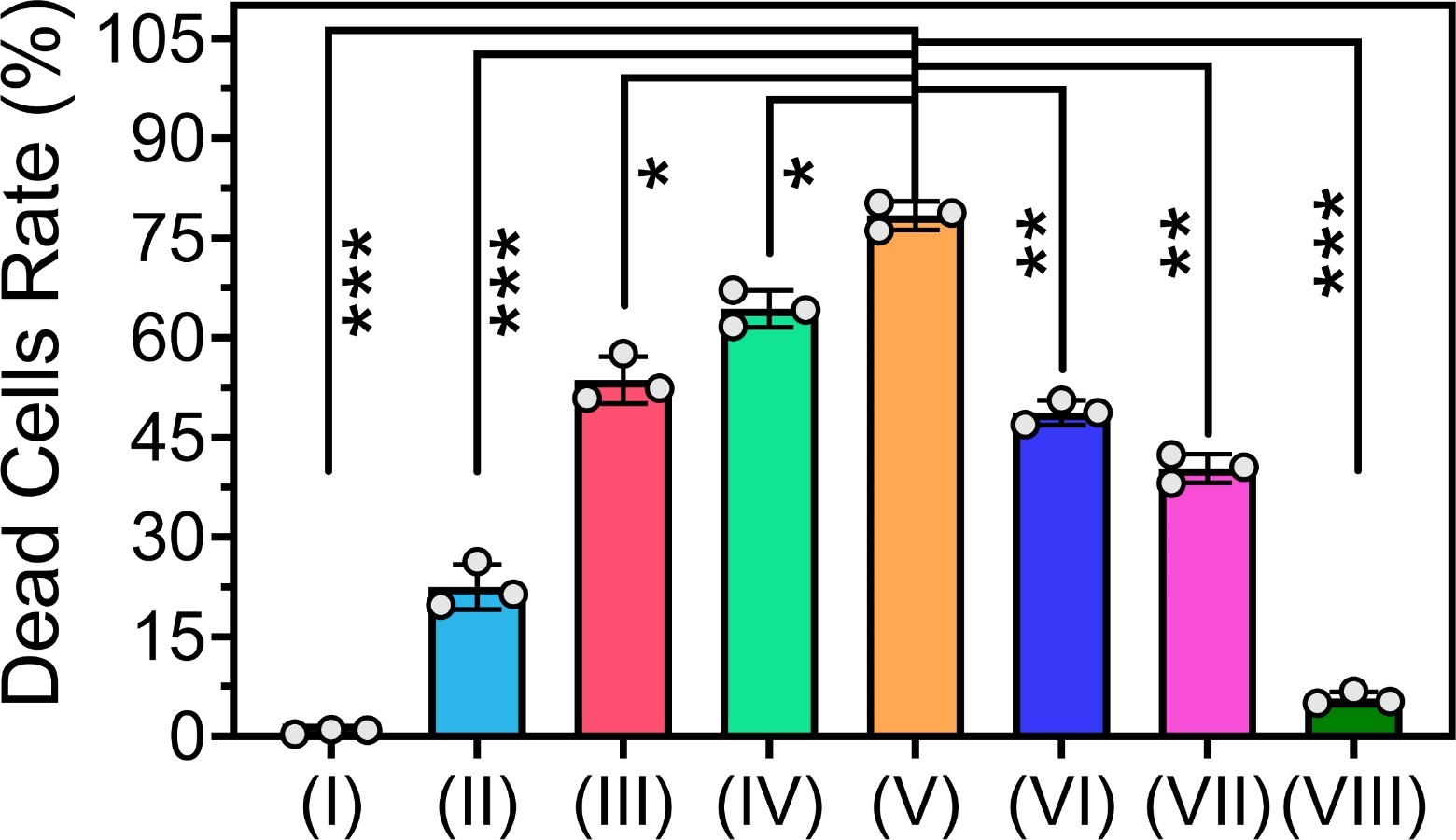


**Figure S28.** The corresponding quantitative analysis of dead cells rate measured by flow cytometry based on Calcein-AM and PI co-stained 4T1 cells after treatment with PBS (group Ⅰ), MCPNC14 (group Ⅱ), MCPNC14-FH2 (group Ⅲ), MCPNC14-FH2-IO3 (group Ⅳ), MCPNC14-FH2-IO3@HA4 (group Ⅴ), MCPNC14-FH2-IO3@HA4 plus DFO (group Ⅵ), MCPNC14-FH2-IO3@HA4 plus BAPTA-AM (group VII), or MCPNC14-FH2-IO3@HA4 plus both DFO and BAPTA-AM (group VIII). Mean ± S.D., *n* = 3. *P < 0.05, **P < 0.01, ***P < 0.001.


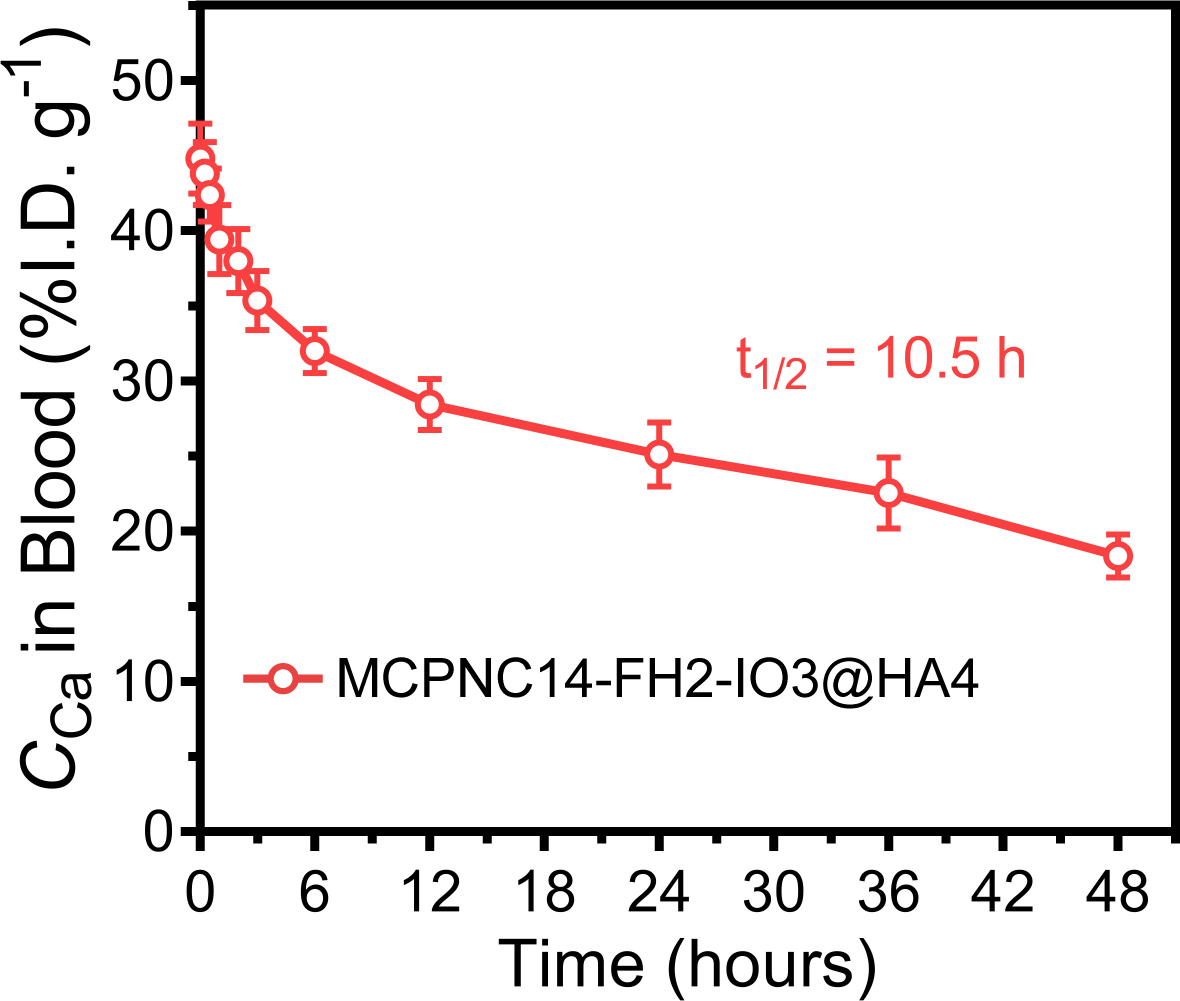


**Figure S29.** Pharmacokinetic profile of MCPNC14-FH2-IO3@HA4 in mice after intravenous injection (*C*_Ca_ = 5.0 mg/kg).


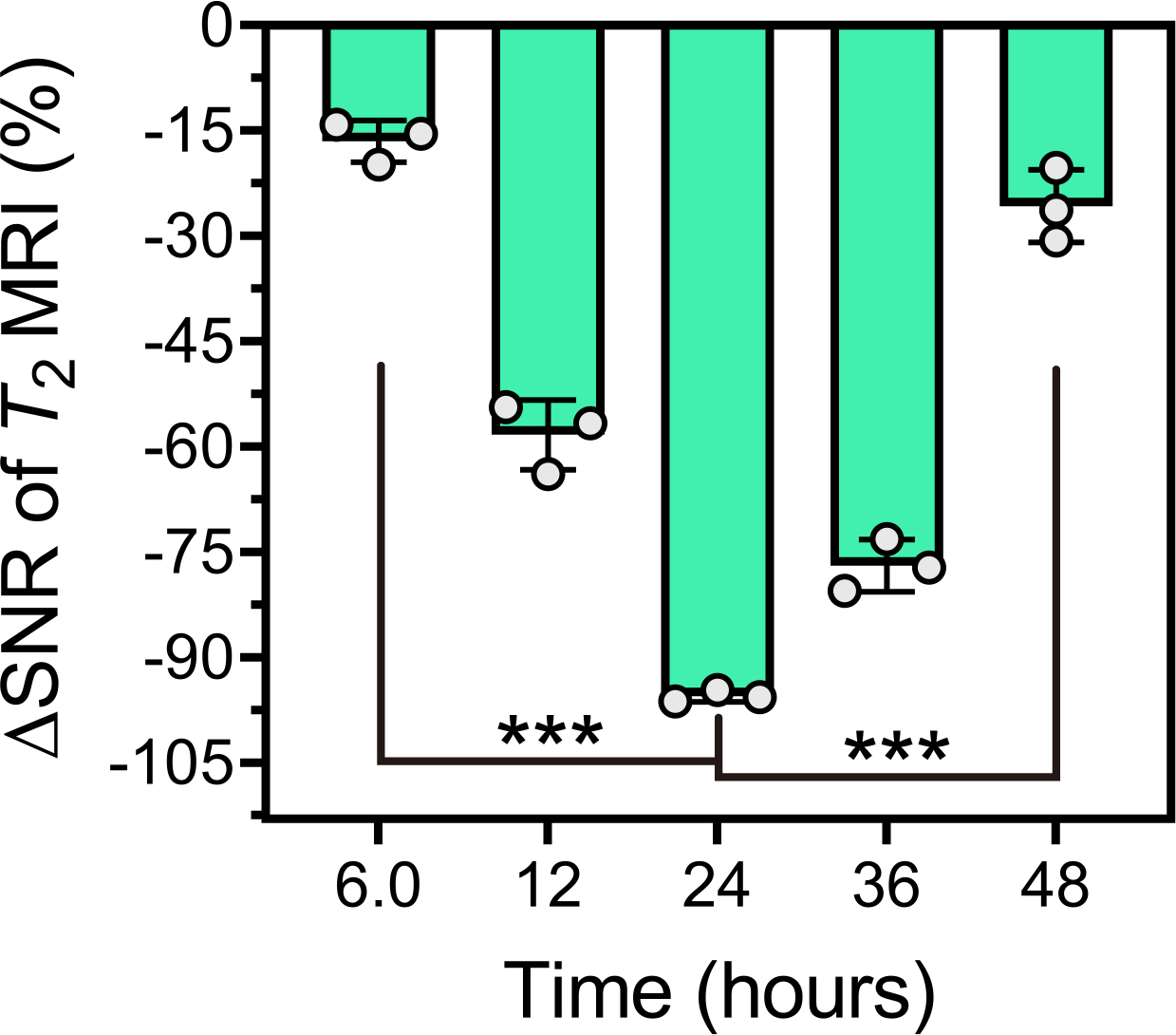


**Figure S30.** Quantitative measurement of the *T*_2_-weigted MR images for MCPNC14-FH2-IO3@HA4. Mean ± S.D., *n* = 3. ***P < 0.001.


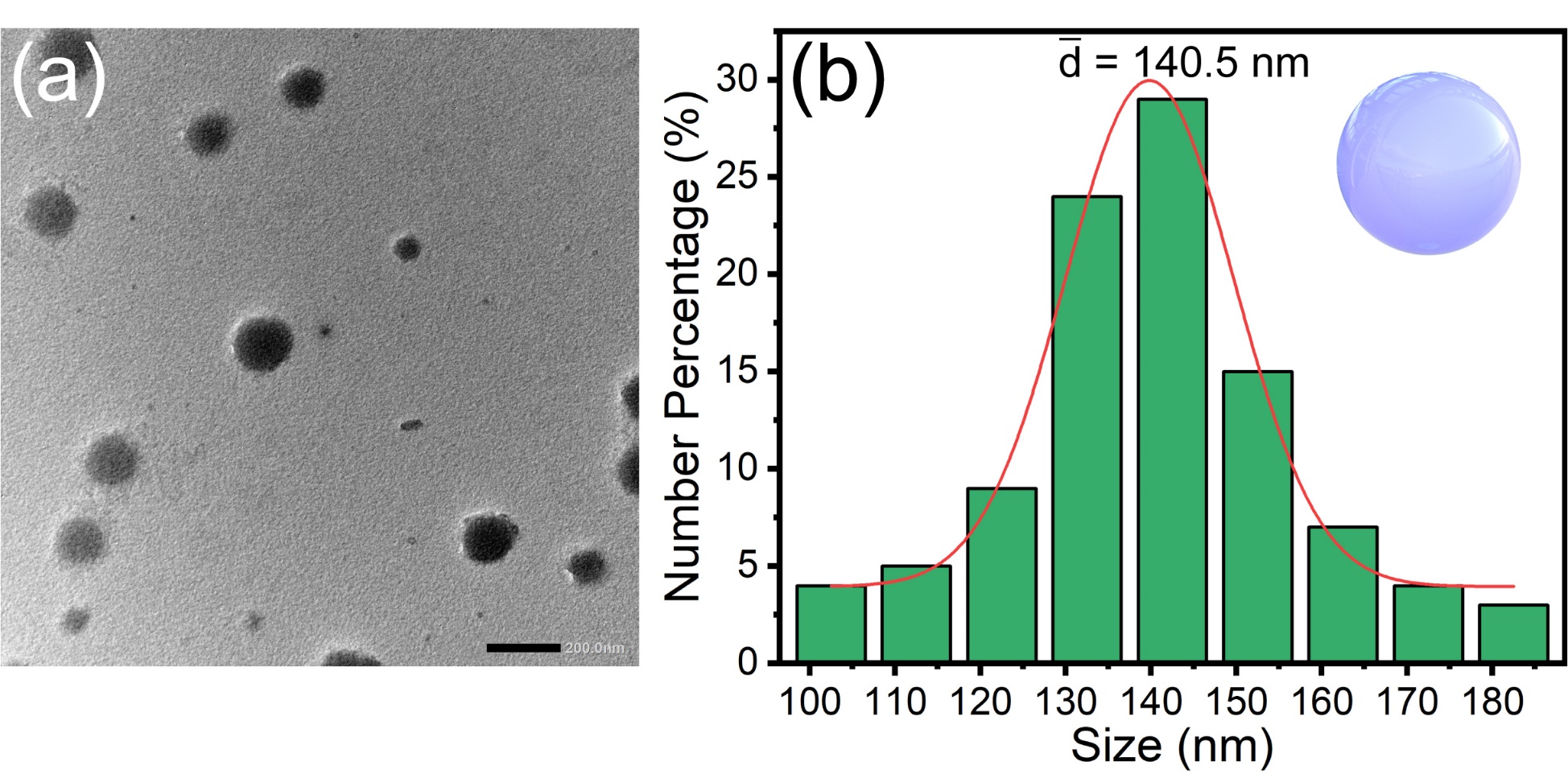


**Figure S31.** TEM image (a) and the corresponding particle size distribution (b) of CPNS. Scale bar = 200 nm.


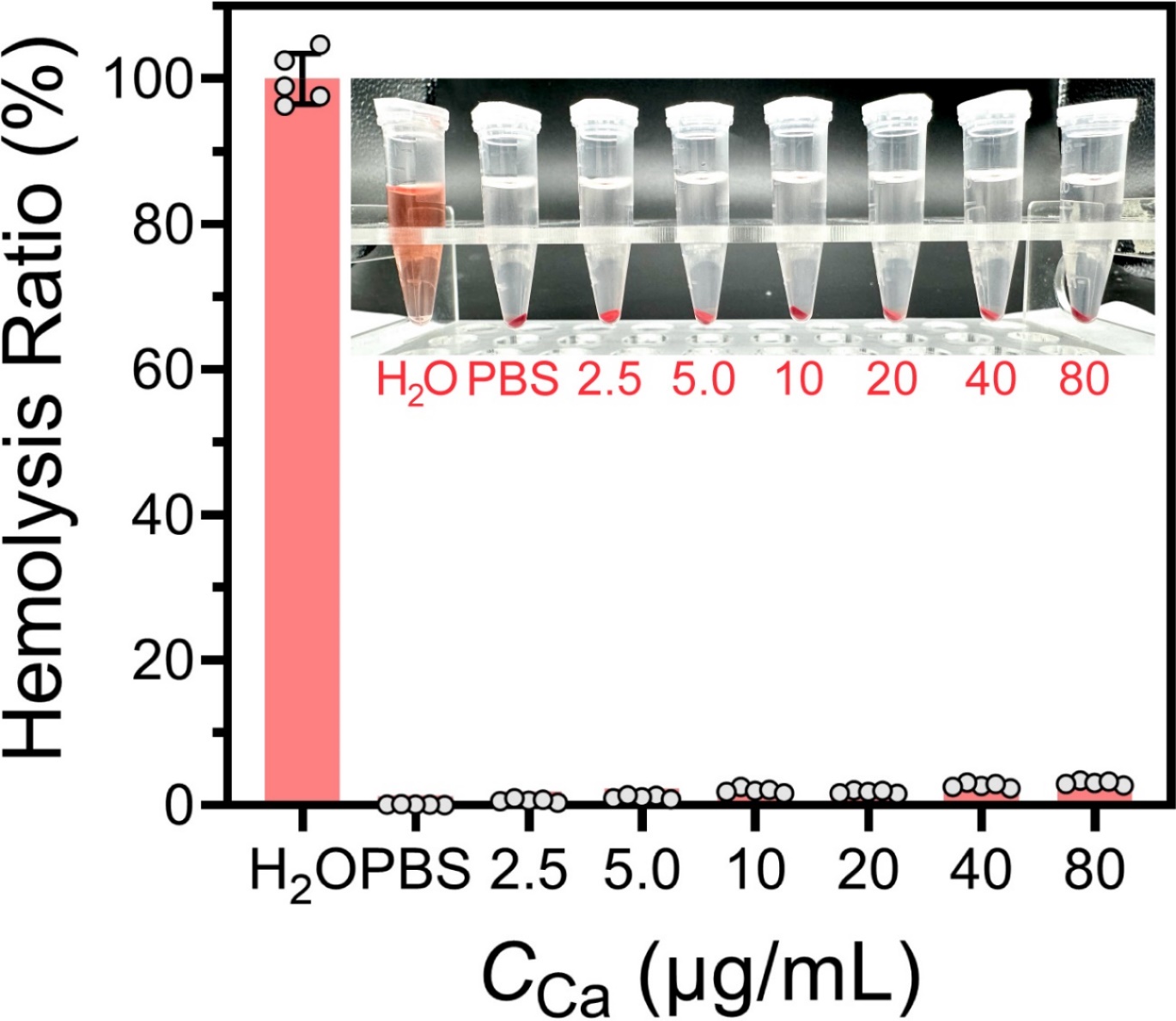


**Figure S32.** Quantitative analysis of hemolysis rates of ultra-pure water (H_2_O), PBS, and MCPNC14-FH2-IO3@HA4 with different *C*_Ca_ (2.5, 5.0, 10, 20, 40, or 80 μg/mL), accompanied by corresponding images of hemolysis. Mean ± S.D., *n* = 5.


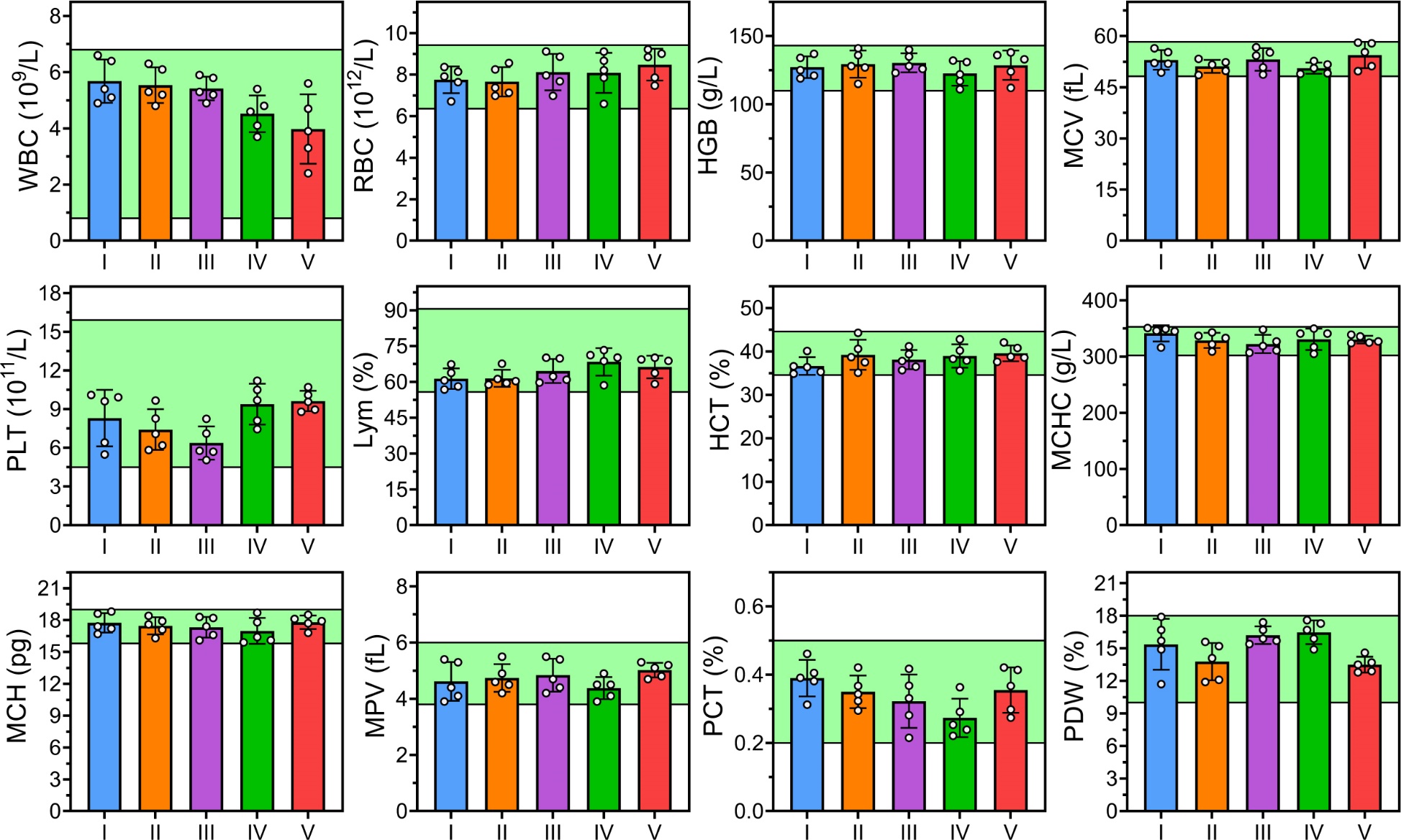


**Figure S33.** Routine blood analyses of healthy mice after intravenous injection of saline (group Ⅰ), MCPNC14 (group Ⅱ), MCPNC14-FH2 (group Ⅲ), MCPNC14-FH2-IO3 (group Ⅳ), or MCPNC14-FH2-IO3@HA4 (group Ⅴ) (Ca dosage = 5.0 mg/kg). The indicators for examination include: WBC, RBC, HGB, MCV, PLT, Lym, HCT, MCHC, MCH, MPV, PCT, and PDW. Mean ± S.D., *n* = 5. The normal parameter range for the indicator is marked as green area.
